# Nitrogen and phosphorous acquisition strategies drive coexistence patterns among archaeal lineages in soil

**DOI:** 10.1038/s41396-023-01493-y

**Published:** 2023-08-18

**Authors:** Jun Zhao, Laibin Huang, Seemanti Chakrabarti, Jennifer Cooper, EunKyung Choi, Carolina Ganan, Bryn Tolchinsky, Eric W. Triplett, Samira H. Daroub, Willm Martens-Habbena

**Affiliations:** 1 Fort Lauderdale Research and Education Center, Department of Microbiology and Cell Science, University of Florida, Davie, FL 33314 USA; 2 Everglades Research and Education Center, Soil and Water Sciences Department, University of Florida, Belle Glade, FL 33430 USA; 3 Department of Microbiology and Cell Science, University of Florida, Gainesville, FL 32611 USA

**Keywords:** Microbial ecology, Biogeochemistry

## Abstract

Soil represents the largest reservoir of *Archaea* on Earth. Present-day archaeal diversity in soils globally is dominated by members of the class *Nitrososphaeria*. The evolutionary radiation of this class is thought to reflect adaptations to a wide range of temperatures, pH, and other environmental conditions. However, the mechanisms that govern competition and coexistence among *Nitrososphaeria* lineages in soil remain poorly understood. Here we show that predominant soil *Nitrososphaeria* lineages compose a patchwork of gene inventory and expression profiles for ammonia, urea, and phosphate utilization. In contrast, carbon fixation, respiration, and ATP synthesis genes are conserved and expressed consistently among predominant phylotypes across 12 major evolutionary lineages commonly found in soil. In situ gene expression profiles closely resemble pure culture reference strains under optimal growth conditions. Together, these results reveal resource-based coexistence patterns among *Nitrososphaeria* lineages and suggest complementary ecophysiological niches associated with differential nutrient acquisition strategies among globally predominant archaeal lineages in soil.

## Introduction

Upland soils hold the largest global reservoir and a tremendous diversity of *Archaea* [1, 2]. This diversity is dominated by members of the archaeal class *Nitrososphaeria* [1–3]. Phylogenomic analyses identified 28 major putative family-level lineages within the *Nitrososphaeria*, of which 18, including the globally predominant lineages, are thought to oxidize ammonia [4–6]. Cultivated strains of this group couple the oxidation of ammonia to nitrite with autotrophic growth by carbon fixation via the modified 3-hydroxypropionate/4-hydroxybutyrate (HP/HB) pathway, and are often referred to as ammonia-oxidizing archaea (AOA) [2, 7, 8]. *Nitrososphaeria*-affiliated AOA often outnumber ammonia-oxidizing bacteria (AOB) and complete ammonia-oxidizing (comammox) bacteria [9–12]. However, many globally predominant AOA lineages in upland soil remain uncultured and their genomic inventory and ecophysiology remain poorly understood [6].

Oxygen availability, pH, and temperature have been important drivers of evolutionary diversification and habitat expansion within *Nitrososphaeria* over geological time scales [13–16]. Cellular ammonia affinities have also been shown to contribute to niche differentiation among cultivated AOA lineages with the highest affinities found in *Nitrosopumilus* and *Ca*. Nitrosotalea strains [17–21], and lower ammonia affinities found in terrestrial *Nitrososphaera* and *Ca*. Nitrosocosmicus strains [18, 19, 22]. Uptake and hydrolysis of urea and cyanate have been found in some cultivated AOA strains [23–26], and were suggested to provide additional niches within marine AOA assemblages [27–29]. Together, these factors could also play a significant role in the adaptation and coexistence of various *Nitrososphaeria* lineages in soil habitats. However, 11 of the 18 family-level AOA lineages remain uncultivated, including the numerically predominant lineages in soil, and the genetic and physiological mechanisms that drive competition and coexistence among these lineages in soil are poorly understood.

Metagenome-assembled genomes (MAGs) have provided important insights into the gene inventory and metabolic traits of terrestrial AOA lineages [5, 30–33]. However, due to the high microbial diversity within upland soil microbial communities and AOA assemblages within them, the soil AOA lineages are particularly underrepresented in current MAG collections. Only a few high-quality AOA genome reconstructions are currently obtained from individual grassland or cropland soils, and often stem from deeper layers (>20 cm) of the soil [30, 31]. Here, we report a combined biogeochemical and detailed paired metagenomic and metatranscriptomic analysis of AOA in an upland soil. The high relative abundance of AOA [9] and deep sequencing coverage enabled us to assemble MAGs, as well as to systematically assess the core gene inventory and gene expression of this nitrifier group across all predominant AOA lineages. The results reveal a complex assemblage of highly active AOA in soil affiliated with 12 major evolutionary lineages, and a pattern of nutrient acquisition-based coexistence among the predominant lineages.

## Materials and methods

### Site description and soil collection

Top 10-cm surface soil was collected from five differently managed soil plots in the Everglades Agricultural Area (EAA) in South Florida every 6–8 weeks between March 2017 and April 2018, including one unmanaged native soil (plot 1) and four agriculturally managed soils with different rotation regimes (plot 2–5). Details on soil collection, management, as well as physiochemical and biogeochemical activity measurements are described in Supplementary Materials and Methods, Supplementary Tables S1 and S2, and references [9, 34].

### Molecular analyses of soil microbial communities

Soil DNA and RNA extraction, PCR amplification and Illumina sequencing of 16S rRNA gene amplicons, and downstream data analysis via QIIME2 (version 2020.2) are described in detail in Supplementary Materials and Methods. All 16S rRNA amplicon sequence variants (ASVs) assigned to class *Nitrososphaeria*, excluding group 1.1c were considered as AOA, and the sum of ASVs classified as *Nitrosospira* sp. or *Nitrosomonas* sp. were considered as AOB. Other AOB genera were not detected. All ASVs of the genus *Nitrospira* were considered as the sum of comammox and canonical NOB (other NOB genera were not detected).

### Shotgun sequencing, metagenomic assembly and binning

Shotgun metagenomic and metatranscriptomic sequencing was conducted at the Department of Energy Joint Genome Institute (JGI) using a NovaSeq S4 instrument (see Supplementary Table S3 for detailed information). Genome assembly and binning was performed on each individual filtered metagenomic dataset, as well as by co-assembly using combined metagenomic datasets. Alternatively, each metagenome dataset was randomly subset to 20% of the original filtered read count for metagenomic assembly and binning. These approaches resulted in a total of ten non-redundant AOA MAGs with ≥80% completeness and <5% contamination. Comparative genomic analysis was conducted between 91 genomes and MAGs (including ten MAGs from this study and 81 from NCBI or IMG, Supplementary Table S4) to identify core protein clusters of AOA and lineage-specific protein clusters. Further details of genome assembly and binning, taxonomic and gene annotations, and comparative genomics are described in Supplementary Materials and Methods.

### Short read taxonomic classification and mapping of shotgun sequencing data

All filtered metagenomic or metatranscriptomic short reads were taxonomically and functionally annotated (e-value 10^–5^, 50% amino acid identity and 60% query coverage, see Supplementary Materials and Methods for further details). Read abundances were normalized by gene length and mapped read number (as reads per kilobase per million reads, RPKM). The 16S rRNA gene reads from metagenomic datasets were retrieved by SortMeRNA v4.0.0 [35] and taxonomically classified using a similar approach as for amplicon sequencing data.

To gain a more comprehensive understanding of the functional diversity of AOA and the key metabolic coding potential of different AOA, we performed additional analysis on selected genes of interest. All gene contigs (full-length or near-full length including ones that were not binned into MAGs) were screened for 26 marker AOA proteins, including DNA-directed RNA polymerase subunit B (RpoB), ammonia monooxygenase subunits (AmoABC and recently identified subunits AmoXYZ [36]), copper-containing nitrite reductase (NirK), acetyl-CoA carboxylase beta (AccB), malonic semialdehyde reductase (Msr), 4-hydroxybutyryl-CoA dehydratase (4HBD), cbb3-type cytochrome c oxidase subunit I (CoxA), putative cytochrome b/b6 domain protein (PetB), type 1 and 2 ammonium transporters (Amt-1 and Amt-2), type 1 and 2 sodium-solute symporters (SSS-1 and SSS-2), urea transporter (Ut), urease subunit alpha (UreC), nitrogen regulatory protein P-II (GlnB), phosphate uptake transporter permease protein A and C (PstA and PstC), phosphate transporter ATP-binding protein (PstB), low affinity inorganic phosphate transporter (PiT), ATP synthase subunit A (AtpA), and archaeal flagellin (FlaB). The retrieved sequences for each protein were dereplicated at 100% identity (for NirK at 80% identity due to high amino acid sequence variation) using CD-HIT v4.6.8 [37], and used to establish an in-house database also containing the homologous non-redundant protein sequences from publicly available reference AOA genomes and the ten MAGs obtained in this study. Such cutoff roughly represents genus- or species-level diversity, and we defined each unique protein sequence as “phylotype” throughout the paper. Sequences from each in-house database were aligned with MAFFT 7.294 [38] and trimmed by trimAl 1.2 [39]. Maximum likelihood phylogeny was constructed by IQ-TREE 2.1.0 [40] using best fit substitution model and 1000 bootstrap iterations and visualized on iTOL (https://itol.embl.de/) [41]. Each protein sequence assembled from our soils was then taxonomically classified at family level according to the identity with reference genome protein sequences, with taxonomic placement of some sequences further manually corrected based on phylogenetic tree topology and clustering. Unassembled short reads from metagenomic and metatranscriptomic datasets were mapped against the in-house protein databases using Diamond blastx (e-value 10^−5^, 90% amino acid similarity and 60% query coverage) to generate the abundance information (RPKM values). Additionally, metatranscriptomic reads were mapped against the ten MAGs from this study using bowtie2 v2.4.5 [42] to generate RPKM values of all genes in each MAG.

### Statistical analyses

To validate family- and order-level taxonomic classification, the normalized abundance of the 26 marker genes in each specific lineage in each soil was compared. The ratio of each target gene abundance relative to *rpoB* (a single-copy gene for all AOA) gene abundance within each AOA lineage was calculated, and expected to approach value of one for core gene commonly present as single copy in AOA genomes (e.g. *amoAB*, *4hbd*, *accB*, *pstABC*, and *coxA* genes), to be higher than one for often multi-copy genes (e.g. *amoC*, *nirK*, *amt*, and *glnB* genes), and to be less than one for genes that were not always present in AOA cells (e.g. *ut*, *ureC*, and *flaB* genes). Because *rpoB* is a housekeeping gene for RNA synthesis and constitutively expressed in living cells, its transcript abundance in AOA was further used as a proxy to assess the transcriptional profile of other functional genes. For comparison of gene expression profiles of soil AOA lineages and laboratory strains, the ratios of normalized target gene transcript abundance relative to *rpoB* transcript abundance within each AOA lineage (after summing the abundance of all phylotypes from each lineage) in soils and three AOA strains (i.e. *Nitrosopumilus maritimus* SCM1, *Ca*. Nitrosopelagicus brevis U25, and *Nitrososphaera viennensis* EN76) were calculated.

## Results

### *Nitrososphaeria* AOA dominate archaeal and nitrifier assemblage throughout the year

**Fig. 1:**
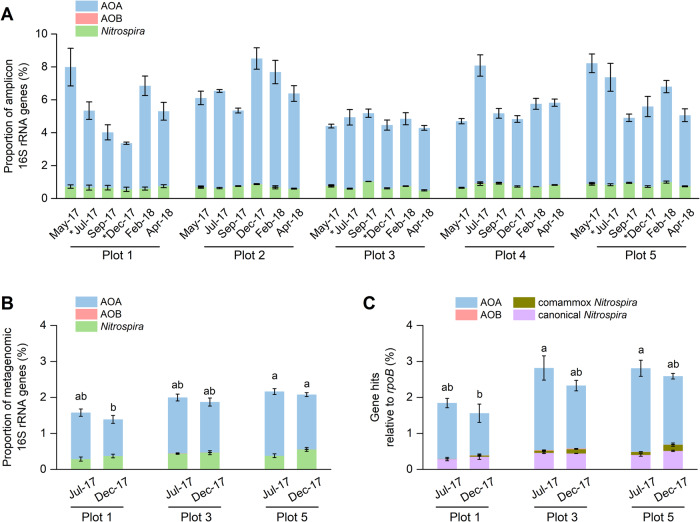
Proportions of nitrifiers in different soils plots. **A** Stacked columns represent proportions of AOA, AOB, and total *Nitrospira* based on 16S rRNA genes from amplicon sequencing. **B** Proportions of AOA, AOB, and total *Nitrospira* based on 16S rRNA genes from metagenomes. **C** Proportions of AOA, AOB, comammox, and canonical *Nitrospira* based on normalized abundances of functional genes of nitrifiers, relative to *rpoB* genes of all prokaryotes. The proportions of AOA, AOB, and comammox abundances were estimated by normalized abundance of *amoA* genes and the canonical *Nitrospira* abundances were estimated by calculating the differences of normalized abundances of *nxrB* genes (total *Nitrospira*) and *amoA* genes of comammox *Nitrospira*. The abundances were also normalized by gene length and are based on the assumptions, that each prokaryotic genome contains one *rpoB* gene copy, AOA cells possess one *amoA* gene copy, AOB cells possess 2.5 *amoA* gene copies, comammox cells possess 1.5 *amoA* gene copies, and *Nitrospira* cells possess 4 *nxrB* gene copies. Error bars represent standard errors of relative abundances of triplicate samples and different letters above columns indicate significant difference between plots and seasons.

*Nitrososphaeria* AOA represented 79.4–100%, 75.4–98.0%, and 73.4–98.6% of total archaea and 2.8–7.6%, 1.0–1.8%, and 1.2–2.3% of total microbial communities in 16S rRNA gene amplicon, metagenome-recovered 16S rRNA genes, and prokaryotic *rpoB*-normalized *amoA* gene reads, respectively, throughout the year (Fig. 1A–C). Non-ammonia-oxidizing *Nitrososphaeria* lineages were not detected. Among other nitrifiers, *Nitrospira* sp., including canonical NOB and comammox, made up 0.5–1.0%, 0.3–0.6%, and 0.3–0.7% of 16S rRNA gene amplicons, metagenome-recovered 16S rRNA genes, and *rpoB*-normalized nitrite oxidoreductaste (*nxrB*) genes, respectively. Relative abundance of AOB was consistently at or below the detection limit (≤0.02%) in all three approaches. Relative abundance of comammox *Nitrospira* based on *amoA* was estimated to be between 0.004–0.2% of total prokaryotic communities (Fig. 1C), equivalent to 1.5–24.7% of total *Nitrospira* (Supplementary Table S5). Similar relative abundance estimates for AOB (≤0.02%) and comammox (≤0.2%) were also obtained from *rpoB*-normalized hydroxylamine oxidoreductase (*hao*) gene abundances (Supplementary Table S5). It is noteworthy that the comammox to total *Nitrospira* proportion might be lower or higher than our estimate, since the *nxrB* gene copy number in *Nitrospira* cells can be variable.

Although total microbial and AOA community composition varied to a certain degree over time, the differences between plots were larger than between seasons (Supplementary Figs. S1 and S2). Soil pH was most likely the primary factor determining differences in AOA composition between different plots (*R*^*2*^ > 0.92, *p* < 0.001), based on combined analysis of multiple environmental parameters (Supplementary Fig. S1) and results from our previous study [9]. Especially the AOA composition in the unmanaged soil (plot 1, pH 5.0–5.8) differed from profiles in the agricultural soils (pH 6.3–7.9). Ammonia and urea concentrations were low in all plots throughout the year, except in plot 1 after mowing in April 2017 (Supplementary Fig. S3, Supplementary Table S6). Available phosphorus concentration was consistently lower in the unmanaged soil than that in agricultural soil (Supplementary Fig. S3). The most contrasting geochemical conditions were related to precipitation and soil temperature between seasons (Supplementary Fig. S3). Plots 1, 3, and 5 were therefore selected for further detailed metagenomic and metatranscriptomic analysis of AOA gene inventory and expression in both warm, wet summer and dry, cooler winter season (more description in Supplementary Results).

### High diversity of AOA across three main orders of *Nitrososphaeria*

Metagenome assembly and binning yielded ten non-redundant AOA MAGs passing quality filtering (79.8–98.5% completeness and 0–4.4% contamination, Supplementary Table S7), all belonging to the order *Nitrososphaerales*. Nine MAGs were obtained from the agricultural soils belonging to four family-level AOA lineages. Based on whole genome phylogeny, these included four *Nitrososphaeraceae* (also known as NS-alpha) MAGs, three NS-delta MAGs, one NS-beta MAG, and one NS-epsilon MAG (Supplementary Fig. S4). One additional MAG was obtained from the unmanaged soil, which was distantly related to NS-beta (hereafter assigned as “NS-beta-associated”). Although some of these MAGs represented a predominant AOA lineage in the respective soil, they did not cover the large diversity of AOA in these soils.

**Fig. 2:**
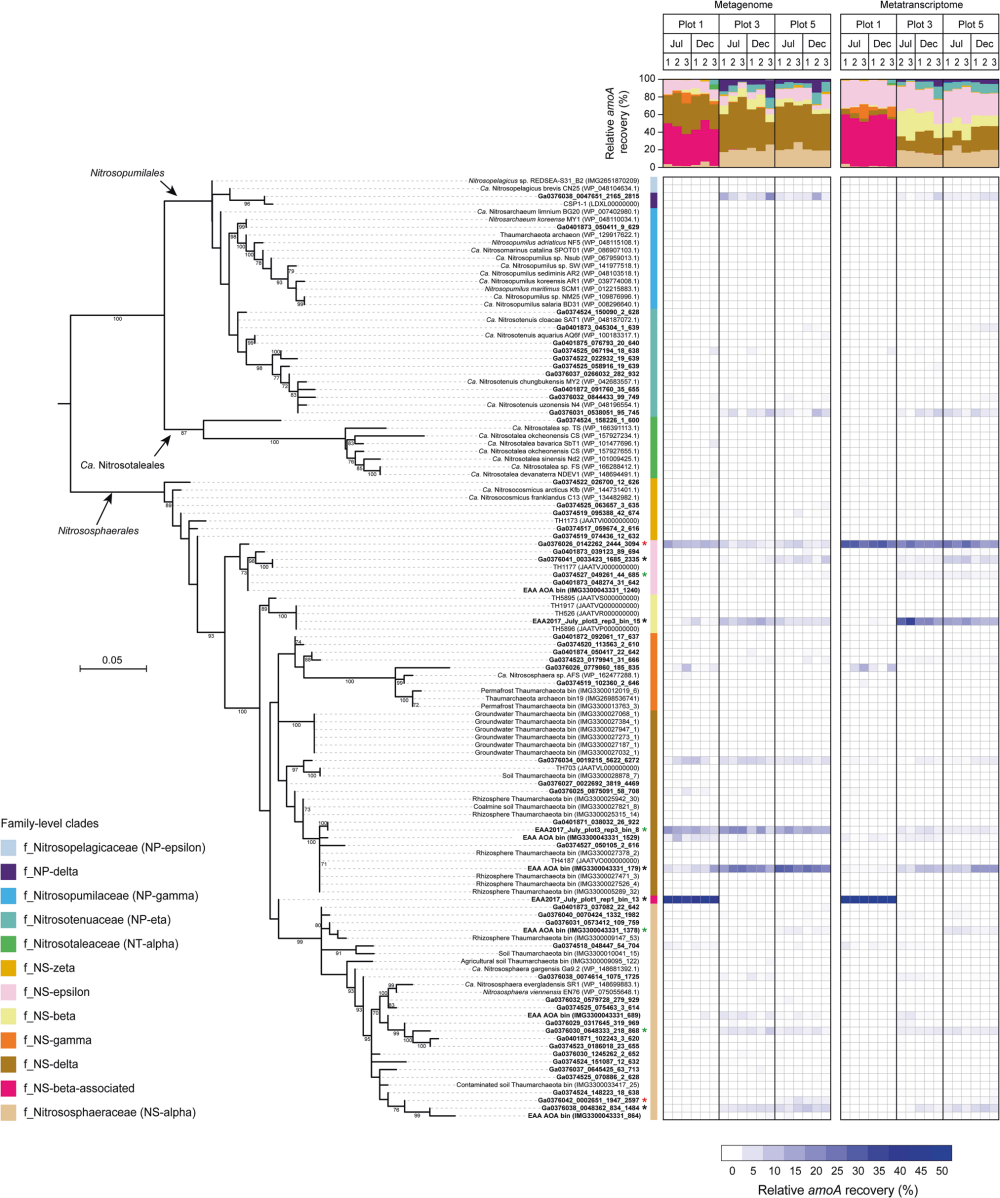
Maximum likelihood phylogeny of AOA AmoA protein sequence assemblies recovered from metagenomes and metatranscriptomes in this study (left) and heat map of relative abundance based on metagenome and metatranscriptome read mapping (right). Representative sequences assembled in this study are indicated by their IMG accession number (“Ga*”). Reference genome sequences of cultured AOA and MAGs retrieved from NCBI RefSeq and IMG databases were used to classify each phylotype to a family-level AOA lineage based on genome-wide taxonomic ranking [5]. The AmoA of the ten MAGs from this study was also included in the phylogeny. Ultrafast bootstrap values > 70% (1000 replicates) are indicated and the scale bar represents 0.05 substitutions per amino acid position. Heat map shows the proportion of mapped sequencing reads of each phylotype relative to total AOA *amoA* reads in each metagenome or metatranscriptome. Shown are all three biological replicates, except for plot 3, for which only two metatranscriptomes were recovered in July 2017. The asterisk (*) symbols refer to specialized phylotypes with corresponding proportions of gene (red asterisk), transcript (green asterisk), or both (black asterisk) abundances significantly affected by different land-use types. Stacked columns above the heat map show the proportion of each AOA family in individual metagenome or metatranscriptome.

To generate more comprehensive profile of AOA diversity, we reconstructed the phylogeny based on protein biomarkers. Metagenome-assembled AmoA protein sequences revealed a total of 60 unique AOA phylotypes, with higher richness in the two agricultural soils (55 and 49 phylotypes in plots 3 and 5, respectively) than in the unmanaged soil (plot 1, 40 phylotypes) (Fig. 2). In line with the high congruence between *amoA* and genome-based phylogeny of AOA [5], all Amo phylotypes clustered into 11 of the 18 known family-level AOA lineages, as well as the one additional putative NS-beta-associated lineage, of the three main orders of *Nitrosopumilales*, *Ca*. Nitrosotaleales, and *Nitrososphaerales* (Fig. 2). The NS-beta-associated lineage was almost exclusively found in the low pH plot 1. Mapping of short metagenome reads against the AmoA sequences revealed that *Nitrososphaerales* families dominated AOA gene abundances in all soils (84.6–98.1%), with the main compositional shift between unmanaged low pH plot 1 and agricultural plots 3 and 5 (Fig. 2, Supplementary Table S8). The unmanaged plot 1 with lower soil pH was co-dominated by NS-delta (33.2% ± 1.8%) and NS-beta-associated AOA (42.7% ± 1.7%), whereas in the two agricultural plots (plots 3 and 5), NS-delta (46.0% ± 2.5%) and *Nitrososphaeraceae* (21.2% ± 0.9%) were predominant. NS-beta (2.4% ± 1.0% and 7.9% ± 1.0% in unmanaged and agricultural soils, respectively) and NS-epsilon (12.1% ± 0.9% and 9.5% ± 1.2% in unmanaged and agricultural soils, respectively) were also abundant. The two *Nitrosopumilales* families, NP-delta and *Nitrosotenuaceae* (also known as NP-eta), were also present at relatively high frequency in plots 3 and 5 (14.2% ± 2.4%), but were scarcely detected in the unmanaged soil (0.9% ± 0.9%).

### Inventory and phylogenetic congruence of key metabolic proteins in soil *Nitrososphaeria* assemblage

To capture the core metabolism of AOA, especially family-level lineages not represented by our MAGs, phylogenies of 25 additional key proteins within the known genetic inventory of AOA were reconstructed from our metagenome datasets and assigned to all different AOA lineages in our soils. The selected genes encode proteins for RNA synthesis, uptake and metabolism of nitrogen, carbon, and phosphorous (Supplementary Figs. S5–S27). Phylogeny of most single-copy core proteins supported the high diversity of AOA, e.g., AccB, 4HBD, PetB, and RpoB, with the number of detected phylotypes ranging from 35–127, together indicating a robust and sensitive detection of all 12 AOA families in these soils (see Supplementary Figs. S5, S11–S15, S21–S23, and S27). As expected, phylogenies of these proteins were highly congruent with AmoA phylogeny, confirming previous studies demonstrating that evolution of the class *Nitrososphaeria* is predominantly characterized by radiative diversification and gene duplications with limited horizontal gene transfer [5, 43]. This congruence was further supported by similar read recovery (RPKM) of *amoA* and these single-copy genes in the same lineage (Supplementary Table S9). However, within the selected key protein inventory, NirK, Amt, SSS, UreC, GlnB, and ATPases proteins did not follow order-level radiative diversification (Supplementary Figs. S10, S16, S17, S19, S20, S25, and Supplementary Results), likely due to horizontal gene transfer or presence of multiple homolog genes in individual genomes [33]. Although these protein phylogenies were not always consistent with AmoA phylogeny, distinct family affiliations could still be properly assigned using these proteins based on high sequence similarity to those of known genomes and MAGs, as well as metagenomic read mapping statistics (Supplementary Table S9). Based on the normalized abundance of AOA-derived *ureC* genes relative to *amoA* and *rpoB* reads, on average 85.7–89.8% of the AOA in our soils encoded urease, a much higher frequency than previously reported for marine AOA [28], and indicated that capability for urea utilization is widespread among upland soil AOA. The *ureC* gene was present in all *Nitrososphaerales* lineages. We further detected urea transporter genes (*ut*) in all *Nitrososphaerales* families, except NS-delta, despite the presence of urease in this lineage.

### Lineage-specific protein clusters identified by comparative genomics

To investigate the protein inventory beyond the 26 proteins analyzed above, a comparative genomic analysis was conducted using 91 available AOA genomes or MAGs obtained from this study and publicly available datasets, representing four major AOA linages at order level. We detected a total of 27,047 distinct protein clusters, 915 were recognized to constitute the AOA core proteome in this study (present in at least one genome of each AOA order, Supplementary Table S10). We then characterized the lineage-specific protein clusters that were uniquely present in at least two MAGs of the same AOA family (except for NS-beta-associated with only one MAG). A total of 695 (160 functionally annotated), 490 (72), 958 (63), 859 (123), and 280 (28) of the protein clusters were uniquely present in the *Nitrososphaeraceae*, NS-beta, NS-beta-associated, NS-delta, and NS-epsilon lineages, respectively (Supplementary Table S11), to which our ten MAGs were affiliated.

Analysis of the annotated protein clusters suggests some lineages can potentially utilize specific exogenous organic compounds. Two of the NS-delta MAGs (include one from this study) contained phylogenetically unique protein clusters (cluster-14563, cluster-15305, Supplementary Table S11) annotated as aspartate ammonia-lyase. Although another aspartate ammonia-lyase protein cluster (cluster-842) was present in all lineages of AOA, the extra copies of the phylogenetically distinct aspartate ammonia-lyase gene in only NS-delta MAGs could suggest an increased need for aspartate degradation yielding additional ammonia, or alternative substrate spectrum. Furthermore, a phylogenetically distinct lactate racemase cluster (cluster-12894) was exclusively detected in all 19 NS-delta MAGs analyzed in this study, suggesting the ability to utilize both lactate stereoisomers, a specific need for interconversion of these isomers, or alternative substrate spectrum of this enzyme, is a ubiquitous and essential trait within this lineage. NS-beta contained a unique cluster of pyruvate kinase (cluster-15605), which could facilitate utilization of additional nitrogen and carbon source. Experimental validation of these enzymes will be required to further elucidate their potential function in this lineage.

Unique genetic traits were identified potentially for adaptation to limited nutrient availability and other environmental stresses. Three of the four NS-epsilon MAGs (including the one assembled from our soil) contained both V-type and A-type copies of ATPase genes (Supplementary Fig. S25). Coexistence of V- and A-type ATPase was previously detected in single deep-sea *Nitrosopumilales* MAG (from NP-iota and NP-theta [44]), and was reported first time here in individual *Nitrososphaerales* MAGs. As V- and A-type ATPase were suggested to play a role for adaptation to acidophilic/piezophilic and neutrophilic environments, respectively [33], the coexistence of these two types of ATPase within NS-epsilon may facilitate its adaptation to a wide range of soil pH. The NS-beta-associated MAG exclusively detected in the low pH plot was the only genome possessing four copies of ammonium transporter genes (two *amt-1* and two *amt-2* genes), which might provide additional advantage for taking up ammonium in low pH soil where the free ammonia availability is limited [45]. This MAG also contained many protein clusters that may regulate pH homeostasis (Supplementary Table S11), such as cation/proton antiporter (cluster-754, cluster-18765, cluster-19192, cluster-21479), major facilitator superfamily (MFS) transporters (cluster-731, cluster-17003, cluster-25995, cluster-26005, cluster-26215, cluster-26425, cluster-26868), resistance-associated macrophage protein (NRAMP) divalent cation transporters (cluster-16976), P-type ATPases (cluster-13967, cluster-14689, cluster-24153), and arginine decarboxylase (cluster-17271), which function as ion transporter or proton scavenger. However, most of these protein clusters were also detected in neutrophilic AOA as shown previously [46]. Nevertheless, the NS-beta-associated MAG possessed a potassium transporter (TrkA) cluster (cluster-13620) different from all the other AOA lineages. It also shared a unique cluster of NhaP-type cation/proton antiporter (5 gene copies in this MAG, cluster-26925), NRAMP divalent cation transporter (cluster-22662), and carbonic anhydrase (for interconversion of HCO_3_^−^ and CO_2_, cluster-26023) with NS-gamma, NS-zeta, and *Nitrosotaleaceae* genomes which were previously suggested to aid their adaptation to low pH [21, 33].

### The majority of *Nitrososphaerales*-affiliated AOA were transcriptionally active

**Fig. 3:**
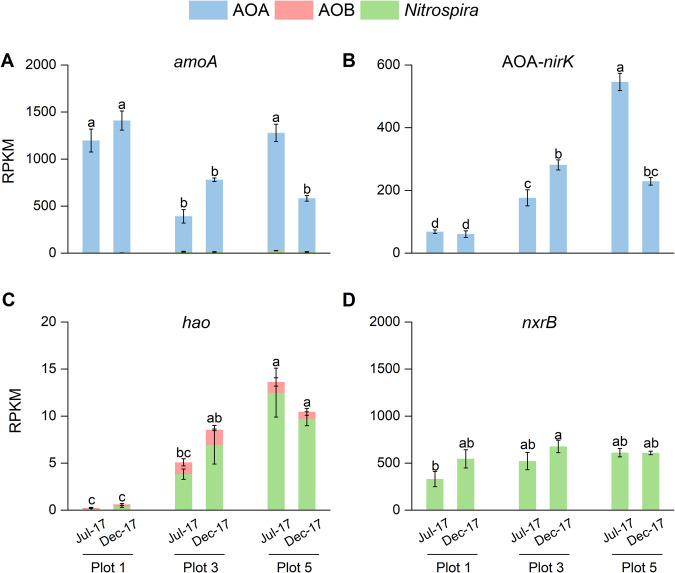
Transcriptional activities of nitrifiers based on normalized abundances of *amoA*, *nirK*, *hao*, and *nxrB* gene transcripts. **A** Stacked columns represent abundances of *amoA* transcripts from AOA, AOB, and comammox *Nitrospira*. **B** Columns represent *nirK* transcript abundances of AOA. **C** Stacked columns represent *hao* transcript abundances of AOB and comammox *Nitrospira*. **D** Columns represent *nxrB* transcript abundances of *Nitrospira*. *amoA*, ammonia monooxygenase; *nirK*, copper containing nitrite reductase; *hao*, hydroxylamine oxidoreductase; *nxrB*, nitrite oxidoreductase. Error bars represent standard errors of relative abundances of triplicate samples (except for two replicates in July-17 metatranscriptomes) and different letters above columns indicate significant differences between plots and seasons.

The viability and activity of *Nitrososphaeria* AOA was first assessed at the whole microbial community level. Many genes for nitrogen and carbon metabolism of AOA were among the most highly expressed microbial genes in the soils. These included *amoABC*, *nirK*, *amt-*1 (high-affinity ammonium transporter), *ureA*, *ureC*, *4hbd*, and *accB* genes (see Supplementary Fig. S28 and Supplementary Table S12). Archaeal *amoA* accounted for 95.1–100% of detected *amoA* transcripts in all soils with RPKM values between 373–1407 (Fig. 3A). Transcript read mapping revealed that all 60 AOA phylotypes detected in the *amoA* gene set were transcriptionally active in at least one soil, suggesting that a viable ecological niche existed for all detected AOA in our soils. However, transcript to gene ratios of some AOA phylotypes were consistently lower than in others. NS-delta, one of the most abundant AOA lineages in all three plots, accounted for 31.7%–52.0% of total archaeal *amoA* genes in metagenomes, but for only 4.6%–27.4% of *amoA* in metatranscriptomes (top stacked column in Fig. 2 and Supplementary Fig. S29). Archaeal *nirK* genes were expressed with RPKM values between 61–546 at whole AOA community level (Fig. 3B), corroborating the importance of the *nirK* in the archaeal nitrogen metabolism in soil. Based on *amoA* and *nirK* gene transcript abundance, AOA were similarly active in summer and winter in the unmanaged plot 1, but were less active in July than in December in plot 3, and conversely more active in July than December in plot 5, in line with the contrasting in situ CO_2_ fluxes between plots 3 and 5 (Supplementary Fig. S3). Archaeal *nirK* transcript abundances closely mirrored *amoA* transcript abundances in plots 3 and 5 with a steady *amoA* to *nirK* transcript ratio of approximately 2:1. This ratio was higher (22:1) in unmanaged plot 1, suggesting that *nirK* expression was less important at the lower pH.

Other highly transcribed genes included cbb3-type cytochrome c oxidase subunit I (*coxA*) and archaeal flagellin (*flaB*), even though the latter were only found in 4 AOA families (Supplementary Fig. S26). The multicopper oxidases genes, i.e. MCO1 and MCO4 of AOA [47], previously shown to be responsive to Cu availability in pure culture and proposed to be involved in Cu binding and uptake [48], were recovered at similar frequencies as AOA *nirK* genes, but were rarely transcribed (Supplementary Table S12), suggesting that copper may not have been a limiting factor for AOA in situ.

**Fig. 4:**
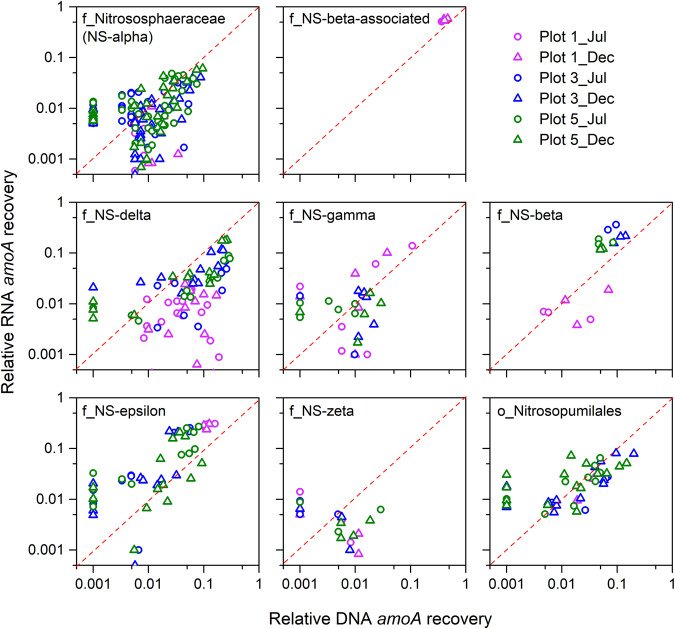
Recovery frequencies of *amoA* reads of AOA phylotypes relative to total archaeal *amoA* read numbers from metagenome and metatranscriptome data. A symbol (circle or triangle) in each plot represents the relative *amoA* DNA and RNA recovery frequencies of an AOA phylotype (100% protein sequence identity). The red dotted line across each plot indicates the same recovery frequency at both DNA and RNA levels, reflecting activity of a phylotype proportionate to corresponding relative abundance.

Relative activity of the individual AOA phylotypes was evaluated via ratio of *amoA* transcript reads versus *amoA* gene reads, similar to an approach using rRNA transcripts versus gene ratio as indicator of prokaryotic activity and dormancy [49, 50]. The results revealed that all or most phylotypes of NS-epsilon (81%), NS-beta (82%), and NS-beta-associated (100%), about half of the *Nitrososphaeraceae* (43%), NS-gamma (53%), and NS-zeta (43%) phylotypes, as well as *Nitrosopumilales* (50%) phylotypes were highly active, that is, displayed >1:1 transcript-to-gene read recovery ratios (Fig. 4). In contrast, the majority of NS-delta phylotypes (84%) showed relatively low *amoA* gene expression activity, especially in unmanaged plot 1 (transcript-to-gene read recovery ratios <1:1, Fig. 4). Similarly low transcript-to-gene recovery ratios for NS-delta were observed based on *rpoB* gene (71% <1:1 ratio, Supplementary Fig. S30) and CO_2_ fixation gene (*accB*, 86% <1:1 ratio, Supplementary Fig. S31). Taken together, these data demonstrate widespread and consistent transcriptional activity among AOA phylotypes of all families detected in the metagenomes. Relatively low transcript to gene ratios across nitrogen, carbon metabolism, and RNA synthesis in NS-delta phylotypes suggest a smaller active fraction of NS-delta cells than in other families, and that neither of the seasons and field management conditions may have been optimal for members of this lineage at the time of sampling.

Transcriptional activity of AOB and comammox were low in all three plots with RPKM values 0.5–5.1 for AOB, and 0.3–25.1 for comammox *amoA* transcripts, respectively (Fig. 3A), much lower than AOA *amoA* transcripts. Transcripts of *hao* were similarly low with RPKM values of 0.1–1.6 and 0.1–12.5 for AOB and comammox, respectively (Fig. 3C). In addition, highly transcribed genes of AOB were all housekeeping and regulatory proteins (e.g., ribosomal proteins, stress response proteins, and chaperones), whereas *amoABC* and *hao* operons were minimally transcribed (Supplementary Table S13). Nitrite oxidoreductase (*nxrB*) was among the highest expressed genes within *Nitrospira* sp., but *Nitrospira amoA* and *hao* transcripts were detected at orders of magnitude lower levels than *nxrB* (Supplementary Table S14). Together, these data indicated that all AOA phylotypes were active in situ, expressing pathways for ammonia and urea uptake and metabolism, as well as CO_2_ fixation. In contrast, comammox bacteria and AOB may not have been significantly engaged in ammonia oxidation, but both still remained viable and transcriptionally active. Although low transcript abundances do not directly equate to low catalytic activities, the low gene and transcript abundances of AOB and comammox further corroborate our previous results that even under optimal growth conditions both groups likely contributed minimally to ammonia oxidation in these soils [9]. Nevertheless, both comammox *amoA* and *hao* gene transcripts were detectably higher in agriculturally managed plots 3 and 5 than in the unmanaged plot 1 (Fig. 3A, C). In contrast, we found *Nitrospira*-affiliated *nxrB* gene transcripts (RPKM values of 336–696) at levels similar to archaeal *amoA* transcripts in all three plots, indicating in situ coupling of archaeal ammonia oxidation and *Nitrospira* sp.-mediated nitrite oxidation (Fig. 3D).

### Gene expression profiles of AOA in soil resemble pure culture reference strains

**Fig. 5:**
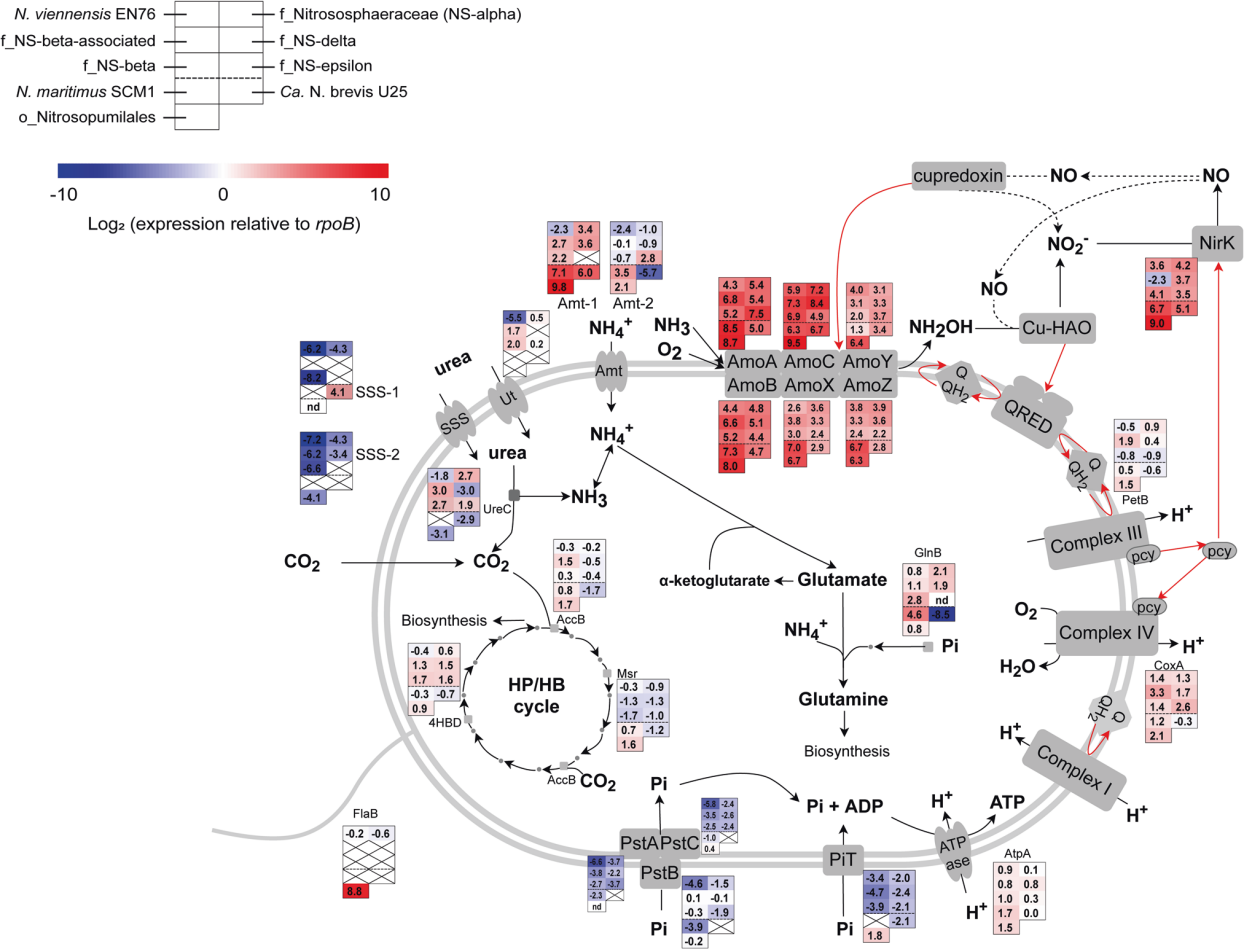
The *rpoB*-normalized transcript abundance of core metabolism pathway genes in six major AOA lineages (*Nitrososphaeraceae*, NS-beta-associated, NS-delta, NS-beta, NS-epsilon, and the order-level lineage *Nitrosopumilales*) from EAA soils. Normalized transcript abundances of three pure culture strains during ammonium-replete (*Nitrososphaera viennensis* EN76 and *Nitrosopumilus maritimus* SCM1) or urea-replete (*Ca*. Nitrosopelagicus brevis U25) exponential growth from previous studies are given for comparison [48, 51, 52]. The cell diagram shows the core AOA metabolic pathways and proteins for ammonia transport, oxidation and assimilation, electron transfer, HP/HB carbon fixation, and ATP synthesis. The heatmap shows log_2_-transformed ratios of target gene transcript abundance to *rpoB* transcript abundance within each lineage averaging different plots and seasons. Cross mark “X” indicates gene absent in a lineage or strain; “nd” indicates gene present, but no transcripts detected in metatranscriptomes. Red arrows denote electron flow and dashed arrows indicate proposed pathways yet to be experimentally verified. AmoABCXYZ ammonia monooxygenase subunit A, B, C, X, Y, and Z, NirK copper-containing nitrite reductase, AccB acetyl-CoA carboxylase beta, Msr Malonic semialdehyde reductase, 4HBD 4-hydroxybutyryl-CoA dehydratase, CoxA Cbb3-type cytochrome c oxidase subunit I, PetB putative cytochrome b/b6 domain protein, Amt-1 and Amt-2 type 1 and 2 ammonium transporters, SSS-1 and SSS-2 type 1 and 2 sodium-solute symporters, Ut urea transporter, UreC urease subunit alpha, GlnB nitrogen regulatory protein P-II, PstAC phosphate uptake transporter permease protein A and C, PstB phosphate transporter ATP-binding protein, PiT low affinity inorganic phosphate transporter, AtpA ATP synthase subunit A, FlaB archaeal flagellin.

To evaluate competition between different AOA phylotypes, in situ gene expression profiles of the six major AOA lineages in our soils were compared to three AOA strains, for which RNA seq-based gene expression patterns in nutrient-replete, exponentially growing cultures were available [48, 51, 52], similar to the approach described previously for *N. viennensis* [48]. The log_2_-transformed *rpoB-*normalized RPKM values of key metabolism genes showed highly similar expression patterns in soils and pure culture strains (Fig. 5 and Supplementary Table S15). Active *Nitrosopumilales* in soil and *N. maritimus* SCM1 in pure culture showed less than 10-fold variation in *rpoB*-normalized expression for tested genes. Similarly, gene expression in *Nitrososphaerales* families in soil followed *N. viennensis* EN76 transcriptional patterns of several key genes of ammonia oxidation (*amoABCXYZ*, *nirK*), CO_2_ fixation (*4hbd*, *accB, msr*), respiration (*coxA*), and ATP synthesis (*atpA*).

The expression of some nutrient utilization related genes was different from that in pure culture and showed lineage-specific patterns in the soil. Most *Nitrososphaerales* families showed more than one order of magnitude higher expression of high-affinity ammonium transporter *amt*-1 (except NS-epsilon) in situ compared to *N. viennensis* in pure culture. Higher expression of *amt*-1 than *amt*-2 genes was observed in all AOA lineages containing both genes, consistent with previously observed transcriptional responses of *N. maritimus* at low ammonium concentrations, supporting the hypothesis of higher substrate affinity of Amt-1 than Amt-2 [53, 54], and suggesting that AOA in situ were largely ammonia-limited. NS-epsilon was the only AOA family with high *amt*-2 gene expression (equivalent to *amt*-1 expression level in other AOA lineages), whereas *amt*-1 appeared to be absent in this lineage. More importantly, *Nitrososphaerales* families, except NS-delta, expressed urease (*ureC*, 13–22-times higher) and urea transporter (*ut*, 41–177-time higher) orders of magnitude higher than ammonium-replete *N. viennensis* cultures, strongly suggesting in situ urea utilization in the ammonia-limited soils. Minimal urease expression, as well as absence of high-affinity urea transporter (Ut) and low affinity sodium-solute symporter (SSS-1) genes were found in NS-delta, which may explain their relatively low activity despite high cell abundance in our soils. *Nitrosopumilales* in our soils mostly lacked urea utilization genes and appeared to rely on free ammonium as nitrogen source and uptake by *amt*. Additionally, NS-beta-associated lineage, NS-delta, and NS-beta expressed ATP binding subunit of the high-affinity P transporter *pstB* orders of magnitude higher in soil than *N. viennensis* in pure culture, suggesting that growth of these lineages may have been limited by phosphate availability. Different land management and seasonality affected these gene expression patterns to some extent, but no drastic switch of metabolic pathways was suggested in any of these AOA lineages (Supplementary Table S15).

Six of the MAGs from this study contained *rpoB* genes, allowing for comparison of their associated gene expression with pure cultures and family-level aggregates (Supplementary Fig. S32, Supplementary Tables S16–S25). Some genes were differently expressed in individual MAGs than at the corresponding whole family-level aggregate. For example, type 1 and 2 sodium-solute symporters (SSS-1 and SSS-2) were on average minimally expressed within each family-level lineage (Fig. 5), but appeared to be highly expressed in some of our assembled MAGs (Supplementary Fig. S32). However, this discrepancy might be exacerbated by the low recruitment of metatranscriptomic reads mapped to single MAGs, and therefore may be a result of less reliable statistics.

We further examined the expression of the lineage-specific protein clusters (identified by comparative genomics as described above) that may reflect unique environmental adaptations. The NS-beta-associated MAG expressed cation/proton antiporter and MFS transporter (summing multiple gene copies) at a similar level as some HP/HB related genes (*4hbd*, *accB*). A NRAMP divalent cation transporter unique to this lineage (cluster-22662) was expressed at similar level as ammonia transporter. Transcripts of other characterized low pH adaptation genes were not or rarely detected in this lineage. For NS-delta MAGs, we did not detect transcripts of the aspartate ammonia-lyase cluster specific to this lineage.

## Discussion

*Nitrososphaeria*-affiliated AOA represent the globally predominant archaeal lineages in soil. Since their discovery in soil nearly two decades ago [1, 55], substantial efforts have been made to understand the physiological and ecological basis for their success [13, 14, 56–58]. The combined seasonal metagenomic, metatranscriptomic, and biogeochemical analysis in highly active soil presented here reveals that a single soil type simultaneously provides viable ecological niches for 12 different family-level *Nitrososphaeria* lineages. Detection of gene expression in nearly all identified AOA indicates that the vast majority of them was indeed viable and active in the soil and did not represent relic DNA or dead biomass [59].

Microbial activities generally followed the expected seasonal trends with higher soil respiration and nitrification potentials in the warmer summer months, than in the colder fall months (Supplementary Fig. S3). However, the higher activities and the approximately seven-degree higher average daily temperatures were likely not pronounced enough to become visible for overall increased gene abundances or enhanced gene expression activity. Notably, nitrate accumulation mirrored nitrification potentials only during fall, whereas increasing soil moisture content at the onset of the rainy season in May and June was associated with strong loss of nitrate in all plots, suggesting either linked nitrification-denitrification, or significant nitrate leaching during the rainy season.

Phylotypes of predominant *Nitrososphaeria* lineages composed a patchwork of different combinations of nitrogen and phosphorous acquisition strategies. Activity of 12 family-level lineages, including all thus far cultivated mesophilic AOA families with high in situ transcriptional activities, indicates coexisting niches for AOA with high- or low-affinity nutrient uptake and ammonia oxidization systems in soil (Fig. 5). These results thus indicate that in addition to kinetic properties [19, 22], the patchwork of specialized nutrient acquisition strategies among diverse AOA lineages may provide an important mechanism governing competition and coexistence among AOA, as well as between AOA, AOB, and comammox ammonia oxidizers. These results suggest that the diverse *Nitrososphaeria* assemblage represents a reservoir of coexisting specialist phylotypes that exploit small-scale spatial and temporal heterogeneity of soil physical and chemical properties, temperature, and moisture patterns, and outcompete low diversity generalist assemblages of AOB and comammox bacteria under a variety of natural environmental conditions. This conclusion is consistent with the observation in bacterial communities that ecological specialists can have an advantage over generalists at the local scale [60, 61].

Several studies have indicated urea as an important substrate of AOA in marine environments [27–29] and urease-positive AOA have also been found in various soils [24, 45, 62, 63]. However, evidence for in situ urea utilization of soil AOA is lacking. The high proportion of AOA that encode and highly express urease and urea transporters (Fig. 5) strongly suggest that both ammonia and urea are important substrates and are directly metabolized by a wide range of *Nitrososphaeria* AOA in soil. However, proportions of ammonia and urea oxidation rates by AOA cannot be directly deduced from the metatranscriptomic data, and direct activity measurements in soil are hampered by the widespread capacity for urea hydrolysis among heterotrophic soil microbes [64, 65]. This observation is consistent with the notion that soil AOA activity is often tightly linked to organic matter mineralization [66–68]. We found no evidence for utilization of cyanate, another potential nitrogen source for archaeal nitrification recently shown to be actively cycled in soil [28, 69]. Three AOA lineages, the NS-delta, NS-beta, and NS-beta-associated, were highly expressing ATP binding subunit of the high-affinity P transporter (PstB) in the soil, suggesting their adaptation to low phosphorus availability. Indeed, available phosphorus was correlated with the variation of soil AOA composition (Supplementary Fig. S1), and these three AOA lineages co-dominated the expression of *pstB* in plot 1 where available phosphorus concentration was the lowest year around (Supplementary Fig. S22). Phosphorus availability was previously identified as one of the main selective forces determining the biogeography of different marine AOA genotypes [70]. Our results indicate that phosphorous availability very likely affects the distribution and ecophysiology of AOA in soil as well. Further research will be needed to understand how ammonia, urea, and phosphorus metabolism are regulated among different AOA lineages.

Previous studies have suggested that the globally predominant soil archaeal lineages, e.g. the NS-delta lineage [55, 71], may not act as canonical autotrophic ammonia oxidizers, but instead oxidize other substrates or grow heterotrophically [72, 73]. Although the fraction of active cells could not be determined directly, archaeal *amoA* was among the most highly transcribed genes in our metatranscriptomes, suggesting that a large fraction of *Nitrososphaeria* cells was indeed metabolically active and engaging in ammonia oxidation. NS-delta AOA were proportionally less transcriptionally active than other lineages (Fig. 4, Supplementary Figs. S30 and S31). However, *rpoB*-normalized gene expression ratios suggested that among active NS-delta cells, *amo* and *nirK*, along with respiratory complexes, A-type ATPase, and CO_2_ fixation pathway were similarly expressed as in other lineages (Fig. 5). Additionally, *amo* was always among the highest transcribed genes in each of the NS-delta MAGs in our soil (among top six highest expressed genes, Supplementary Tables S22–S24), similar to MAGs of other AOA lineages in our soils and in pure cultures (Supplementary Fig. S32). It is thus less likely that ammonia oxidation is just an auxiliary metabolism for the active NS-delta cells. Together, these data suggest that, although a larger fraction of NS-delta cells might have been dead or inactive than in other lineages, the active fraction of NS-delta cells engaged in ammonia oxidation and CO_2_ fixation. However, we cannot rule out the possibility that in active NS-delta the general transcriptional level is intrinsically lower compared to other AOA lineages, leading to the lower recovery of affiliated reads from metatranscriptomic data. Although the apparent absence of recognizable high affinity urea transporters (Ut) and putative low affinity urea uptake systems (SSS-1) in the NS-delta lineage remains somewhat puzzling, it suggests that NS-delta is either specialized in ammonium utilization, has lost direct urea uptake and utilization capacity, or may convert other organic N sources into urea.

Most *Nitrosopumilales* AOA lacked the genetic capability to use urea, although a low number of transcripts of low affinity urea uptake system (SSS-1) and urease were found within this group, suggesting that some low abundance phylotypes may utilize urea. *Nitrosopumilales* in our soils exhibited the highest *rpoB*-normalized transcript ratios for Amo, high-affinity Amt (Amt-1), NirK, and CO_2_ fixation pathway enzymes, indicating a similarly oligotrophic, primarily ammonia-dependent autotrophic life style of soil *Nitrosopumilales*, as observed in their marine counterparts [17, 29, 51, 74]. However, besides the low prevalence of urea utilization genes, we found no obvious explanation for their relatively low overall abundance in the unfertilized and apparently nitrogen-limited soils [71, 73, 75–77].

In summary, the presented metagenomes and transcriptomes provide a detailed and comprehensive view into the gene inventory and expression patterns of the globally predominant lineages of archaea in soil. Our results identify gene inventory and transcriptional patterns of several uncultivated *Nitrososphaerales* families dominant in soils: (i) NS-delta was always one of the numerally dominant AOA lineages in different soils, but showed disproportionally low transcriptional activity compared to other families, did not possess urea transporter genes, and showed minimal expression of urease genes; (ii) NS-epsilon represented one of the most transcriptionally active families in soils. It contained only a low-affinity ammonium transporter (Amt-2) and was highly expressing it, while in other families Amt-1 was most highly expressed in the soils. Additionally, NS-epsilon MAGs contained both V-type and A-type copies of ATPase genes, suggesting a wide environmental adaptability; (iii) NS-beta-associated AOA and a representative MAG were found exclusively in our unmanaged soil, and likely represent a new low pH (<6)-adapted family. Like other families, it possessed *nirK* genes, but expressed those at much lower levels that within the neutrophilic families. It also contained two copies of both *amt-1* and *amt-2* genes in one single MAG.

### Supplementary information

Supplemental Materials

Supplemental Table S1

Supplemental Table S2

Supplemental Table S3

Supplemental Table S4

Supplemental Table S6

Supplemental Table S7

Supplemental Table S8

Supplemental Table S9

Supplemental Table S10

Supplemental Table S11

Supplemental Table S12

Supplemental Table S13

Supplemental Table S14

Supplemental Table S15

Supplemental Table S16-25

## Data Availability

The raw 16S rRNA gene amplicon sequencing data were deposited to NCBI under BioProject accession number PRJNA831877. Metagenomic and metatranscriptomic shotgun sequences are deposited at NCBI under SRA study IDs SRP258623, SRP258632, SRP258633, SRP258644, SRP258647, SRP258661, SRP258662, SRP258690, SRP258692, SRP258713, SRP258714, SRP258717, SRP258719, SRP258720, SRP258805, SRP258806, SRP258807, SRP258809, SRP258810, SRP258816, SRP258818, SRP258823, SRP258824, SRP258830, SRP258834, SRP258836, SRP258837, SRP258838, SRP258846, SRP270154, SRP282650, SRP282652, SRP282654, SRP282655, and SRP282656. All read assemblies (contigs, scaffolds and bins) are available in the IMG genome database (https://img.jgi.doe.gov/) or at NCBI BioProject PRJNA902846. See Supplementary Table S7 for details.
