# Supplementary material for: Nitrogen and phosphorous acquisition strategies drive coexistence patterns among archaeal lineages in soil
Source: ISME J. 2023 Aug 18;17(11):1839–50. doi: 10.1038/s41396-023-01493-y (PMC10579303; doi:10.1038/s41396-023-01493-y)
Supplement: Supplementary file 1 — Supplemental Materials [file 41396_2023_1493_MOESM1_ESM.pdf]

Nitrogen and phosphorous acquisition strategies drive coexistence patterns among archaeal lineages in soil

Jun Zhao[1], Laibin Huang[1], Seemanti Chakrabarti[1], Jennifer Cooper[2], EunKyung Choi[1], Carolina Ganan[1], Bryn Tolchinsky[1], Eric W. Triplett[3], Samira H. Daroub[2], Willm Martens-Habbena[1*]

[1] Fort Lauderdale Research and Education Center, Department of Microbiology and Cell Science, University of Florida, Davie, FL, 33314, USA

[2] Everglades Research and Education Center, Soil and Water Sciences Department, University of Florida, Belle Glade, 33430, USA

[3] Department of Microbiology and Cell Science, University of Florida, Gainesville, FL, 32611, USA

* Correspondence to:

Willm Martens-Habbena (email: w.martenshabbena@ufl.edu)

Supplementary Materials

Supplementary Methods, Results, Discussion, and References **Page S2-S10**

Supplementary Table S5 **Page S11**

Supplementary Figures S1-S32 **Page S12-S66**

Supplementary Methods

Site description and soil collection

Soils were collected from five differently managed soil plots in the Everglades Agricultural Area (EAA) in South Florida. The plots were located in close proximity to each other to ensure similarity in weather patterns. Plot 1 was unmanaged and covered with natural vegetation. Plot 2 was cultivated with a rotation of sugarcane, spinach, fallow treatment and sugarcane. Plot 3 was cultivated with a rotation of sugarcane, sweetcorn, rice, and sugarcane. Plot 4 was cultivated with a rotation of sugarcane, sweetcorn, fallow treatment, and sugarcane. Lastly, plot 5 was cultivated with sugarcane throughout the year. Plots 2–5 have been under long-term agricultural management with a history of liming to adjust soil pH to near-neutral. Samples were collected every 6–8 weeks between March 2017 and April 2018. From each plot, biological triplicates (50–200 m apart) were sampled and each replicate contained nine 10-cm topsoil cores collected randomly within a subplot of 10×10 m^2. All samples were homogenized in the field. Sub-samples for DNA and RNA extraction were transferred to Ziploc bags and immediately frozen between sheets of dry ice and subsequently stored at -80ºC for further processing. Samples for nutrient analyses were transferred into cooled 2 M KCl solution, and remaining soil for other analyses was stored on ice until further analysis.

Soil physiochemical measurements

Soil pH was determined using a soil-to-water ratio of 1:2 (w/w). Soil NH_4^+, NO_2^-, NO_3^- and urea concentrations were determined colorimetrically after extraction with 2 M KCl solution dissolved in phosphate buffer as previously described (Mulvenna and Savidge, 1992; Huang et al., 2021). Total P and available P were measured using a continuous segmented flow analyzer (Auto Analyzer 3, Seal Analytical Inc., Mequon, WI, USA) according to EPA method 365.1. Samples for available P were filtered through 0.45 μm filters (EMD Millepore Corp., Darmstadt, Germany), and samples for total P were digested by the ammonium-persulfate method prior to analyses. Soil organic matter (OM) content was measured by loss on ignition and dissolved organic carbon (DOC) content was measured using a TOC analyzer (Shimadzu, Kyoto, Japan). Soil CO_2, N_2O and CH_4 fluxes were measured in the field using a Gasmet (DX4040, Helsinki, Finland) fitted with a survey chamber (Li-cor LI-8100, Lincoln, Nebraska) as previously described (Storlien et al., 2014).

Soil potential nitrification rates were measured using slurry method as described previously (Huang et al., 2021). Briefly, two to four days after returning from the field 1.0-g samples of field-moist soil were mixed with 20 ml SFCM media in 50-ml Falcon tubes. The SFCM media contained 1 g L^{-1} NaCl, 0.4 g L^{-1} $MgCl_2$·6 H_2O, 0.1 g L^{-1} $CaCl_2$·2H_2O and 0.5 g L^{-1} KCl in double deionized water (EMD Millipore Synergy UV). After autoclaving the media was supplemented sterile stock solutions as follows (per liter): 2 mL $NaHCO_3$ (84 g L^{-1}), 5 mL KH_2PO_4 (0.4 g L^{-1}), 1ml FeNaEDTA (2.75 g L^{-1}), 1 ml modified non-chelated trace element solution (Martens-Habbena et al., 2009). The medium pH was 7.2 and 1 mM NH_4Cl was added as nitrogen substrate. Samples were vortexed briefly and incubated at 28 ºC. Samples for determination of NO_2^- + NO_3^- (1.0 mL) were collected at 0 h, 24 h and 48 h of incubation. Nutrient concentrations were determined using colorimetric assays as described above and potential rates were determined from linear regression of NO_2^- + NO_3^- accumulation over the first 48 h of incubation.

DNA and RNA extraction

Soil samples (5.0 g) were homogenized under liquid nitrogen using DNA-free mortar and pestle, and DNA was subsequently extracted from 0.25 g of sample using the DNeasy PowerSoil Kit (Qiagen, Hilden, Germany) following manufacturer's instructions, except that bead-beating was performed twice at 4.5 m s^{-1} for 15 s using a FastPrep-24 (MP Biomedicals, Santa Ana, CA, USA). DNA was dissolved in 50 µl molecular biology grade water and stored at -20 °C before use. Soil RNA was also extracted from homogenized 2 g samples using RNeasy PowerSoil Total RNA Kit (Qiagen) following kit instructions. DNA was removed by treating samples twice with DNase using the TURBO DNA-free Kit (Thermo Fisher Scientific). Absence of DNA was confirmed by negative endpoint PCR of 16S rRNA genes.

Amplicon sequencing of 16S rRNA genes

The 16S rRNA gene fragment (V4-V5 region) was amplified using primer pair 515F and 926R (Quince et al., 2011; Parada et al., 2016) with specific adaptors. PCR amplifications, library preparations and DNA sequencing were conducted at the Environmental Sample Preparation and Sequencing Facility (ESPSF) at Argonne National Laboratory following the Earth Microbiome protocol (Thompson et al., 2017). QIIME2 (version 2020.2) was used for sequence analyses (Caporaso et al., 2010), including quality filtering, denoising, paired-end read merging and dereplication using DADA2 to generate amplicon sequence variants (ASVs) (Callahan et al., 2016). ASVs were taxonomically classified against SILVA database release 138.1 using the scikit-learn classifier (Pedregosa et al., 2011) embedded in QIIME2. ASVs that were unclassified at domain level or classified as eukaryotes were removed from statistical analyses.

Shotgun sequencing, quality filtering, sequence assembly and genome binning

Shotgun metagenomic and metatranscriptomic raw sequences were filtered using BBTools v38.75 (http://bbtools.jgi.doe.gov). Paired-end reads were processed with BBDuk to remove contaminants and adapter sequence. BBDuk was further used to remove reads that contained 4 or more 'N' bases, with an average quality < 3 or with a minimum length ≤51 bp or 33% of the full read length. BBMap was used to map and remove human and animal sequences and common microbial contaminants.

Genome assembly and binning was performed on each individual filtered metagenomic dataset, as well as on combined metagenomic datasets, including all metagenomes from the agricultural plots 3 and 5, or unmanaged plot 1, respectively, using the JGI standard workflow (Clum et al., 2021). Filtered high quality reads were further corrected by bbcms (BBTools software package) using Tadpole error-correction algorithm (mincount=2 highcountfraction=0.6). The corrected reads were assembled by metaSPAdes v3.13.0 (parameters: -m 2000 -ospades3 --only-assembler -k 33,55,77,99,127 --meta) (Nurk et al., 2017). The input short read set was mapped to the final assembly and coverage information generated with BBMap. MetaBAT v2:2.15 (Kang et al., 2015) with default parameters was used to generate genome bins based on the coverage and tetranucleotide frequency of the assembly. Alternatively, each metagenome dataset was randomly subset to 20% of the original filtered read count, and was assembled by megahit v1.1.4 (Li et al., 2015) with default parameters, followed by genome binning using MaxBin 2 (Wu et al., 2016) implemented in MetaWRAP v1.2 (Uritskiy et al., 2018). All bins obtained from above pipelines were taxonomically classified by GTDB-tk v1.3.0 (Chaumeil et al., 2020) and their quality was assessed using CheckM v1.1.03 (Parks et al., 2015). Genome bins assigned to class *Nitrososphaeria* were selected and dereplicated using dRep v2.3.2 (Olm et al., 2017) after removing MAGs with <70% completeness and >10% contamination. This resulted in a total of ten

AOA MAGs. Prediction and annotation of protein-coding genes was performed by Prokka (Seemann, 2014) using arCOGs (Makarova et al., 2015), Uniref50 (Suzek et al., 2007), and NCBI RefSeq (Pruitt et al., 2007) databases. For comparative genomics, 92 genomes and MAGs (including ten MAGs from this study and 82 from NCBI or IMG, Table S4) were collected and their protein dataset was clustered into ortholog protein groups in all selected genomes based on sequence identity of 35% using Roary v3.12.0 (Page et al., 2015). Average nucleotide identity (ANIs) between these AOA MAGs and reference genomes were calculated using fastANI v1.1 (Jain et al., 2018).

Short read taxonomic classification and mapping

The filtered shotgun short reads were paired-end merged by BBMerge with least 10 overlapping bases and only reads ≥150 bp length were retained for further analysis. The resultant merged reads were used as query for Diamond v0.9.34 (Buchfink et al., 2015) search against NCBI RefSeq protein database (updated on 2020-06-02) for all short read taxonomy and function annotations (e-value 10^{-5}, 50% amino acid identity and 60% query coverage). Gene and transcript abundances were normalized as reads per kilobase per million reads mapped (RPKM).

Statistical analyses

Two-way ANOVA was employed to determine the effect of land use and seasonal change on the abundance and activity of different nitrifier groups, followed by a Tukey *post-hoc* test to determine significant mean differences. Non-metric multidimensional scaling (NMDS) based on Bray-Curtis dissimilarity matrix of AOA community as well as total prokaryotic community composition based on 16S rRNA genes was performed using the vegan package (version 2.5-7) in *R* (version 4.1.1) and analysis of similarity (ANOSIM) was used to assess the compositional difference between different land uses (Philip, 2003). Function "envfit" was used to test the significance of geochemical factors (as vectors) and land management (as factors) for the NMDS ordinations, with number of permutations 999 by vegan(Philip, 2003). Differences at $p < 0.05$ were considered statistically significant.

Supplemental Results and Discussion

Soil biogeochemical properties and microbial activities

Soil physico-chemical properties, organic matter mineralization, and nitrification potential were monitored in five soil plots of varying management over a 12-month time series from May 2017 to April 2018 (see details of soil plots and management in Table S1 and Ref (Huang et al., 2021)). Temperature and precipitation were typical for the tropical climate in South Florida, with maximum daily average 10-cm soil temperatures of 28 to 30 ºC and high, but variable precipitation (7.5–230 L m^{-2} $week^{-1}$) between May and September, followed by cooler temperatures of 18 to 23 ºC and low precipitation (< 2 L m^{-2} $week^{-1}$) between November and April (Fig. S3a and Table S2). All five plots showed a strong seasonal cycle with higher ammonia and nitrate concentrations during the dry fall and winter seasons, and lower ammonium and nitrate concentrations during the wet summers (Fig. S3b, c and Table S6). Urea was lower than or at the same concentration as ammonium in all seasons (Fig. S3d). Soil total phosphorus (TP) was averagely lower in plot 1 and 3 (Fig. S3e), while available phosphorus (AP) was consistently the lowest in the unmanaged soil plot 1 (Fig. S3f). At both sampling points of shotgun sequencing, AP was highest in plot 5, followed by plot 3 and plot 1. Soil microbial respiration assessed via *in situ* soil surface-atmosphere

CO_2 fluxes were highly variable with plot averages between 56.94 ± 28.30 (plot 2 in March) and 446.42 ± 121.56 mg C m^2 h^{-1} (plot 5 September), but fluxes tended to be higher in summer than in winter (Fig. S3g and Table S6). No seasonal CO_2 flux peak was detected in plot 3, which was flooded for rice cultivation during peak summer month. Potential nitrification rates were lower in plant-covered plots 1 and 5, compared to the partially fallow or flooded plots 2, 3, and 4 (Fig. S3h). In line with seasonal profiles of temperature, soil moisture, and CO_2 fluxes, highest potential nitrification rates between 13.0 (plot 1) to 36.9 (plot 3) μg NO_x^--N g^{-1} dry soil d^{-1} were observed in June. Although soil-atmosphere CO_2 fluxes and potential nitrification rates were only determined monthly, the data are consistent with a typical tropical seasonal cycle with higher microbial activities in the warm and wet summer season and lower activities during cooler and drier fall and winter season.

Spatial and temporal patterns of overall microbial community and AOA community composition

Nonmetric multidimensional scaling (NMDS) of Bray-Curtis dissimilarity distances revealed clear separation of AOA community composition between different plots throughout the year (ANOSIM, $P \leq 0.001$) (Fig. S1), whereas seasonality had no significant influence on soil AOA community composition ($P = 0.191$). Overall prokaryotic microbial community richness (number of ASVs) and evenness (Shannon's index) was lower in the unmanaged plot 1 than in the agricultural plots 2–4 (Fig. S2a, b). Similar patterns of community composition distinctly separated by different land management ($P \leq 0.001$) were also observed in total prokaryotic microbial communities (Figs. S1a, b and 2c), corroborating previous observations (Orellana et al., 2018; Huang et al., 2021).

Key metabolic proteins in soil *Nitrososphaeria* with multiple copies or history of horizontal transfer

Some proteins within the key metabolism protein inventory do not follow order-level radiative diversification, likely due to horizontal gene transfer or presence of multiple homolog genes in individual genomes, e.g. in Amt transporters and energy-transducing A- and V-type ATPases (Wang et al., 2019). Consequently, clustering of Amt-1, Amt-2, AtpA, AmoC, NirK, UreC, and GlnB proteins did not always follow AmoA phylogeny. For Amt-1 phylogeny, family NS-gamma and order *Ca.* Nitrosotaleales formed a monophyletic group, whereas in the Amt-2 phylogeny family NS-delta, orders of *Ca.* Nitrosotaleales and *Nitrosopumilales* clustered closely together (Fig. S16).

In the UreC phylogeny NS-delta also clustered among *Ca.* Nitrosotaleales and *Nitrosopumilales* groups, while the remaining *Nitrososphaerales* families formed a distinct monophyletic group (Fig. S19). Interestingly, some *Nitrososphaeraceae* genomes, e.g., *Nitrososphaera viennensis* EN76 and *Ca.* Nitrososphaera everglades SR1 contain two *ureC* gene copies affiliated with different monophyletic lineages.

The AtpA phylogeny united lineages of putative acidophilic and piezophilic AOA comprising *Ca.* Nitrosotaleaceae, NS-gamma and NS-zeta (V-type group), and combined *Nitrosopumilales* and the other *Nitrososphaerales* families into a distinct group (A-type group) (Wang et al., 2019) (Fig. S25). Notably, two NS-epsilon reference genomes contained both V- and A-type copies of ATPase genes, suggesting a wide environmental adaptability of these strains. Other proteins, i.e., AmoC, NirK and GlnB are present in multiple distantly related copies in AOA genomes, similarly leading to low congruence with AmoA-based phylogeny (Figs. S6, S10, S20).

Phylogeny and activity of *Nitrospira* and AOB

Despite much lower abundance and transcriptional activity than AOA, the relative abundance and/or activity of AOB and comammox increased following conversion of the land to cropping system (Figs. 1C, 3C). It has been suggested that AOB favour high concentrations of inorganic ammonium supply (Di et al., 2009; Jia and Conrad, 2009; Verhamme et al., 2011; Trivedi et al., 2019; Zhao et al., 2020a; Sun et al., 2022) and are more active under neutral than acidic soil pH conditions (Nicol et al., 2008; Zhang et al., 2012). Both factors might affect competitive status of AOB, but the higher proportion and activity of AOB in the cropping fields than in the unmanaged soil in our study is more likely the consequence of increased pH by liming during the land conversion, since the chemical N fertilizer was not applied before soil sampling. This will support previous conclusion that AOA population had higher ecosystem stability than AOB in response to a pH perturbation (Zhao et al., 2020b). Increase in soil pH could also have favoured comammox competition and activity following land conversion, as currently known comammox strains all grow optimally at neutral to slight alkaline pH conditions at 7.0-7.8 (Daims et al., 2015; van Kessel et al., 2015).

Metagenomic analysis estimated much higher AOA than *Nitrospira* abundances in our soils (Fig. 1B, C), but the transcriptional activity of AOA (estimated by *amoA*) and *Nitrospira* (estimated by *nxrB*) were at the similar level (Fig. 3A, D). Nitrate but not nitrite accumulation was observed in the fields all year around (Table S6). These results collectively showed balanced activities of ammonia and nitrite oxidizers in soils to complete nitrification. The much higher abundance of AOA than NOB suggested lower average cell specific rate of the former than the latter nitrifying groups in transforming N in the soils, which was consistent with the known physiological dynamics (specific cell rates) of representative AOA and NOB strains (Pester et al., 2014; Stieglmeier et al., 2014; Nowka et al., 2015; Lehtovirta-Morley et al., 2016; Zhang et al., 2020).

Opposite to AOA, soil *Nitrospira* abundance and activity were co-dominated by generalist (well adapting wide range of habitats) rather than specialist phylotypes (competitive in special habitats) in the soils. The major *Nitrospira* taxa in soils were mostly the same in both unmanaged and agricultural soils (e.g., Ga0376028_0077477_766_2055 and Ga0401874_001960_645_1835), showing high function stability (tolerance) to environmental disturbance by land-use conversion, including soil pH change. Only one phylotype (Ga0376026_0398247_284_1573) showed drastic decrease in relative abundance and activity following agricultural management, representing the main specialist *Nitrospira* in the unmanaged, low pH soil.

Reference

U.S. Environmental Protection Agency, 1993. EPA 365.1: Determination of phosphorus by semi-automated colorimetry, Revision 2.0

Buchfink, B., Xie, C., Huson, D.H., 2015. Fast and sensitive protein alignment using DIAMOND. Nature Methods 12, 59-60.

Callahan, B.J., McMurdie, P.J., Rosen, M.J., Han, A.W., Johnson, A.J., Holmes, S.P., 2016. DADA2: High-resolution sample inference from Illumina amplicon data. Nature Methods 13, 581-583.

Caporaso, J.G., Fierer, N., Peña, A.G., Goodrich, J.K., Gordon, J.I., Huttley, G.A., Kelley, S.T., Knights, D., Mcdonald, D., Muegge, B.D., Pirrung, M., Reeder, J., Widmann, J.,

Yatsunenko, T., Zaneveld, J., 2010. QIIME allows analysis of high-throughput community sequencing data. Nature Methods 7, 335-336.
Chaumeil, P.A., Mussig, A.J., Hugenholtz, P., Parks, D.H., 2020. GTDB-Tk: a toolkit to classify genomes with the Genome Taxonomy Database. Bioinformatics 36, 1925–1927.
Clum, A., Huntemann, M., Bushnell, B., Foster, B., Foster, B., Roux, S., Hajek Patrick, P., Varghese, N., Mukherjee, S., Reddy, T.B.K., Daum, C., Yoshinaga, Y., O'Malley, R., Seshadri, R., Kyrpides Nikos, C., Eloe-Fadrosh Emiley, A., Chen, I.M.A., Copeland, A., Ivanova Natalia, N., 2021. DOE JGI Metagenome Workflow. mSystems 6, e00804-00820.
Daims, H., Lebedeva, E.V., Pjevac, P., Han, P., Herbold, C., Albertsen, M., Jehmlich, N., Palatinszky, M., Vierheilig, J., Bulaev, A., Kirkegaard, R.H., von Bergen, M., Rattei, T., Bendinger, B., Nielsen, P.H., Wagner, M., 2015. Complete nitrification by Nitrospira bacteria. Nature 528, 504-509.
Di, H.J., Cameron, K.C., Shen, J.P., Winefield, C.S., O'Callaghan, M., Bowatte, S., He, J.Z., 2009. Nitrification driven by bacteria and not archaea in nitrogen-rich grassland soils. Nature Geoscience 2, 621-624.
Huang, L., Chakrabarti, S., Cooper, J., Perez, A., John, S.M., Daroub, S.H., Martens-Habbena, W., 2021. Ammonia-oxidizing archaea are integral to nitrogen cycling in a highly fertile agricultural soil. ISME Communications 1, 19.
Jain, C., Rodriguez, R.L., Phillippy, A.M., Konstantinidis, K.T., Aluru, S., 2018. High throughput ANI analysis of 90K prokaryotic genomes reveals clear species boundaries. Nature Communications 9, 5114.
Jia, Z., Conrad, R., 2009. Bacteria rather than Archaea dominate microbial ammonia oxidation in an agricultural soil. Environmental Microbiology 11, 1658-1671.
Kang, D.D., Froula, J., Egan, R., Wang, Z., 2015. MetaBAT, an efficient tool for accurately reconstructing single genomes from complex microbial communities. PeerJ 3, e1165.
Lehtovirta-Morley, L.E., Ross, J., Hink, L., Weber, E.B., Gubry-Rangin, C., Thion, C., Prosser, J.I., Nicol, G.W., 2016. Isolation of '*Candidatus* Nitrosocosmicus franklandus', a novel ureolytic soil archaeal ammonia oxidiser with tolerance to high ammonia concentration. FEMS Microbiology Ecology 92, fiw057.
Li, D., Liu, C.M., Luo, R., Sadakane, K., Lam, T.W., 2015. MEGAHIT: an ultra-fast single-node solution for large and complex metagenomics assembly via succinct de Bruijn graph. Bioinformatics 31, 1674-1676.
Makarova, K.S., Wolf, Y.I., Koonin, E.V., 2015. Archaeal Clusters of Orthologous Genes (arCOGs): An Update and Application for Analysis of Shared Features between Thermococcales, Methanococcales, and Methanobacteriales. Life (Basel) 5, 818-840.
Martens-Habbena, W., Berube, P.M., Urakawa, H., de la Torre, J.R., Stahl, D.A., 2009. Ammonia oxidation kinetics determine niche separation of nitrifying Archaea and Bacteria. Nature 461, 976-979.
Mulvenna, P.F., Savidge, G., 1992. A modified manual method for the determination of urea in seawater using diacetylmonoxime reagent. Estuarine, Coastal and Shelf Science 34, 429-438.
Nicol, G.W., Leininger, S., Schleper, C., Prosser, J.I., 2008. The influence of soil pH on the diversity, abundance and transcriptional activity of ammonia oxidizing archaea and bacteria. Environmental Microbiology 10, 2966-2978.

Nowka, B., Daims, H., Spieck, E., 2015. Comparison of oxidation kinetics of nitrite-oxidizing bacteria: nitrite availability as a key factor in niche differentiation. Applied and Environmental Microbiology 81, 745-753.

Nurk, S., Meleshko, D., Korobeynikov, A., Pevzner, P.A., 2017. metaSPAdes: a new versatile metagenomic assembler. Genome Research 27, 824-834.

Olm, M.R., Brown, C.T., Brooks, B., Banfield, J.F., 2017. dRep: a tool for fast and accurate genomic comparisons that enables improved genome recovery from metagenomes through de-replication. The ISME Journal 11, 2864-2868.

Orellana, L.H., Chee-Sanford, J.C., Sanford, R.A., Loffler, F.E., Konstantinidis, K.T., 2018. Year-round shotgun metagenomes reveal stable microbial communities in agricultural soils and novel ammonia oxidizers responding to fertilization. Applied and Environmental Microbiology 84, 14.

Page, A.J., Cummins, C.A., Hunt, M., Wong, V.K., Reuter, S., Holden, M.T., Fookes, M., Falush, D., Keane, J.A., Parkhill, J., 2015. Roary: rapid large-scale prokaryote pan genome analysis. Bioinformatics 31, 3691-3693.

Parada, A.E., Needham, D.M., Fuhrman, J.A., 2016. Every base matters: assessing small subunit rRNA primers for marine microbiomes with mock communities, time series and global field samples. Environmental Microbiology 18, 1403-1414.

Parks, D.H., Imelfort, M., Skennerton, C.T., Hugenholtz, P., Tyson, G.W., 2015. CheckM: assessing the quality of microbial genomes recovered from isolates, single cells, and metagenomes. Genome Research 25, 1043-1055.

Pedregosa, F., Varoquaux, G., Gramfort, A., Michel, V., Thirion, B., Grisel, O., Blondel, M., Prettenhofer, P., Weiss, R., Dubourg, V., Vanderplas, J., Passos, A., Cournapeau, D., Brucher, M., Perrot, M., Duchesnay, E., 2011. Scikit-learn: Machine Learning in Python. Journal of Machine Learning Research 12, 2825-2830.

Pester, M., Maixner, F., Berry, D., Rattei, T., Koch, H., Lucker, S., Nowka, B., Richter, A., Spieck, E., Lebedeva, E., Loy, A., Wagner, M., Daims, H., 2014. NxrB encoding the beta subunit of nitrite oxidoreductase as functional and phylogenetic marker for nitrite-oxidizing *Nitrospira*. Environmental Microbiology 16, 3055-3071.

Philip, D., 2003. VEGAN, a package of R functions for community ecology. Journal of Vegetation Science 14, 927-930.

Pruitt, K.D., Tatusova, T., Maglott, D.R., 2007. NCBI reference sequences (RefSeq): a curated non-redundant sequence database of genomes, transcripts and proteins. Nucleic Acids Research 35, D61-65.

Quince, C., Lanzen, A., Davenport, R.J., Turnbaugh, P.J., 2011. Removing Noise From Pyrosequenced Amplicons. Bmc Bioinformatics 12, 18.

Seemann, T., 2014. Prokka: rapid prokaryotic genome annotation. Bioinformatics 30, 2068-2069.

Stieglmeier, M., Mooshammer, M., Kitzler, B., Wanek, W., Zechmeister-Boltenstern, S., Richter, A., Schleper, C., 2014. Aerobic nitrous oxide production through N-nitrosating hybrid formation in ammonia-oxidizing archaea. The ISME Journal 8, 1135-1146.

Storlien, J.O., Hons, F.M., Wight, J.P., Heilman, J.L., 2014. Carbon dioxide and nitrous oxide emissions impacted by bioenergy sorghum management. Soil Science Society of America Journal 78, 1694-1706.

Sun, X., Zhao, J., Bei, Q., Xia, W., Zhou, X., Zhao, B., Zhang, J., Jia, Z., 2022. Niche specialization and ecophysiological adaptation strategies of salt-tolerant nitrite oxidizers in soil. Biology and Fertility of Soils 58, 815-825.

Suzek, B.E., Huang, H., McGarvey, P., Mazumder, R., Wu, C.H., 2007. UniRef: comprehensive and non-redundant UniProt reference clusters. Bioinformatics 23, 1282-1288.

Thompson, L.R., Sanders, J.G., McDonald, D., Amir, A., Ladau, J., Locey, K.J., Prill, R.J., Tripathi, A., Gibbons, S.M., Ackermann, G., Navas-Molina, J.A., Janssen, S., Kopylova, E., Vazquez-Baeza, Y., Gonzalez, A., Morton, J.T., Mirarab, S., Xu, Z.Z., Jiang, L.J., Haroon, M.F., Kanbar, J., Zhu, Q.J., Song, S.J., Kosciolek, T., Bokulich, N.A., Lefler, J., Brislawn, C.J., Humphrey, G., Owens, S.M., Hampton-Marcell, J., Berg-Lyons, D., McKenzie, V., Fierer, N., Fuhrman, J.A., Clauset, A., Stevens, R.L., Shade, A., Pollard, K.S., Goodwin, K.D., Jansson, J.K., Gilbert, J.A., Knight, R., Rivera, J.L.A., Al-Moosawi, L., Alverdy, J., Amato, K.R., Andras, J., Angenent, L.T., Antonopoulos, D.A., Apprill, A., Armitage, D., Ballantine, K., Barta, J., Baum, J.K., Berry, A., Bhatnagar, A., Bhatnagar, M., Biddle, J.F., Bittner, L., Boldgiv, B., Bottos, E., Boyer, D.M., Braun, J., Brazelton, W., Brearley, F.Q., Campbell, A.H., Caporaso, J.G., Cardona, C., Carroll, J., Cary, S.C., Casper, B.B., Charles, T.C., Chu, H.Y., Claar, D.C., Clark, R.G., Clayton, J.B., Clemente, J.C., Cochran, A., Coleman, M.L., Collins, G., Colwell, R.R., Contreras, M., Crary, B.B., Creer, S., Cristol, D.A., Crump, B.C., Cui, D.Y., Daly, S.E., Davalos, L., Dawson, R.D., Defazio, J., Delsuc, F., Dionisi, H.M., Dominguez-Bello, M.G., Dowell, R., Dubinsky, E.A., Dunn, P.O., Ercolini, D., Espinoza, R.E., Ezenwa, V., Fenner, N., Findlay, H.S., Fleming, I.D., Vincenzo, F., Forsman, A., Freeman, C., Friedman, E.S., Galindo, G., Garcia, L., Garcia-Amado, M.A., Garshelis, D., Gasser, R.B., Gerdts, G., Gibson, M.K., Gifford, I., Gill, R.T., Giray, T., Gittel, A., Golyshin, P., Gong, D.L., Grossart, H.P., Guyton, K., Haig, S.J., Hale, V., Hall, R.S., Hallam, S.J., Handley, K.M., Hasan, N.A., Haydon, S.R., Hickman, J.E., Hidalgo, G., Hofmockel, K.S., Hooker, J., Hulth, S., Hultman, J., Hyde, E., Ibanez-Alamo, J.D., Jastrow, J.D., Jex, A.R., Johnson, L.S., Johnston, E.R., Joseph, S., Jurburg, S.D., Jurelevicius, D., Karlsson, A., Karlsson, R., Kauppinen, S., Kellogg, C.T.E., Kennedy, S.J., Kerkhof, L.J., King, G.M., Kling, G.W., Koehler, A.V., Krezalek, M., Kueneman, J., Lamendella, R., Landon, E.M., Lane-deGraaf, K., LaRoche, J., Larsen, P., Laverock, B., Lax, S., Lentino, M., Levin, II, Liancourt, P., Liang, W.J., Linz, A.M., Lipson, D.A., Liu, Y.Q., Lladser, M.E., Lozada, M., Spirito, C.M., MacCormack, W.P., MacRae-Crerar, A., Magris, M., Martin-Platero, A.M., Martin-Vivaldi, M., Martinez, L.M., Martinez-Bueno, M., Marzinelli, E.M., Mason, O.U., Mayer, G.D., McDevitt-Irwin, J.M., McDonald, J.E., McGuire, K.L., McMahon, K.D., McMinds, R., Medina, M., Mendelson, J.R., Metcalf, J.L., Meyer, F., Michelangeli, F., Miller, K., Mills, D.A., Minich, J., Mocali, S., Moitinho-Silva, L., Moore, A., Morgan-Kiss, R.M., Munroe, P., Myrold, D., Neufeld, J.D., Ni, Y.Y., Nicol, G.W., Nielsen, S., Nissimov, J.I., Niu, K.F., Nolan, M.J., Noyce, K., O'Brien, S.L., Okamoto, N., Orlando, L., Castellano, Y.O., Osuolale, O., Oswald, W., Parnell, J., Peralta-Sanchez, J.M., Petraitis, P., Pfister, C., Pilon-Smits, E., Piombino, P., Pointing, S.B., Pollock, F.J., Potter, C., Prithiviraj, B., Quince, C., Rani, A., Ranjan, R., Rao, S., Rees, A.P., Richardson, M., Riebesell, U., Robinson, C., Rockne, K.J., Rodriguezl, S.M., Rohwer, F., Roundstone, W., Safran, R.J., Sangwan, N., Sanz, V., Schrenk, M., Schrenzel, M.D., Scott, N.M., Seger, R.L., Seguin-Orlando, A., Seldin, L., Seyler, L.M., Shakhsheer, B., Sheets, G.M., Shen, C.C., Shi, Y., Shin, H.D., Shogan, B.D., Shutler, D., Siegel, J., Simmons, S., Sjoling, S., Smith, D.P., Soler, J.J., Sperling, M., Steinberg, P.D., Stephens,

B., Stevens, M.A., Taghavi, S., Tai, V., Tait, K., Tan, C.L., Tas, N., Taylor, D.L., Thomas, T., Timling, I., Turner, B.L., Urich, T., Ursell, L.K., van der Lelie, D., Van Treuren, W., van Zwieten, L., Vargas-Robles, D., Thurber, R.V., Vitaglione, P., Walker, D.A., Walters, W.A., Wang, S., Wang, T., Weaver, T., Webster, N.S., Wehrle, B., Weisenhorn, P., Weiss, S., Werner, J.J., West, K., Whitehead, A., Whitehead, S.R., Whittingham, L.A., Willerslev, E., Williams, A.E., Wood, S.A., Woodhams, D.C., Yang, Y.Q., Zaneveld, J., Zarraonaindia, I., Zhang, Q.K., Zhao, H.X., Earth Microbiome Project, C., 2017. A communal catalogue reveals Earth's multiscale microbial diversity. Nature 551, 457-463.

Trivedi, C., Reich, P.B., Maestre, F.T., Hu, H.W., Singh, B.K., Delgado-Baquerizo, M., 2019. Plant-driven niche differentiation of ammonia-oxidizing bacteria and archaea in global drylands. The ISME Journal 13, 2727-2736.

Uritskiy, G.V., DiRuggiero, J., Taylor, J., 2018. MetaWRAP-a flexible pipeline for genome-resolved metagenomic data analysis. Microbiome 6, 158.

van Kessel, M.A., Speth, D.R., Albertsen, M., Nielsen, P.H., Op den Camp, H.J., Kartal, B., Jetten, M.S., Lucker, S., 2015. Complete nitrification by a single microorganism. Nature 528, 555-559.

Verhamme, D.T., Prosser, J.I., Nicol, G.W., 2011. Ammonia concentration determines differential growth of ammonia-oxidising archaea and bacteria in soil microcosms. The ISME Journal 5, 1067-1071.

Wang, B., Qin, W., Ren, Y., Zhou, X., Jung, M.Y., Han, P., Eloe-Fadrosh, E.A., Li, M., Zheng, Y., Lu, L., Yan, X., Ji, J., Liu, Y., Liu, L., Heiner, C., Hall, R., Martens-Habbena, W., Herbold, C.W., Rhee, S.K., Bartlett, D.H., Huang, L., Ingalls, A.E., Wagner, M., Stahl, D.A., Jia, Z., 2019. Expansion of Thaumarchaeota habitat range is correlated with horizontal transfer of ATPase operons. The ISME Journal 13, 3067-3079.

Wu, Y.W., Simmons, B.A., Singer, S.W., 2016. MaxBin 2.0: an automated binning algorithm to recover genomes from multiple metagenomic datasets. Bioinformatics 32, 605-607.

Zhang, L.-M., Hu, H.-W., Shen, J.-P., He, J.-Z., 2012. Ammonia-oxidizing archaea have more important role than ammonia-oxidizing bacteria in ammonia oxidation of strongly acidic soils. The ISME Journal 6, 1032-1045.

Zhang, Y., Qin, W., Hou, L., Zakem, E.J., Wan, X., Zhao, Z., Liu, L., Hunt, K.A., Jiao, N., Kao, S.J., Tang, K., Xie, X., Shen, J., Li, Y., Chen, M., Dai, X., Liu, C., Deng, W., Dai, M., Ingalls, A.E., Stahl, D.A., Herndl, G.J., 2020. Nitrifier adaptation to low energy flux controls inventory of reduced nitrogen in the dark ocean. Proceedings of the National Academy of Sciences of the United States of America 117, 4823-4830.

Zhao, J., Bello, M.O., Meng, Y., Prosser, J.I., Gubry-Rangin, C., 2020a. Selective inhibition of ammonia oxidising archaea by simvastatin stimulates growth of ammonia oxidising bacteria. Soil Biology and Biochemistry 141.

Zhao, J., Meng, Y., Drewer, J., Skiba, U.M., Prosser, J.I., Gubry-Rangin, C., 2020b. Differential ecosystem function stability of ammonia-oxidizing archaea and bacteria following short-term environmental perturbation. mSystems 5.

Table S5. **Proportions of nitrifiers based on normalized marker gene abundances relative to total *rpoB* gene abundance (%).** The proportions of AOA, AOB and comammox abundances were estimated by normalized abundance of *amoA* gene and the canonical *Nitrospira* abundances were estimated by calculating the differences of normalized abundances of *nxrB* gene (total *Nitrospira*) and *amoA* gene of comammox *Nitrospira*). The abundances were normalized by gene length and are based on the assumptions, that each genome contains one *rpoB* gene copy, AOA cells possess one *amoA gene* copy, AOB cells possess 2.5 *amoA* and 2.5 *hao* gene copies, comammox cell possesses 1.5 *amoA* and 1.5 *hao* gene copies, and *Nitrospira* cells possess 4 *nxrB* gene copies.

	AOA	AOB	AOB	Total *Nitrospira*	Comammox *Nitrospira*	canonical *Nitrospira*	Comammox *Nitrospira*	Comammox proportion relative to total *Nitrospira*
	amoA	*amoA*	*hao*	*nxrB*	*amoA*	*nxrB - amoA*	*hao*	
Plot1_July_17	1.55 ± 0.13	0.003 ± 0.002	0.01 ± 0.00	0.29 ± 0.05	0.004 ± 0.002	0.29 ± 0.05	0.004 ± 0.002	1.5 ± 0.8
Plot1_Dec_17	1.17 ± 0.25	0.01 ± 0.00	0.01 ± 0.01	0.39 ± 0.06	0.03 ± 0.02	0.35 ± 0.08	0.02 ± 0.01	10.2 ± 6.3
Plot3_July_17	2.28 ± 0.34	0.02 ± 0.00	0.02 ± 0.00	0.52 ± 0.02	0.06 ± 0.01	0.46 ± 0.02	0.07 ± 0.01	11.9 ± 2.4
Plot3_Dec_17	1.75 ± 0.14	0.02 ± 0.00	0.02 ± 0.00	0.56 ± 0.00	0.12 ± 0.00	0.44 ± 0.00	0.09 ± 0.02	21.4± 0.7
Plot5_July_17	2.32 ± 0.22	0.01 ± 0.00	0.01 ± 0.00	0.48 ± 0.04	0.08 ± 0.01	0.40 ± 0.04	0.07 ± 0.01	17.3 ± 1.4
Plot5_Dec_17	1.90 ± 0.08	0.003 ± 0.002	0.01 ± 0.00	0.69 ± 0.04	0.17 ± 0.04	0.52 ± 0.02	0.16 ± 0.03	24.7 ± 5.1

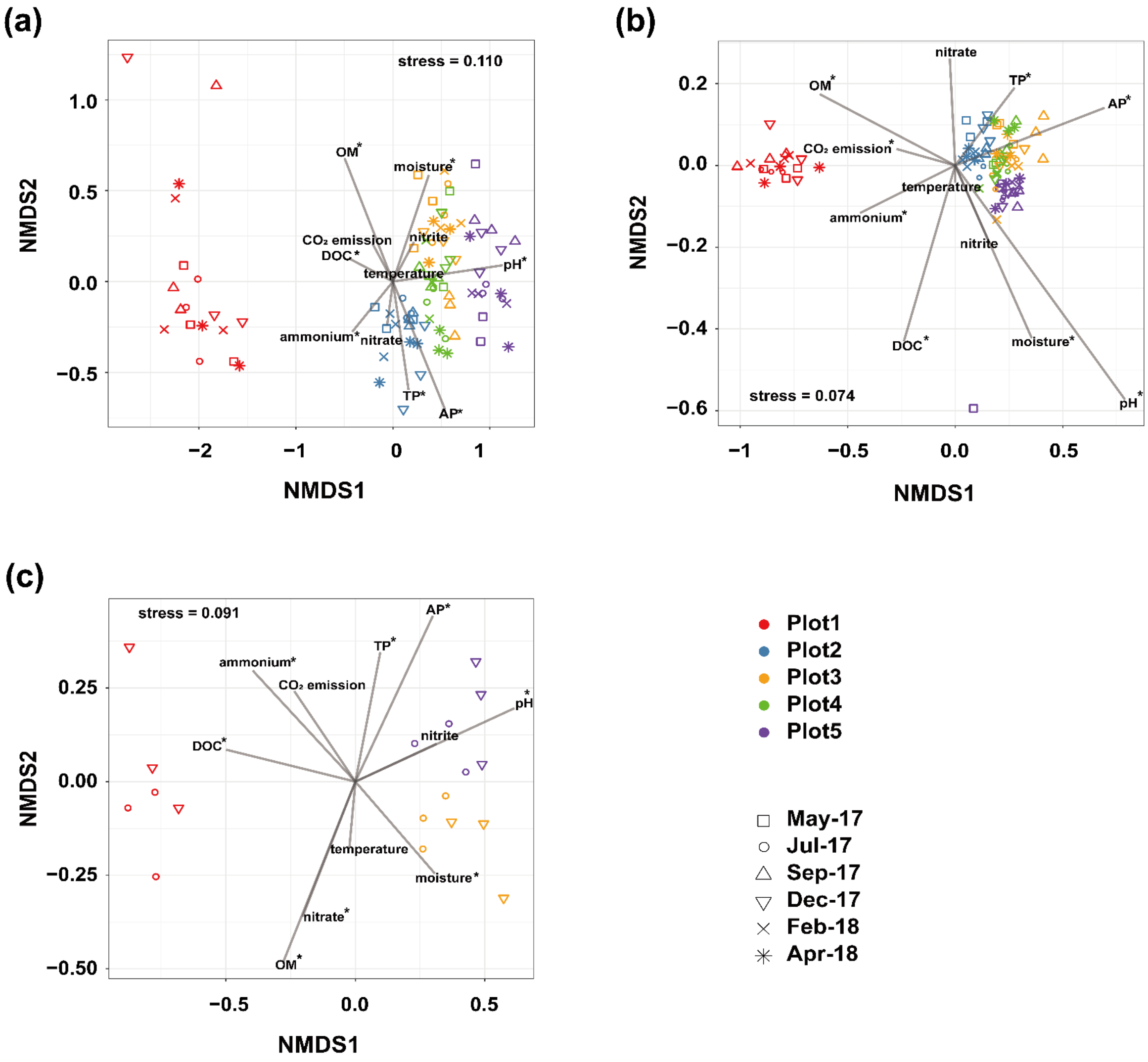

Fig. S1. Nonmetric multidimensional scaling (NMDS) analysis of prokaryotic community composition (bacteria and archaea) in all soils. The analyses were based on (a) the 16S rRNA genes of all prokaryotic community from amplicon sequencing; (b) the 16S rRNA genes of total ammonia oxidizing archaea from amplicon sequencing; (c) the archaeal *amoA* genes from metagenomes. Different symbol colours separate soil plots and different symbol shapes represent different soil collection months. The lengths of the grey line segments within the plot are proportional to the correlations between measured parameters and ordination configuration. The directions of the segments from the center point follow the increasing changes in the values of the parameters. The asterisks (*) indicate significant correlations ($p < 0.05$). OM, organic matter; DOC, dissolved organic carbon; Temperature, soil temperature; TP, total phosphorus; AP, available phosphorus.

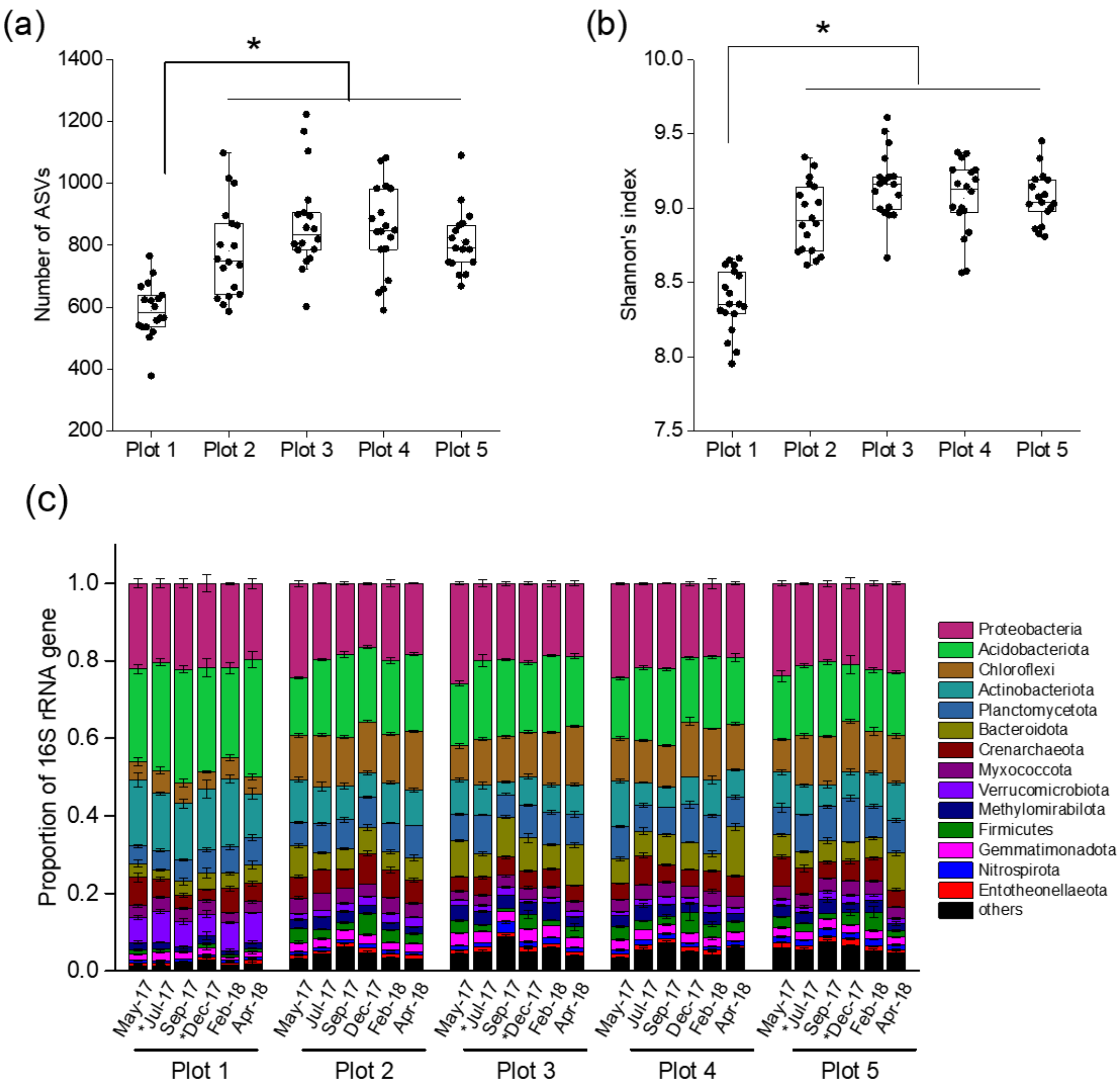

Fig. S2. Number of ASVs (a) and Shannon's index (b) showing the richness and evenness of microbial distribution in different plots and the compositions of microorganisms at phylum level based on 16S rRNA gene classifications (c). (a,b)The index was calculated at sampling depth of 10,000 sequences per sample. The centre line in the box plot represents the median value. The upper and lower boundaries of each box give the 25th and 75th percentiles and the asterisk (*) above the box represents significant difference in index values between plots. (c) Top 14 most abundant phyla (averagely >1% in all soils) were shown in the columns and the rest of the phyla was summed up as "others". The asterisks (*) show soil samples further used for shotgun sequencing. Error bars represent standard errors of relative abundances from triplicate samples.

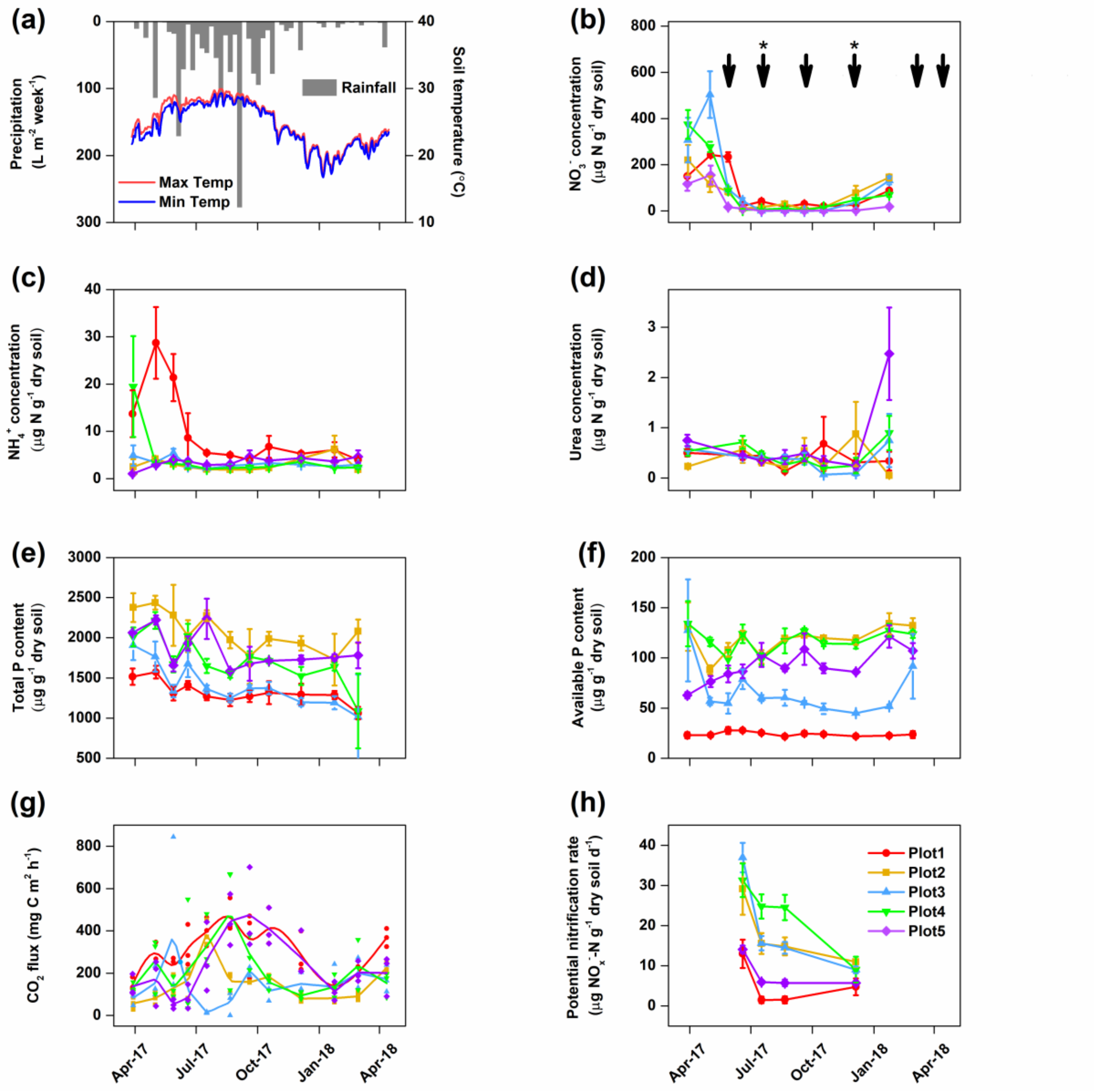

Fig. S3. Annual patterns of climate, soil nutrients, and microbial activities during the 11-month time series. (a) Daily maximum and minimum temperatures at 10-cm soil depth in each plot, and weekly sum of precipitation during the same period. (b)-(d) KCl-extractable soil nitrate, ammonium and urea-N concentrations. (e-f) Soil total and available phosphorus concentrations. (g-h) Soil-atmosphere CO2 fluxes and potential nitrification rates of different soils over time. Arrows in (b) indicate sampling time points for amplicon sequencing of 16S rRNA genes, and arrows with asterisk represent sampling points for shotgun metagenomics and metatranscriptomics sequencing.

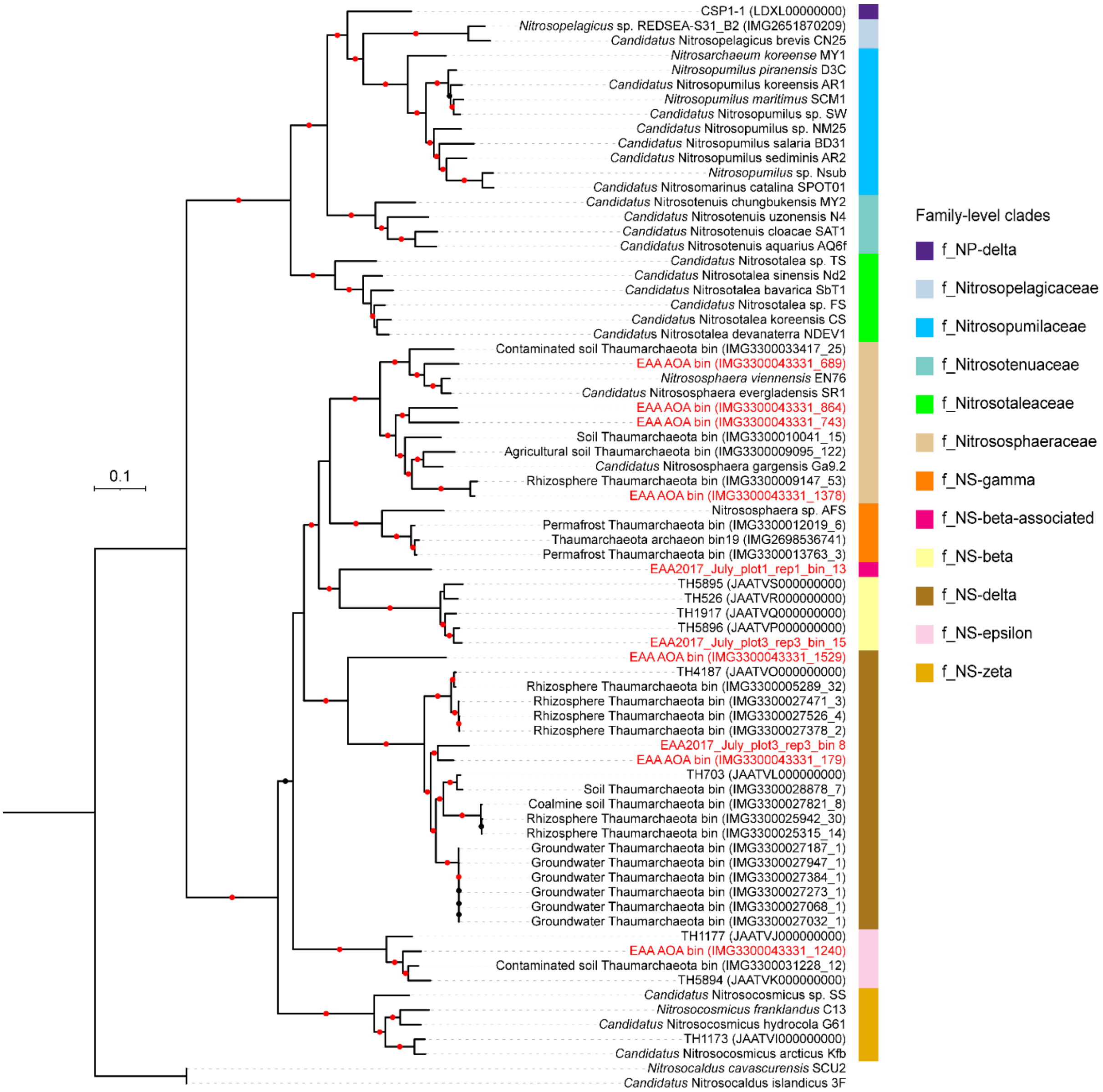

Fig. S4. **Phylogenetic tree constructed from a concatenated alignment of 53 protein sequences by GTDB-Tk v2.1.0.** Reference sequences of cultured AOA genomes and MAGs retrieved from NCBI RefSeq and IMG databases were used to classify each phylotype to a family-level AOA lineage based on genome-wide taxonomic ranking system (Sheridan et al 2020). Ultrafast bootstrap values >70% and >90% (1,000 replicates) are indicated by black and red solid circles, respectively. The scale bar represents 0.1 substitutions per amino acid position. The red genome names are acquired from this study.

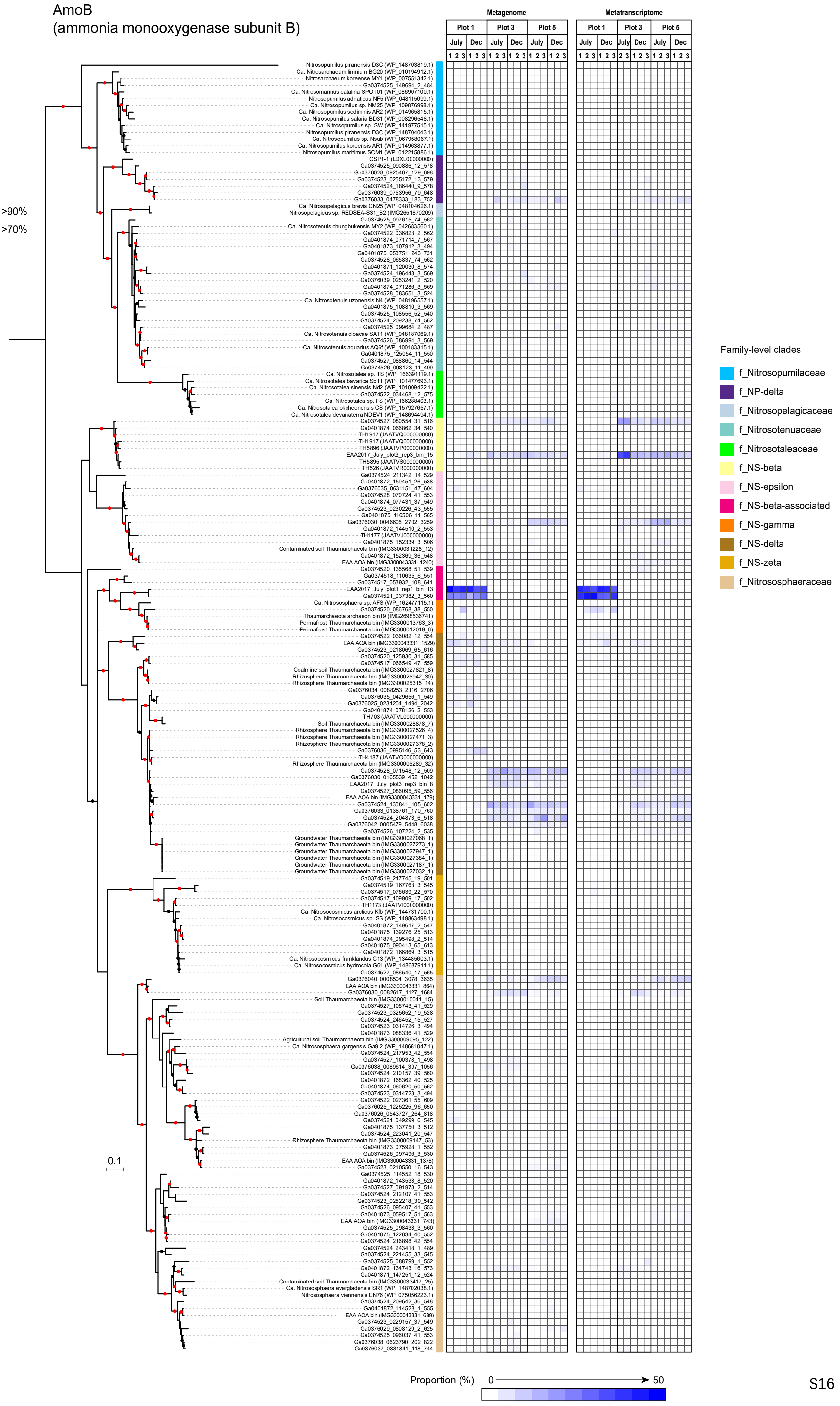
AmoB
(ammonia monooxygenase subunit B)
>90%
>70%
0.1
Metagenome
Metatranscriptome
Plot 1
Plot 3
Plot 5
July
Dec
1 2 3
Family-level clades
f_Nitrosopumilaceae
f_NP-delta
f_Nitrosopelagicaceae
f_Nitrosotenuaceae
f_Nitrosotaleaceae
f_NS-beta
f_NS-epsilon
f_NS-beta-associated
f_NS-gamma
f_NS-delta
f_NS-zeta
f_Nitrososphaeraceae
Proportion (%)
0
50

Fig. S5. Maximum likelihood phylogeny of AOA AmoB protein sequences recovered from metagenomes and metatranscriptomes in this study (left) and heat map of relative abundance based on read mapping (right). Representative sequences assembled in this study are indicated with their IMG accession number ("Ga*"). Reference sequences of cultured AOA genomes and MAGs retrieved from NCBI RefSeq and IMG databases were used to classify each phylotype to a family-level AOA lineage based on genome-wide taxonomic ranking system (Sheridan et al 2020). Ultrafast bootstrap values >70% and >90% (1,000 replicates) are indicated by black and red solid circles, respectively. The scale bar represents 0.1 substitution per amino acid position. Heat map shows the proportion of mapped sequencing reads of each phylotype relative to total AOA *amoB* reads in each metagenome or metatranscriptome. Shown are all three biological replicates, except for plot 3, for which only two metatranscriptomes were recovered in July 2017.

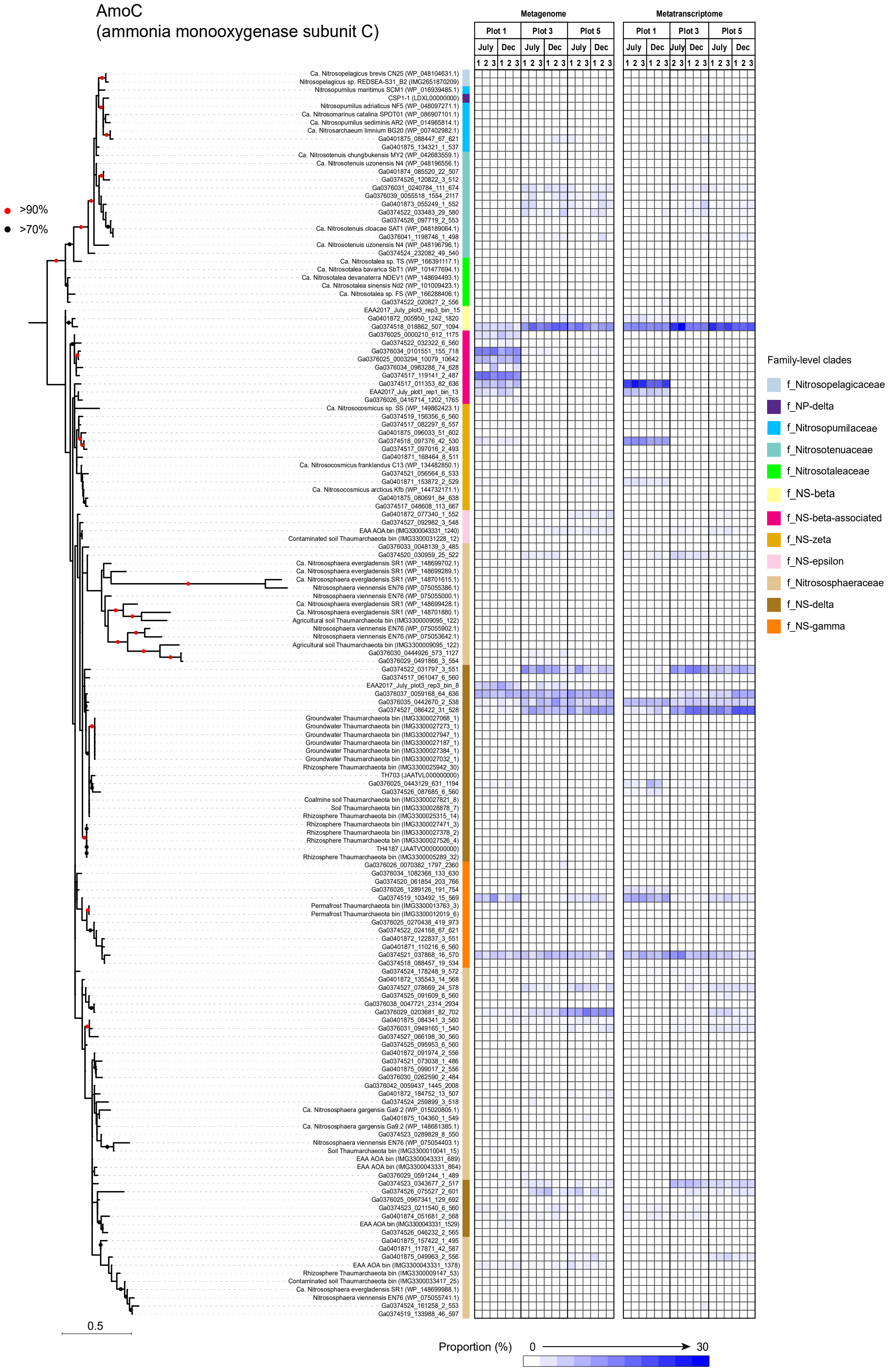
AmoC
(ammonia monooxygenase subunit C)
>90%
>70%
0.5
Metagenome
Metatranscriptome
Plot 1
Plot 3
Plot 5
July
Dec
1 2 3
Family-level clades
f_Nitrosopelagicaceae
f_NP-delta
f_Nitrosopumilaceae
f_Nitrosotenuaceae
f_Nitrosotaleaceae
f_NS-beta
f_NS-beta-associated
f_NS-zeta
f_NS-epsilon
f_Nitrososphaeraceae
f_NS-delta
f_NS-gamma
Proportion (%)
0
30

Fig. S6. Maximum likelihood phylogeny of AOA AmoC protein sequences recovered from metagenomes and metatranscriptomes in this study (left) and heat map of relative abundance based on read mapping (right). Representative sequences assembled in this study are indicated with their IMG accession number ("Ga*"). Reference sequences of cultured AOA genomes and MAGs retrieved from NCBI RefSeq and IMG databases were used to classify each phylotype to a family-level AOA lineage based on genome-wide taxonomic ranking system (Sheridan et al 2020). Ultrafast bootstrap values >70% and >90% (1,000 replicates) are indicated by black and red solid circles, respectively. The scale bar represents 0.5 substitution per amino acid position. Heat map shows the proportion of mapped sequencing reads of each phylotype relative to total AOA *amoC* reads in each metagenome or metatranscriptome. Shown are all three biological replicates, except for plot 3, for which only two metatranscriptomes were recovered in July 2017.

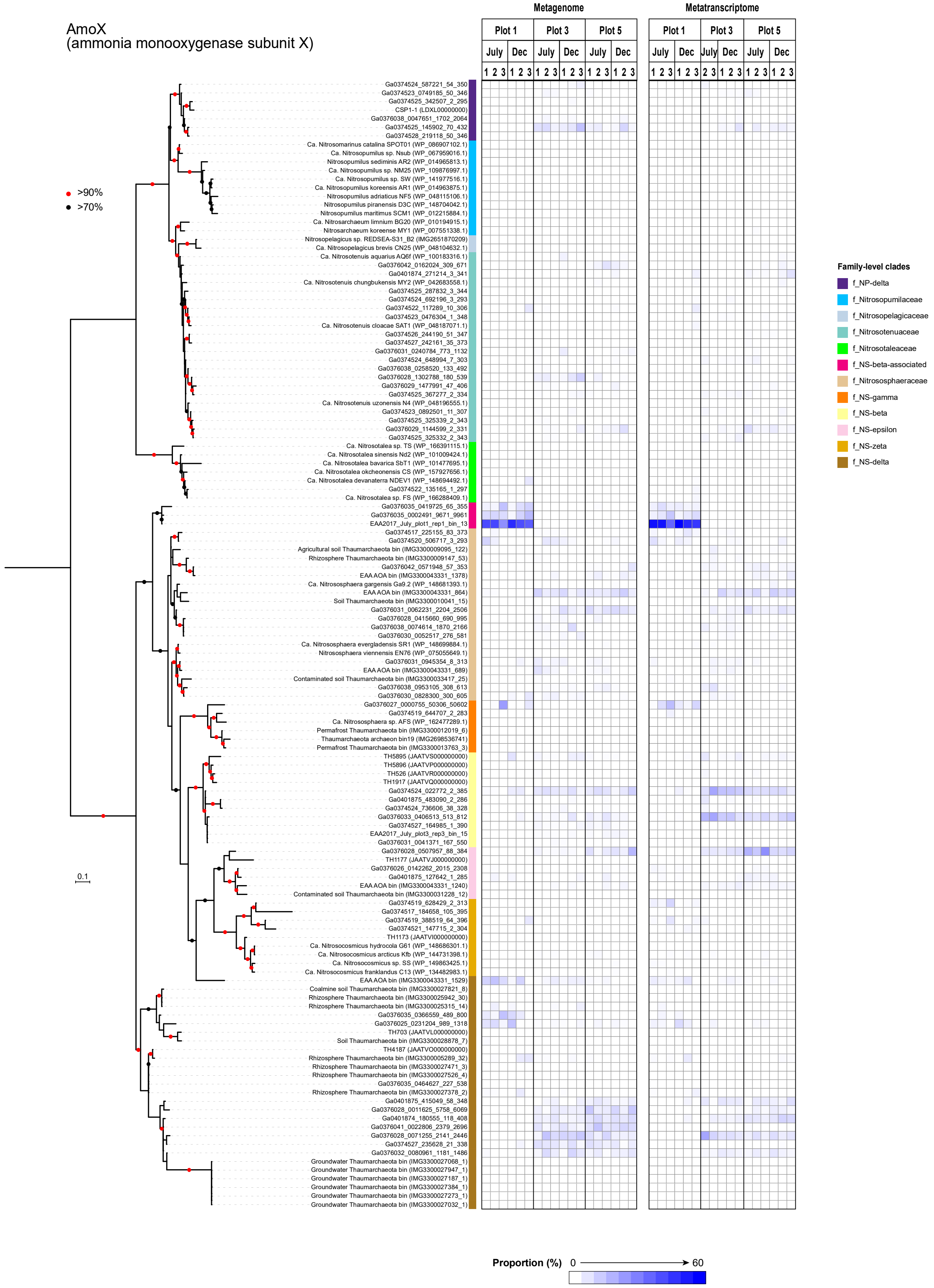
AmoX
(ammonia monooxygenase subunit X)
>90%
>70%
0.1
Metagenome
Metatranscriptome
Plot 1
Plot 3
Plot 5
Plot 1
Plot 3
Plot 5
July
Dec
July
Dec
July
Dec
July
Dec
July
Dec
July
Dec
1 2 3 1 2 3 1 2 3 1 2 3 1 2 3 1 2 3
1 2 3 1 2 3 2 3 1 2 3 1 2 3 1 2 3
Ga0374524_587221_54_350
Ga0374523_0749185_50_346
Ga0374525_342507_2_295
CSP1-1 (LDXL00000000)
Ga0376038_0047651_1702_2064
Ga0374525_145902_70_432
Ga0374528_219118_50_346
Ca. Nitrosomarinus catalina SPOT01 (WP_086907102.1)
Ca. Nitrosopumilus sp. Nsub (WP_067959016.1)
Nitrosopumilus sediminis AR2 (WP_014965813.1)
Ca. Nitrosopumilus sp. NM25 (WP_109876997.1)
Ca. Nitrosopumilus sp. SW (WP_141977516.1)
Ca. Nitrosopumilus koreensis AR1 (WP_014963875.1)
Nitrosopumilus adriaticus NF5 (WP_048115106.1)
Nitrosopumilus piranensis D3C (WP_148704042.1)
Nitrosopumilus maritimus SCM1 (WP_012215884.1)
Ca. Nitrosarchaeum limnium BG20 (WP_010194915.1)
Nitrosarchaeum koreense MY1 (WP_007551338.1)
Nitrosopelagicus sp. REDSEA-S31_B2 (IMG2651870209)
Ca. Nitrosopelagicus brevis CN25 (WP_048104632.1)
Ca. Nitrosotenuis aquarius AQ6f (WP_100183316.1)
Ga0376042_0162024_309_671
Ga0401874_271214_3_341
Ca. Nitrosotenuis chungbukensis MY2 (WP_042683558.1)
Ga0374525_287832_3_344
Ga0374524_692196_3_293
Ga0374522_117289_10_306
Ga0374523_0476304_1_348
Ca. Nitrosotenuis cloacae SAT1 (WP_048187071.1)
Ga0374526_244190_51_347
Ga0374527_242161_35_373
Ga0376031_0240784_773_1132
Ga0374524_648994_7_303
Ga0376038_0258520_133_492
Ga0376028_1302788_180_539
Ga0376029_1477991_47_406
Ga0374525_367277_2_334
Ca. Nitrosotenuis uzonensis N4 (WP_048196555.1)
Ga0374523_0892501_11_307
Ga0374525_325339_2_343
Ga0376029_1144599_2_331
Ga0374525_325332_2_343
Ca. Nitrosotalea sp. TS (WP_166391115.1)
Ca. Nitrosotalea sinensis Nd2 (WP_101009424.1)
Ca. Nitrosotalea bavarica SbT1 (WP_101477695.1)
Ca. Nitrosotalea okcheonensis CS (WP_157927656.1)
Ca. Nitrosotalea devanaterra NDEV1 (WP_148694492.1)
Ga0374522_135165_1_297
Ca. Nitrosotalea sp. FS (WP_166288409.1)
Ga0376035_0419725_65_355
Ga0376035_0002491_9671_9961
EAA2017_July_plot1_rep1_bin_13
Ga0374517_225155_83_373
Ga0374520_506717_3_293
Agricultural soil Thaumarchaeota bin (IMG3300009095_122)
Rhizosphere Thaumarchaeota bin (IMG3300009147_53)
Ga0376042_0571948_57_353
EAA AOA bin (IMG3300043331_1378)
Ca. Nitrososphaera gargensis Ga9.2 (WP_148681393.1)
EAA AOA bin (IMG3300043331_864)
Soil Thaumarchaeota bin (IMG3300010041_15)
Ga0376031_0062231_2204_2506
Ga0376028_0415660_690_995
Ga0376038_0074614_1870_2166
Ga0376030_0052517_276_581
Ca. Nitrososphaera evergladensis SR1 (WP_148699884.1)
Nitrososphaera viennensis EN76 (WP_075055649.1)
Ga0376031_0945354_8_313
EAA AOA bin (IMG3300043331_689)
Contaminated soil Thaumarchaeota bin (IMG3300033417_25)
Ga0376038_0953105_308_613
Ga0376030_0828300_300_605
Ga0376027_0000755_50306_50602
Ga0374519_644707_2_283
Ca. Nitrososphaera sp. AFS (WP_162477289.1)
Permafrost Thaumarchaeota bin (IMG3300012019_6)
Thaumarchaeota archaeon bin19 (IMG2698536741)
Permafrost Thaumarchaeota bin (IMG3300013763_3)
TH5895 (JAATVS000000000)
TH5896 (JAATVP000000000)
TH526 (JAATVR000000000)
TH1917 (JAATVQ000000000)
Ga0374524_022772_2_385
Ga0401875_483090_2_286
Ga0374524_736606_38_328
Ga0376033_0406513_513_812
Ga0374527_164985_1_390
EAA2017_July_plot3_rep3_bin_15
Ga0376031_0041371_167_550
Ga0376028_0507957_88_384
TH1177 (JAATVJ000000000)
Ga0376026_0142262_2015_2308
Ga0401875_127642_1_285
EAA AOA bin (IMG3300043331_1240)
Contaminated soil Thaumarchaeota bin (IMG3300031228_12)
Ga0374519_628429_2_313
Ga0374517_184658_105_395
Ga0374519_388519_64_396
Ga0374521_147715_2_304
TH1173 (JAATVI000000000)
Ca. Nitrosocosmicus hydrocola G61 (WP_148686301.1)
Ca. Nitrosocosmicus arcticus Kfb (WP_144731398.1)
Ca. Nitrosocosmicus sp. SS (WP_149863425.1)
Ca. Nitrosocosmicus franklandus C13 (WP_134482983.1)
EAA AOA bin (IMG3300043331_1529)
Coalmine soil Thaumarchaeota bin (IMG3300027821_8)
Rhizosphere Thaumarchaeota bin (IMG3300025942_30)
Rhizosphere Thaumarchaeota bin (IMG3300025315_14)
Ga0376035_0366559_489_800
Ga0376025_0231204_989_1318
TH703 (JAATVL000000000)
Soil Thaumarchaeota bin (IMG3300028878_7)
TH4187 (JAATVO000000000)
Rhizosphere Thaumarchaeota bin (IMG3300005289_32)
Rhizosphere Thaumarchaeota bin (IMG3300027471_3)
Rhizosphere Thaumarchaeota bin (IMG3300027526_4)
Ga0376035_0464627_227_538
Rhizosphere Thaumarchaeota bin (IMG3300027378_2)
Ga0401875_415049_58_348
Ga0376028_0011625_5758_6069
Ga0401874_180555_118_408
Ga0376041_0022806_2379_2696
Ga0376028_0071255_2141_2446
Ga0374527_235628_21_338
Ga0376032_0080961_1181_1486
Groundwater Thaumarchaeota bin (IMG3300027068_1)
Groundwater Thaumarchaeota bin (IMG3300027947_1)
Groundwater Thaumarchaeota bin (IMG3300027187_1)
Groundwater Thaumarchaeota bin (IMG3300027384_1)
Groundwater Thaumarchaeota bin (IMG3300027273_1)
Groundwater Thaumarchaeota bin (IMG3300027032_1)
Family-level clades
f_NP-delta
f_Nitrosopumilaceae
f_Nitrosopelagicaceae
f_Nitrosotenuaceae
f_Nitrosotaleaceae
f_NS-beta-associated
f_Nitrososphaeraceae
f_NS-gamma
f_NS-beta
f_NS-epsilon
f_NS-zeta
f_NS-delta
Proportion (%) 0 60

Fig. S7. Maximum likelihood phylogeny of AOA AmoX protein sequences recovered from metagenomes and metatranscriptomes in this study (left) and heat map of relative abundance based on read mapping (right). Representative sequences assembled in this study are indicated with their IMG accession number ("Ga*"). Reference sequences of cultured AOA genomes and MAGs retrieved from NCBI RefSeq and IMG databases were used to classify each phylotype to a family-level AOA lineage based on genome-wide taxonomic ranking system (Sheridan et al 2020). Ultrafast bootstrap values >70% and >90% (1,000 replicates) are indicated by black and red solid circles, respectively. The scale bar represents 0.1 substitution per amino acid position. Heat map shows the proportion of mapped sequencing reads of each phylotype relative to total AOA *amoX* reads in each metagenome or metatranscriptome. Shown are all three biological replicates, except for plot 3, for which only two metatranscriptomes were recovered in July 2017.

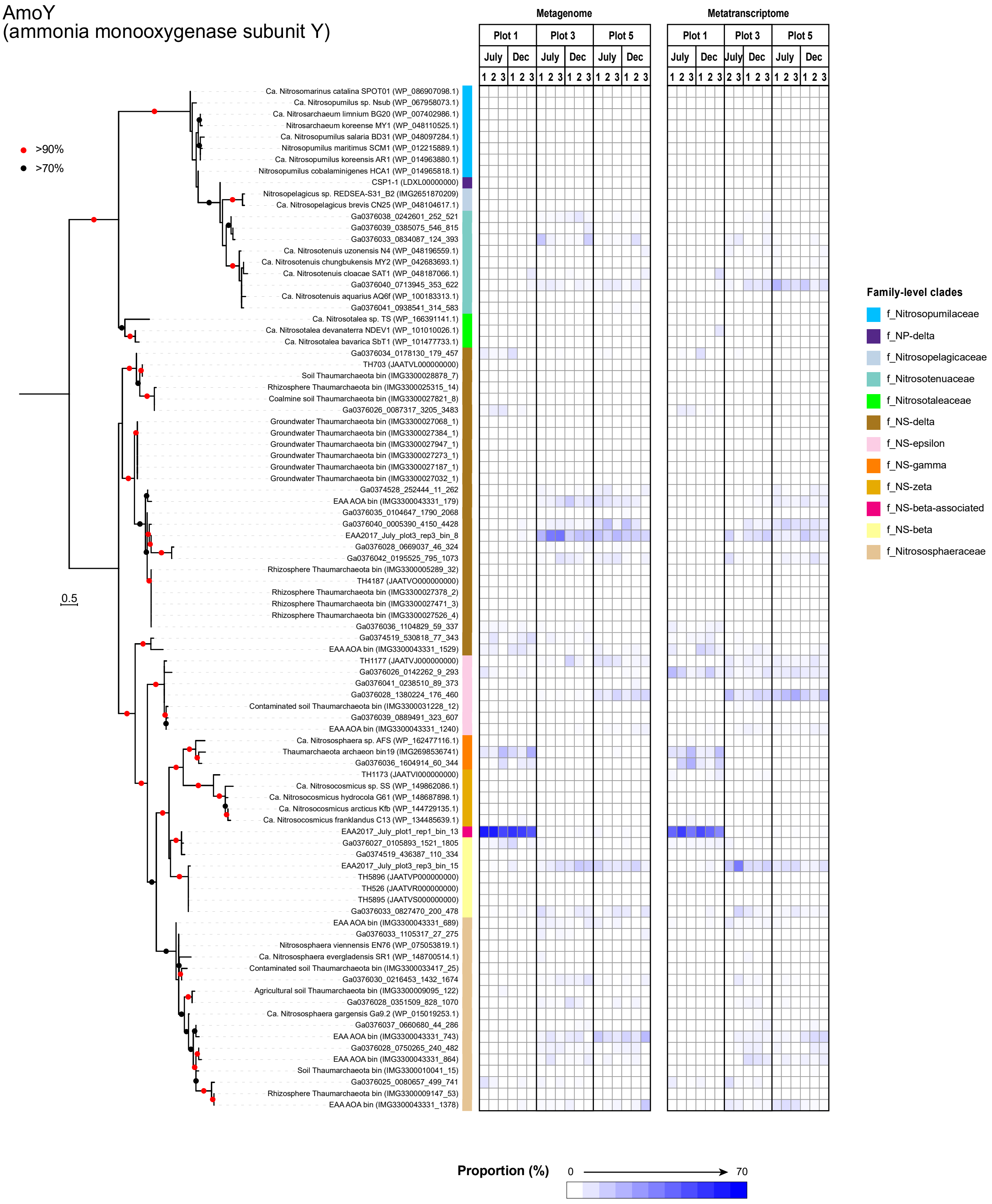
AmoY
(ammonia monooxygenase subunit Y)
>90%
>70%
0.5
Metagenome
Metatranscriptome
Plot 1
Plot 3
Plot 5
July
Dec
1 2 3
Ca. Nitrosomarinus catalina SPOT01 (WP_086907098.1)
Ca. Nitrosopumilus sp. Nsub (WP_067958073.1)
Ca. Nitrosarchaeum limnium BG20 (WP_007402986.1)
Nitrosarchaeum koreense MY1 (WP_048110525.1)
Ca. Nitrosopumilus salaria BD31 (WP_048097284.1)
Nitrosopumilus maritimus SCM1 (WP_012215889.1)
Ca. Nitrosopumilus koreensis AR1 (WP_014963880.1)
Nitrosopumilus cobalaminigenes HCA1 (WP_014965818.1)
CSP1-1 (LDXL00000000)
Nitrosopelagicus sp. REDSEA-S31_B2 (IMG2651870209)
Ca. Nitrosopelagicus brevis CN25 (WP_048104617.1)
Ga0376038_0242601_252_521
Ga0376039_0385075_546_815
Ga0376033_0834087_124_393
Ca. Nitrosotenuis uzonensis N4 (WP_048196559.1)
Ca. Nitrosotenuis chungbukensis MY2 (WP_042683693.1)
Ca. Nitrosotenuis cloacae SAT1 (WP_048187066.1)
Ga0376040_0713945_353_622
Ca. Nitrosotenuis aquarius AQ6f (WP_100183313.1)
Ga0376041_0938541_314_583
Ca. Nitrosotalea sp. TS (WP_166391141.1)
Ca. Nitrosotalea devanaterra NDEV1 (WP_101010026.1)
Ca. Nitrosotalea bavarica SbT1 (WP_101477733.1)
Ga0376034_0178130_179_457
TH703 (JAATVL000000000)
Soil Thaumarchaeota bin (IMG3300028878_7)
Rhizosphere Thaumarchaeota bin (IMG3300025315_14)
Coalmine soil Thaumarchaeota bin (IMG3300027821_8)
Ga0376026_0087317_3205_3483
Groundwater Thaumarchaeota bin (IMG3300027068_1)
Groundwater Thaumarchaeota bin (IMG3300027384_1)
Groundwater Thaumarchaeota bin (IMG3300027947_1)
Groundwater Thaumarchaeota bin (IMG3300027273_1)
Groundwater Thaumarchaeota bin (IMG3300027187_1)
Groundwater Thaumarchaeota bin (IMG3300027032_1)
Ga0374528_252444_11_262
EAA AOA bin (IMG3300043331_179)
Ga0376035_0104647_1790_2068
Ga0376040_0005390_4150_4428
EAA2017_July_plot3_rep3_bin_8
Ga0376028_0669037_46_324
Ga0376042_0195525_795_1073
Rhizosphere Thaumarchaeota bin (IMG3300005289_32)
TH4187 (JAATVO000000000)
Rhizosphere Thaumarchaeota bin (IMG3300027378_2)
Rhizosphere Thaumarchaeota bin (IMG3300027471_3)
Rhizosphere Thaumarchaeota bin (IMG3300027526_4)
Ga0376036_1104829_59_337
Ga0374519_530818_77_343
EAA AOA bin (IMG3300043331_1529)
TH1177 (JAATVJ000000000)
Ga0376026_0142262_9_293
Ga0376041_0238510_89_373
Ga0376028_1380224_176_460
Contaminated soil Thaumarchaeota bin (IMG3300031228_12)
Ga0376039_0889491_323_607
EAA AOA bin (IMG3300043331_1240)
Ca. Nitrososphaera sp. AFS (WP_162477116.1)
Thaumarchaeota archaeon bin19 (IMG2698536741)
Ga0376036_1604914_60_344
TH1173 (JAATVI000000000)
Ca. Nitrosocosmicus sp. SS (WP_149862086.1)
Ca. Nitrosocosmicus hydrocola G61 (WP_148687898.1)
Ca. Nitrosocosmicus arcticus Kfb (WP_144729135.1)
Ca. Nitrosocosmicus franklandus C13 (WP_134485639.1)
EAA2017_July_plot1_rep1_bin_13
Ga0376027_0105893_1521_1805
Ga0374519_436387_110_334
EAA2017_July_plot3_rep3_bin_15
TH5896 (JAATVP000000000)
TH526 (JAATVR000000000)
TH5895 (JAATVS000000000)
Ga0376033_0827470_200_478
EAA AOA bin (IMG3300043331_689)
Ga0376033_1105317_27_275
Nitrososphaera viennensis EN76 (WP_075053819.1)
Ca. Nitrososphaera evergladensis SR1 (WP_148700514.1)
Contaminated soil Thaumarchaeota bin (IMG3300033417_25)
Ga0376030_0216453_1432_1674
Agricultural soil Thaumarchaeota bin (IMG3300009095_122)
Ga0376028_0351509_828_1070
Ca. Nitrososphaera gargensis Ga9.2 (WP_015019253.1)
Ga0376037_0660680_44_286
EAA AOA bin (IMG3300043331_743)
Ga0376028_0750265_240_482
EAA AOA bin (IMG3300043331_864)
Soil Thaumarchaeota bin (IMG3300010041_15)
Ga0376025_0080657_499_741
Rhizosphere Thaumarchaeota bin (IMG3300009147_53)
EAA AOA bin (IMG3300043331_1378)
Family-level clades
f_Nitrosopumilaceae
f_NP-delta
f_Nitrosopelagicaceae
f_Nitrosotenuaceae
f_Nitrosotaleaceae
f_NS-delta
f_NS-epsilon
f_NS-gamma
f_NS-zeta
f_NS-beta-associated
f_NS-beta
f_Nitrososphaeraceae
Proportion (%)
0
70

Fig. S8. Maximum likelihood phylogeny of AOA AmoY protein sequences recovered from metagenomes and metatranscriptomes in this study (left) and heat map of relative abundance based on read mapping (right). Representative sequences assembled in this study are indicated with their IMG accession number ("Ga*"). Reference sequences of cultured AOA genomes and MAGs retrieved from NCBI RefSeq and IMG databases were used to classify each phylotype to a family-level AOA lineage based on genome-wide taxonomic ranking system (Sheridan et al 2020). Ultrafast bootstrap values >70% and >90% (1,000 replicates) are indicated by black and red solid circles, respectively. The scale bar represents 0.5 substitution per amino acid position. Heat map shows the proportion of mapped sequencing reads of each phylotype relative to total AOA *amoY* reads in each metagenome or metatranscriptome. Shown are all three biological replicates, except for plot 3, for which only two metatranscriptomes were recovered in July 2017.

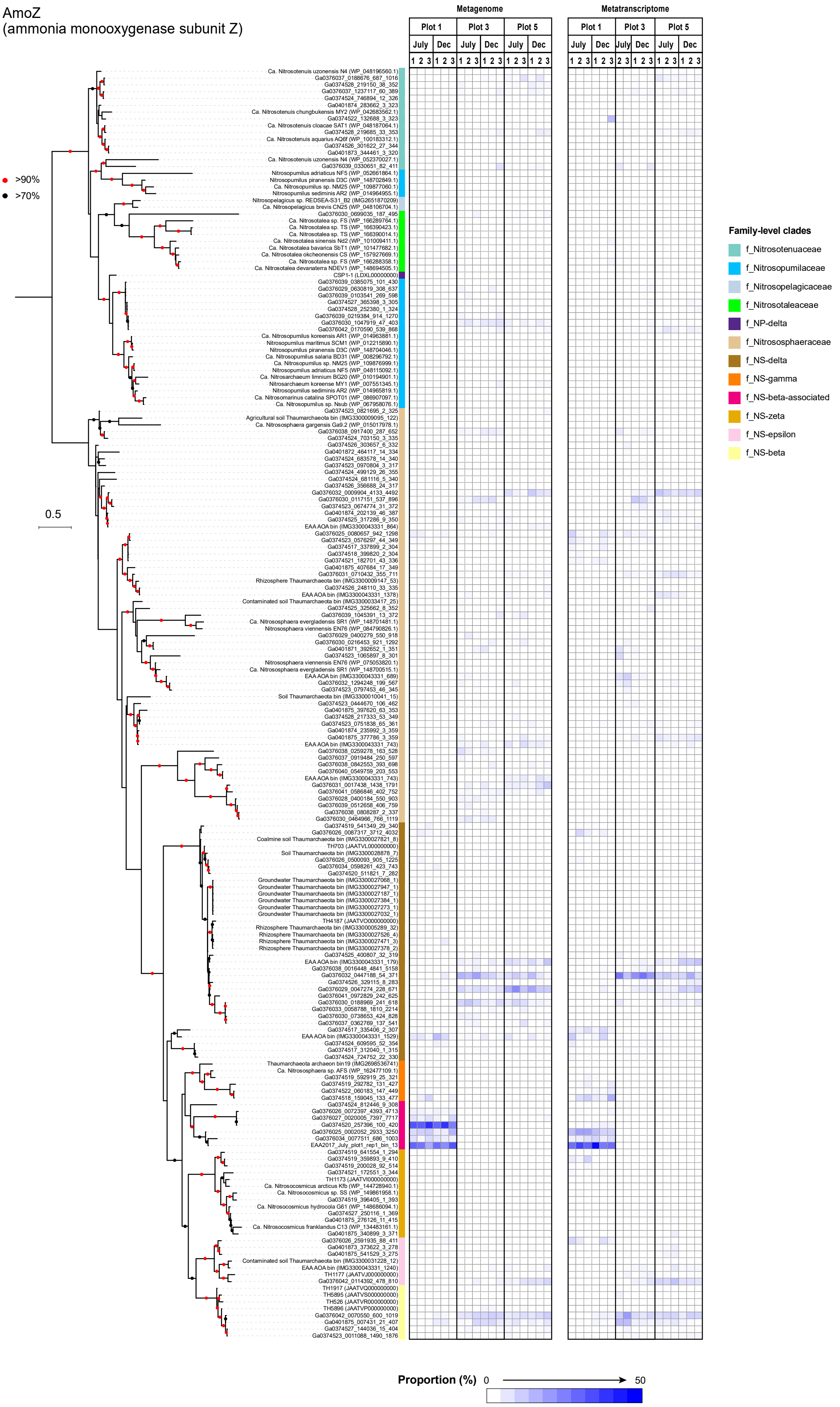
AmoZ
(ammonia monooxygenase subunit Z)
>90%
>70%
0.5
Metagenome
Metatranscriptome
Plot 1
Plot 3
Plot 5
July
Dec
Family-level clades
f_Nitrosotenuaceae
f_Nitrosopumilaceae
f_Nitrosopelagicaceae
f_Nitrosotaleaceae
f_NP-delta
f_Nitrososphaeraceae
f_NS-delta
f_NS-gamma
f_NS-beta-associated
f_NS-zeta
f_NS-epsilon
f_NS-beta
Proportion (%)
0
50

Fig. S9. Maximum likelihood phylogeny of AOA AmoZ protein sequences recovered from metagenomes and metatranscriptomes in this study (left) and heat map of relative abundance based on read mapping (right). Representative sequences assembled in this study are indicated with their IMG accession number ("Ga*"). Reference sequences of cultured AOA genomes and MAGs retrieved from NCBI RefSeq and IMG databases were used to classify each phylotype to a family-level AOA lineage based on genome-wide taxonomic ranking system (Sheridan et al 2020). Ultrafast bootstrap values >70% and >90% (1,000 replicates) are indicated by black and red solid circles, respectively. The scale bar represents 0.5 substitution per amino acid position. Heat map shows the proportion of mapped sequencing reads of each phylotype relative to total AOA *amoZ* reads in each metagenome or metatranscriptome. Shown are all three biological replicates, except for plot 3, for which only two metatranscriptomes were recovered in July 2017.

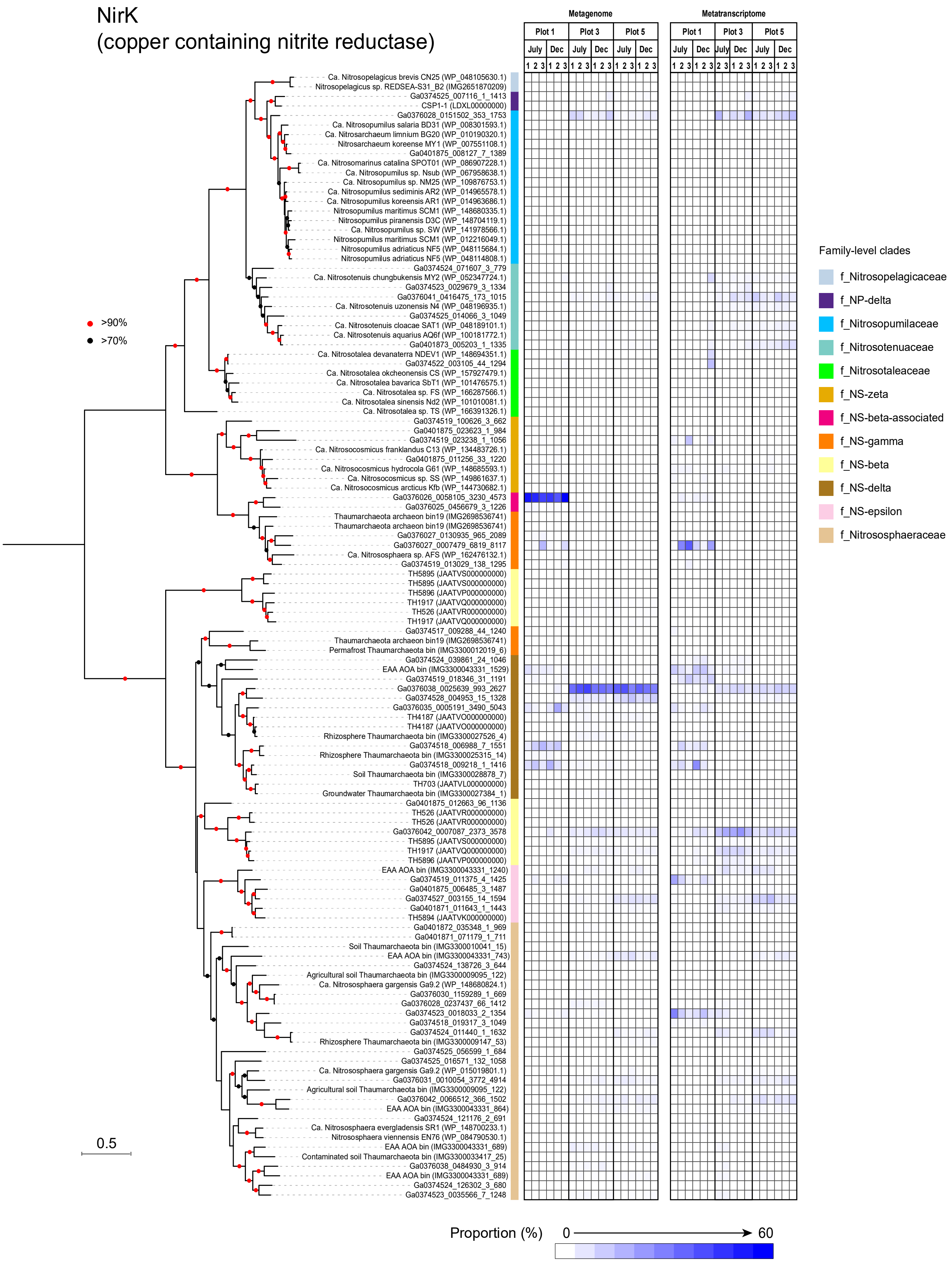
NirK
(copper containing nitrite reductase)
Metagenome
Metatranscriptome
Plot 1
Plot 3
Plot 5
July
Dec
1 2 3
>90%
>70%
0.5
Ca. Nitrosopelagicus brevis CN25 (WP_048105630.1)
Nitrosopelagicus sp. REDSEA-S31_B2 (IMG2651870209)
Ga0374525_007116_1_1413
CSP1-1 (LDXL00000000)
Ga0376028_0151502_353_1753
Ca. Nitrosopumilus salaria BD31 (WP_008301593.1)
Ca. Nitrosarchaeum limnium BG20 (WP_010190320.1)
Nitrosarchaeum koreense MY1 (WP_007551108.1)
Ga0401875_008127_7_1389
Ca. Nitrosomarinus catalina SPOT01 (WP_086907228.1)
Ca. Nitrosopumilus sp. Nsub (WP_067958638.1)
Ca. Nitrosopumilus sp. NM25 (WP_109876753.1)
Ca. Nitrosopumilus sediminis AR2 (WP_014965578.1)
Ca. Nitrosopumilus koreensis AR1 (WP_014963686.1)
Nitrosopumilus maritimus SCM1 (WP_148680335.1)
Nitrosopumilus piranensis D3C (WP_148704119.1)
Ca. Nitrosopumilus sp. SW (WP_141978566.1)
Nitrosopumilus maritimus SCM1 (WP_012216049.1)
Nitrosopumilus adriaticus NF5 (WP_048115684.1)
Nitrosopumilus adriaticus NF5 (WP_048114808.1)
Ga0374524_071607_3_779
Ca. Nitrosotenuis chungbukensis MY2 (WP_052347724.1)
Ga0374523_0029679_3_1334
Ga0376041_0416475_173_1015
Ca. Nitrosotenuis uzonensis N4 (WP_048196935.1)
Ga0374525_014066_3_1049
Ca. Nitrosotenuis cloacae SAT1 (WP_048189101.1)
Ca. Nitrosotenuis aquarius AQ6f (WP_100181772.1)
Ga0401873_005203_1_1335
Ca. Nitrosotalea devanaterra NDEV1 (WP_148694351.1)
Ga0374522_003105_44_1294
Ca. Nitrosotalea okcheonensis CS (WP_157927479.1)
Ca. Nitrosotalea bavarica SbT1 (WP_101476575.1)
Ca. Nitrosotalea sp. FS (WP_166287566.1)
Ca. Nitrosotalea sinensis Nd2 (WP_101010081.1)
Ca. Nitrosotalea sp. TS (WP_166391326.1)
Ga0374519_100626_3_662
Ga0401875_023623_1_984
Ga0374519_023238_1_1056
Ca. Nitrosocosmicus franklandus C13 (WP_134483726.1)
Ga0401875_011256_33_1220
Ca. Nitrosocosmicus hydrocola G61 (WP_148685593.1)
Ca. Nitrosocosmicus sp. SS (WP_149861637.1)
Ca. Nitrosocosmicus arcticus Kfb (WP_144730682.1)
Ga0376026_0058105_3230_4573
Ga0376025_0456679_3_1226
Thaumarchaeota archaeon bin19 (IMG2698536741)
Thaumarchaeota archaeon bin19 (IMG2698536741)
Ga0376027_0130935_965_2089
Ga0376027_0007479_6819_8117
Ca. Nitrososphaera sp. AFS (WP_162476132.1)
Ga0374519_013029_138_1295
TH5895 (JAATVS000000000)
TH5895 (JAATVS000000000)
TH5896 (JAATVP000000000)
TH1917 (JAATVQ000000000)
TH526 (JAATVR000000000)
TH1917 (JAATVQ000000000)
Ga0374517_009288_44_1240
Thaumarchaeota archaeon bin19 (IMG2698536741)
Permafrost Thaumarchaeota bin (IMG3300012019_6)
Ga0374524_039861_24_1046
EAA AOA bin (IMG3300043331_1529)
Ga0374519_018346_31_1191
Ga0376038_0025639_993_2627
Ga0374528_004953_15_1328
Ga0376035_0005191_3490_5043
TH4187 (JAATVO000000000)
TH4187 (JAATVO000000000)
Rhizosphere Thaumarchaeota bin (IMG3300027526_4)
Ga0374518_006988_7_1551
Rhizosphere Thaumarchaeota bin (IMG3300025315_14)
Ga0374518_009218_1_1416
Soil Thaumarchaeota bin (IMG3300028878_7)
TH703 (JAATVL000000000)
Groundwater Thaumarchaeota bin (IMG3300027384_1)
Ga0401875_012663_96_1136
TH526 (JAATVR000000000)
TH526 (JAATVR000000000)
Ga0376042_0007087_2373_3578
TH5895 (JAATVS000000000)
TH1917 (JAATVQ000000000)
TH5896 (JAATVP000000000)
EAA AOA bin (IMG3300043331_1240)
Ga0374519_011375_4_1425
Ga0401875_006485_3_1487
Ga0374527_003155_14_1594
Ga0401871_011643_1_1443
TH5894 (JAATVK000000000)
Ga0401872_035348_1_969
Ga0401871_071179_1_711
Soil Thaumarchaeota bin (IMG3300010041_15)
EAA AOA bin (IMG3300043331_743)
Ga0374524_138726_3_644
Agricultural soil Thaumarchaeota bin (IMG3300009095_122)
Ca. Nitrososphaera gargensis Ga9.2 (WP_148680824.1)
Ga0376030_1159289_1_669
Ga0376028_0237437_66_1412
Ga0374523_0018033_2_1354
Ga0374518_019317_3_1049
Ga0374524_011440_1_1632
Rhizosphere Thaumarchaeota bin (IMG3300009147_53)
Ga0374525_056599_1_684
Ga0374525_016571_132_1058
Ca. Nitrososphaera gargensis Ga9.2 (WP_015019801.1)
Ga0376031_0010054_3772_4914
Agricultural soil Thaumarchaeota bin (IMG3300009095_122)
Ga0376042_0066512_366_1502
EAA AOA bin (IMG3300043331_864)
Ga0374524_121176_2_691
Ca. Nitrososphaera evergladensis SR1 (WP_148700233.1)
Nitrososphaera viennensis EN76 (WP_084790530.1)
EAA AOA bin (IMG3300043331_689)
Contaminated soil Thaumarchaeota bin (IMG3300033417_25)
Ga0376038_0484930_3_914
EAA AOA bin (IMG3300043331_689)
Ga0374524_126302_3_680
Ga0374523_0035566_7_1248
Family-level clades
f_Nitrosopelagicaceae
f_NP-delta
f_Nitrosopumilaceae
f_Nitrosotenuaceae
f_Nitrosotaleaceae
f_NS-zeta
f_NS-beta-associated
f_NS-gamma
f_NS-beta
f_NS-delta
f_NS-epsilon
f_Nitrososphaeraceae
Proportion (%)
0
60

Fig. S10. Maximum likelihood phylogeny of AOA NirK protein sequences recovered from metagenomes and metatranscriptomes in this study (left) and heat map of relative abundance based on read mapping (right). Representative sequences assembled in this study are indicated with their IMG accession number ("Ga*"). Reference sequences of cultured AOA genomes and MAGs retrieved from NCBI RefSeq and IMG databases were used to classify each phylotype to a family-level AOA lineage based on genome-wide taxonomic ranking system (Sheridan et al 2020). Ultrafast bootstrap values >70% and >90% (1,000 replicates) are indicated by black and red solid circles, respectively. The scale bar represents 0.5 substitution per amino acid position. Heat map shows the proportion of mapped sequencing reads of each phylotype relative to total AOA *nirK* reads in each metagenome or metatranscriptome. Shown are all three biological replicates, except for plot 3, for which only two metatranscriptomes were recovered in July 2017.

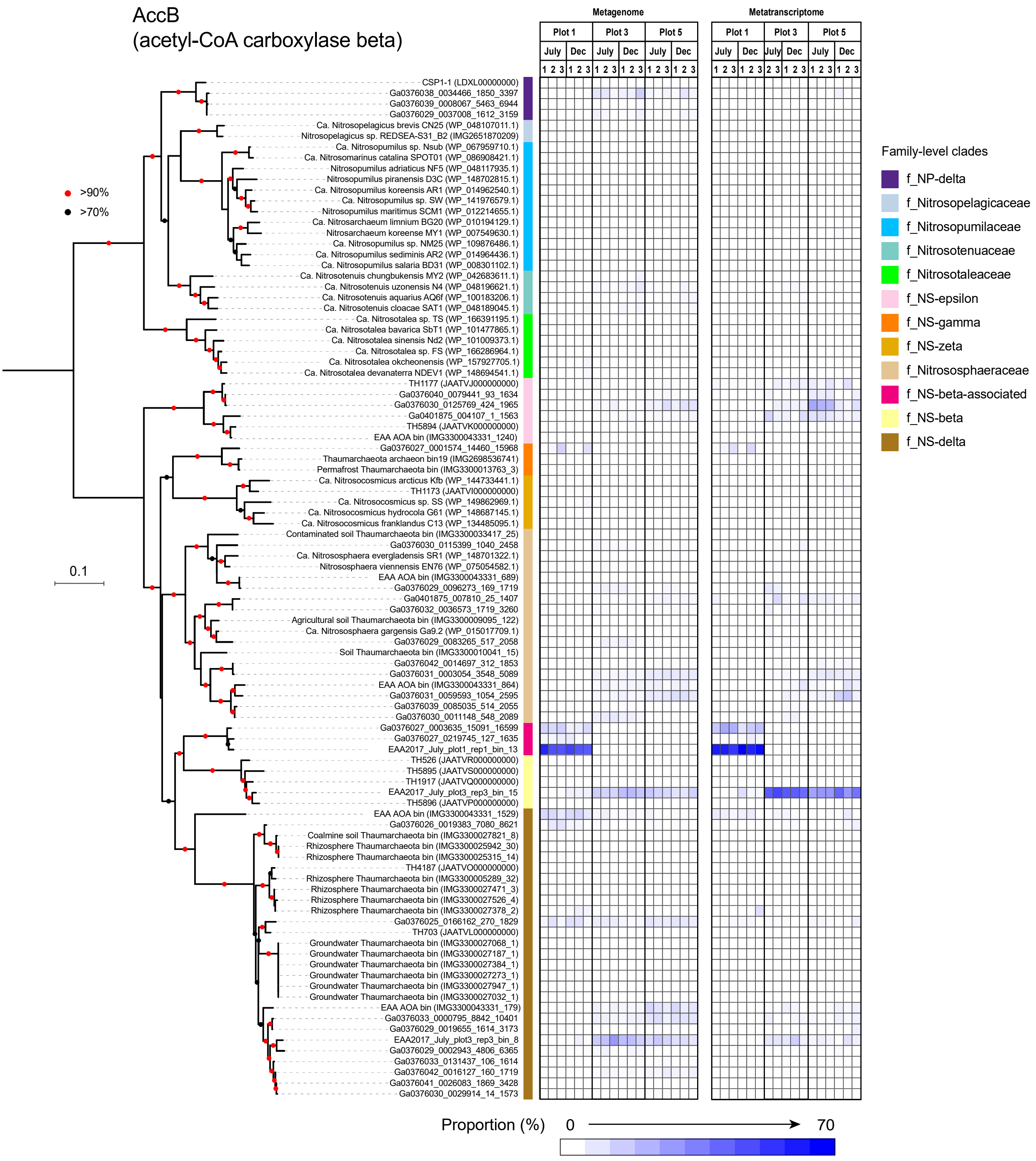
AccB
(acetyl-CoA carboxylase beta)
>90%
>70%
0.1
Metagenome
Metatranscriptome
Plot 1
Plot 3
Plot 5
July
Dec
1 2 3
CSP1-1 (LDXL00000000)
Ga0376038_0034466_1850_3397
Ga0376039_0008067_5463_6944
Ga0376029_0037008_1612_3159
Ca. Nitrosopelagicus brevis CN25 (WP_048107011.1)
Nitrosopelagicus sp. REDSEA-S31_B2 (IMG2651870209)
Ca. Nitrosopumilus sp. Nsub (WP_067959710.1)
Ca. Nitrosomarinus catalina SPOT01 (WP_086908421.1)
Nitrosopumilus adriaticus NF5 (WP_048117935.1)
Nitrosopumilus piranensis D3C (WP_148702815.1)
Ca. Nitrosopumilus koreensis AR1 (WP_014962540.1)
Ca. Nitrosopumilus sp. SW (WP_141976579.1)
Nitrosopumilus maritimus SCM1 (WP_012214655.1)
Ca. Nitrosarchaeum limnium BG20 (WP_010194129.1)
Nitrosarchaeum koreense MY1 (WP_007549630.1)
Ca. Nitrosopumilus sp. NM25 (WP_109876486.1)
Ca. Nitrosopumilus sediminis AR2 (WP_014964436.1)
Ca. Nitrosopumilus salaria BD31 (WP_008301102.1)
Ca. Nitrosotenuis chungbukensis MY2 (WP_042683611.1)
Ca. Nitrosotenuis uzonensis N4 (WP_048196621.1)
Ca. Nitrosotenuis aquarius AQ6f (WP_100183206.1)
Ca. Nitrosotenuis cloacae SAT1 (WP_048189045.1)
Ca. Nitrosotalea sp. TS (WP_166391195.1)
Ca. Nitrosotalea bavarica SbT1 (WP_101477865.1)
Ca. Nitrosotalea sinensis Nd2 (WP_101009373.1)
Ca. Nitrosotalea sp. FS (WP_166286964.1)
Ca. Nitrosotalea okcheonensis (WP_157927705.1)
Ca. Nitrosotalea devanaterra NDEV1 (WP_148694541.1)
TH1177 (JAATVJ000000000)
Ga0376040_0079441_93_1634
Ga0376030_0125769_424_1965
Ga0401875_004107_1_1563
TH5894 (JAATVK000000000)
EAA AOA bin (IMG3300043331_1240)
Ga0376027_0001574_14460_15968
Thaumarchaeota archaeon bin19 (IMG2698536741)
Permafrost Thaumarchaeota bin (IMG3300013763_3)
Ca. Nitrosocosmicus arcticus Kfb (WP_144733441.1)
TH1173 (JAATVI000000000)
Ca. Nitrosocosmicus sp. SS (WP_149862969.1)
Ca. Nitrosocosmicus hydrocola G61 (WP_148687145.1)
Ca. Nitrosocosmicus franklandus C13 (WP_134485095.1)
Contaminated soil Thaumarchaeota bin (IMG3300033417_25)
Ga0376030_0115399_1040_2458
Ca. Nitrososphaera evergladensis SR1 (WP_148701322.1)
Nitrososphaera viennensis EN76 (WP_075054582.1)
EAA AOA bin (IMG3300043331_689)
Ga0376029_0096273_169_1719
Ga0401875_007810_25_1407
Ga0376032_0036573_1719_3260
Agricultural soil Thaumarchaeota bin (IMG3300009095_122)
Ca. Nitrososphaera gargensis Ga9.2 (WP_015017709.1)
Ga0376029_0083265_517_2058
Soil Thaumarchaeota bin (IMG3300010041_15)
Ga0376042_0014697_312_1853
Ga0376031_0003054_3548_5089
EAA AOA bin (IMG3300043331_864)
Ga0376031_0059593_1054_2595
Ga0376039_0085035_514_2055
Ga0376030_0011148_548_2089
Ga0376027_0003635_15091_16599
Ga0376027_0219745_127_1635
EAA2017_July_plot1_rep1_bin_13
TH526 (JAATVR000000000)
TH5895 (JAATVS000000000)
TH1917 (JAATVQ000000000)
EAA2017_July_plot3_rep3_bin_15
TH5896 (JAATVP000000000)
EAA AOA bin (IMG3300043331_1529)
Ga0376026_0019383_7080_8621
Coalmine soil Thaumarchaeota bin (IMG3300027821_8)
Rhizosphere Thaumarchaeota bin (IMG3300025942_30)
Rhizosphere Thaumarchaeota bin (IMG3300025315_14)
TH4187 (JAATVO000000000)
Rhizosphere Thaumarchaeota bin (IMG3300005289_32)
Rhizosphere Thaumarchaeota bin (IMG3300027471_3)
Rhizosphere Thaumarchaeota bin (IMG3300027526_4)
Rhizosphere Thaumarchaeota bin (IMG3300027378_2)
Ga0376025_0166162_270_1829
TH703 (JAATVL000000000)
Groundwater Thaumarchaeota bin (IMG3300027068_1)
Groundwater Thaumarchaeota bin (IMG3300027187_1)
Groundwater Thaumarchaeota bin (IMG3300027384_1)
Groundwater Thaumarchaeota bin (IMG3300027273_1)
Groundwater Thaumarchaeota bin (IMG3300027947_1)
Groundwater Thaumarchaeota bin (IMG3300027032_1)
EAA AOA bin (IMG3300043331_179)
Ga0376033_0000795_8842_10401
Ga0376029_0019655_1614_3173
EAA2017_July_plot3_rep3_bin_8
Ga0376029_0002943_4806_6365
Ga0376033_0131437_106_1614
Ga0376042_0016127_160_1719
Ga0376041_0026083_1869_3428
Ga0376030_0029914_14_1573
Family-level clades
f_NP-delta
f_Nitrosopelagicaceae
f_Nitrosopumilaceae
f_Nitrosotenuaceae
f_Nitrosotaleaceae
f_NS-epsilon
f_NS-gamma
f_NS-zeta
f_Nitrososphaeraceae
f_NS-beta-associated
f_NS-beta
f_NS-delta
Proportion (%)
0
70

Fig. S11. Maximum likelihood phylogeny of AOA AccB protein sequences recovered from metagenomes and metatranscriptomes in this study (left) and heat map of relative abundance based on read mapping (right). Representative sequences assembled in this study are indicated with their IMG accession number ("Ga*"). Reference sequences of cultured AOA genomes and MAGs retrieved from NCBI RefSeq and IMG databases were used to classify each phylotype to a family-level AOA lineage based on genome-wide taxonomic ranking system (Sheridan et al 2020). Ultrafast bootstrap values >70% and >90% (1,000 replicates) are indicated by black and red solid circles, respectively. The scale bar represents 0.1 substitution per amino acid position. Heat map shows the proportion of mapped sequencing reads of each phylotype relative to total AOA *accB* reads in each metagenome or metatranscriptome. Shown are all three biological replicates, except for plot 3, for which only two metatranscriptomes were recovered in July 2017.

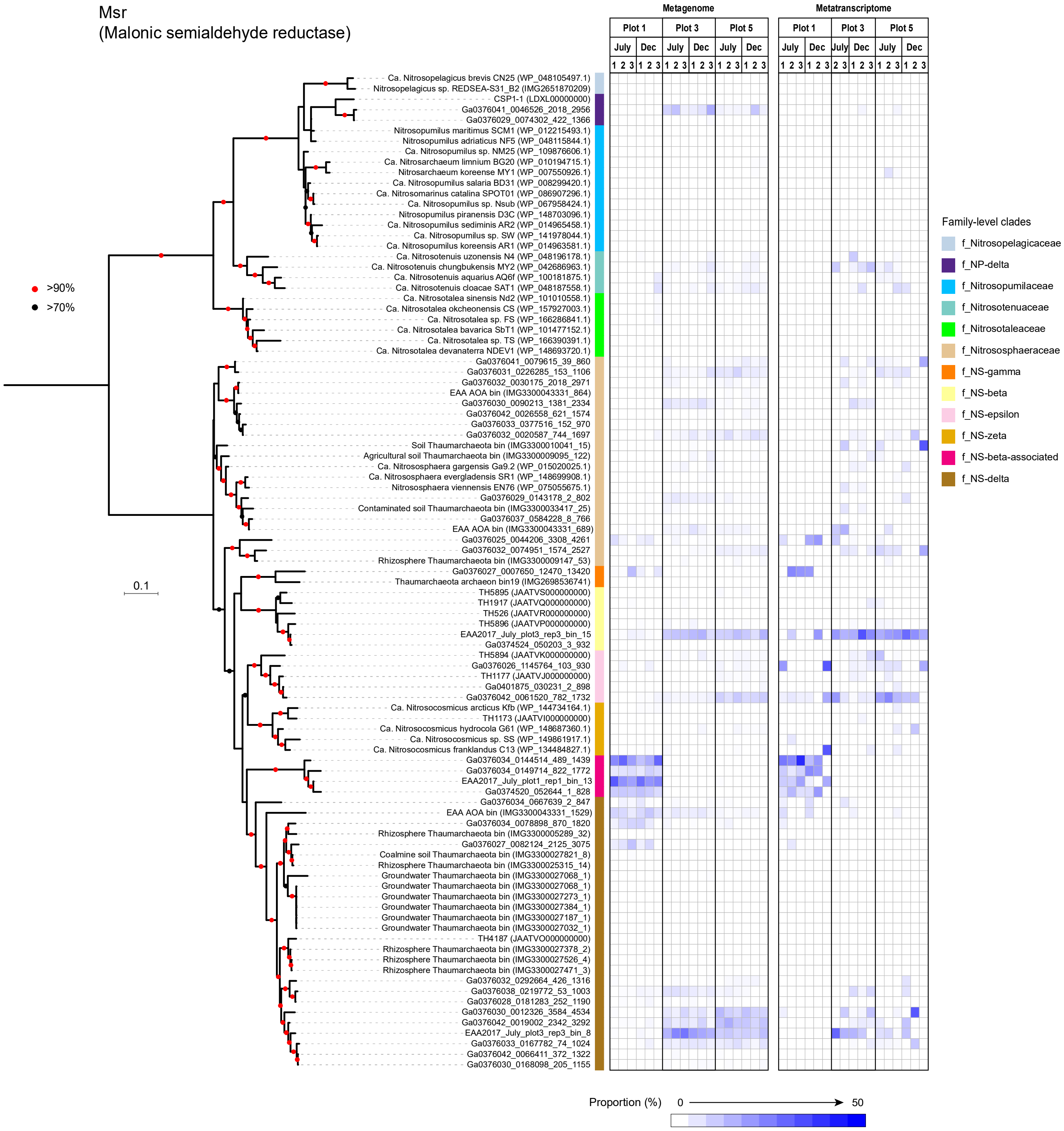

Msr
(Malonic semialdehyde reductase)
Metagenome
Metatranscriptome
Plot 1
Plot 3
Plot 5
July
Dec
1 2 3
>90%
>70%
0.1
Ca. Nitrosopelagicus brevis CN25 (WP_048105497.1)
Nitrosopelagicus sp. REDSEA-S31_B2 (IMG2651870209)
CSP1-1 (LDXL00000000)
Ga0376041_0046526_2018_2956
Ga0376029_0074302_422_1366
Nitrosopumilus maritimus SCM1 (WP_012215493.1)
Nitrosopumilus adriaticus NF5 (WP_048115844.1)
Ca. Nitrosopumilus sp. NM25 (WP_109876606.1)
Ca. Nitrosarchaeum limnium BG20 (WP_010194715.1)
Nitrosarchaeum koreense MY1 (WP_007550926.1)
Ca. Nitrosopumilus salaria BD31 (WP_008299420.1)
Ca. Nitrosomarinus catalina SPOT01 (WP_086907296.1)
Ca. Nitrosopumilus sp. Nsub (WP_067958424.1)
Nitrosopumilus piranensis D3C (WP_148703096.1)
Ca. Nitrosopumilus sediminis AR2 (WP_014965458.1)
Ca. Nitrosopumilus sp. SW (WP_141978044.1)
Ca. Nitrosopumilus koreensis AR1 (WP_014963581.1)
Ca. Nitrosotenuis uzonensis N4 (WP_048196178.1)
Ca. Nitrosotenuis chungbukensis MY2 (WP_042686963.1)
Ca. Nitrosotenuis aquarius AQ6f (WP_100181875.1)
Ca. Nitrosotenuis cloacae SAT1 (WP_048187558.1)
Ca. Nitrosotalea sinensis Nd2 (WP_101010558.1)
Ca. Nitrosotalea okcheonensis CS (WP_157927003.1)
Ca. Nitrosotalea sp. FS (WP_166286841.1)
Ca. Nitrosotalea bavarica SbT1 (WP_101477152.1)
Ca. Nitrosotalea sp. TS (WP_166390391.1)
Ca. Nitrosotalea devanaterra NDEV1 (WP_148693720.1)
Ga0376041_0079615_39_860
Ga0376031_0226285_153_1106
Ga0376032_0030175_2018_2971
EAA AOA bin (IMG3300043331_864)
Ga0376030_0090213_1381_2334
Ga0376042_0026558_621_1574
Ga0376033_0377516_152_970
Ga0376032_0020587_744_1697
Soil Thaumarchaeota bin (IMG3300010041_15)
Agricultural soil Thaumarchaeota bin (IMG3300009095_122)
Ca. Nitrososphaera gargensis Ga9.2 (WP_015020025.1)
Ca. Nitrososphaera evergladensis SR1 (WP_148699908.1)
Nitrososphaera viennensis EN76 (WP_075055675.1)
Ga0376029_0143178_2_802
Contaminated soil Thaumarchaeota bin (IMG3300033417_25)
Ga0376037_0584228_8_766
EAA AOA bin (IMG3300043331_689)
Ga0376025_0044206_3308_4261
Ga0376032_0074951_1574_2527
Rhizosphere Thaumarchaeota bin (IMG3300009147_53)
Ga0376027_0007650_12470_13420
Thaumarchaeota archaeon bin19 (IMG2698536741)
TH5895 (JAATVS000000000)
TH1917 (JAATVQ000000000)
TH526 (JAATVR000000000)
TH5896 (JAATVP000000000)
EAA2017_July_plot3_rep3_bin_15
Ga0374524_050203_3_932
TH5894 (JAATVK000000000)
Ga0376026_1145764_103_930
TH1177 (JAATVJ000000000)
Ga0401875_030231_2_898
Ga0376042_0061520_782_1732
Ca. Nitrosocosmicus arcticus Kfb (WP_144734164.1)
TH1173 (JAATVI000000000)
Ca. Nitrosocosmicus hydrocola G61 (WP_148687360.1)
Ca. Nitrosocosmicus sp. SS (WP_149861917.1)
Ca. Nitrosocosmicus franklandus C13 (WP_134484827.1)
Ga0376034_0144514_489_1439
Ga0376034_0149714_822_1772
EAA2017_July_plot1_rep1_bin_13
Ga0374520_052644_1_828
Ga0376034_0667639_2_847
EAA AOA bin (IMG3300043331_1529)
Ga0376034_0078898_870_1820
Rhizosphere Thaumarchaeota bin (IMG3300005289_32)
Ga0376027_0082124_2125_3075
Coalmine soil Thaumarchaeota bin (IMG3300027821_8)
Rhizosphere Thaumarchaeota bin (IMG3300025315_14)
Groundwater Thaumarchaeota bin (IMG3300027068_1)
Groundwater Thaumarchaeota bin (IMG3300027068_1)
Groundwater Thaumarchaeota bin (IMG3300027273_1)
Groundwater Thaumarchaeota bin (IMG3300027384_1)
Groundwater Thaumarchaeota bin (IMG3300027187_1)
Groundwater Thaumarchaeota bin (IMG3300027032_1)
TH4187 (JAATVO000000000)
Rhizosphere Thaumarchaeota bin (IMG3300027378_2)
Rhizosphere Thaumarchaeota bin (IMG3300027526_4)
Rhizosphere Thaumarchaeota bin (IMG3300027471_3)
Ga0376032_0292664_426_1316
Ga0376038_0219772_53_1003
Ga0376028_0181283_252_1190
Ga0376030_0012326_3584_4534
Ga0376042_0019002_2342_3292
EAA2017_July_plot3_rep3_bin_8
Ga0376033_0167782_74_1024
Ga0376042_0066411_372_1322
Ga0376030_0168098_205_1155
Family-level clades
f_Nitrosopelagicaceae
f_NP-delta
f_Nitrosopumilaceae
f_Nitrosotenuaceae
f_Nitrosotaleaceae
f_Nitrososphaeraceae
f_NS-gamma
f_NS-beta
f_NS-epsilon
f_NS-zeta
f_NS-beta-associated
f_NS-delta
Proportion (%)
0
50

Fig. S12. Maximum likelihood phylogeny of AOA Msr protein sequences recovered from metagenomes and metatranscriptomes in this study (left) and heat map of relative abundance based on read mapping (right). Representative sequences assembled in this study are indicated with their IMG accession number ("Ga*"). Reference sequences of cultured AOA genomes and MAGs retrieved from NCBI RefSeq and IMG databases were used to classify each phylotype to a family-level AOA lineage based on genome-wide taxonomic ranking system (Sheridan et al 2020). Ultrafast bootstrap values >70% and >90% (1,000 replicates) are indicated by black and red solid circles, respectively. The scale bar represents 0.1 substitution per amino acid position. Heat map shows the proportion of mapped sequencing reads of each phylotype relative to total AOA *msr* reads in each metagenome or metatranscriptome. Shown are all three biological replicates, except for plot 3, for which only two metatranscriptomes were recovered in July 2017.

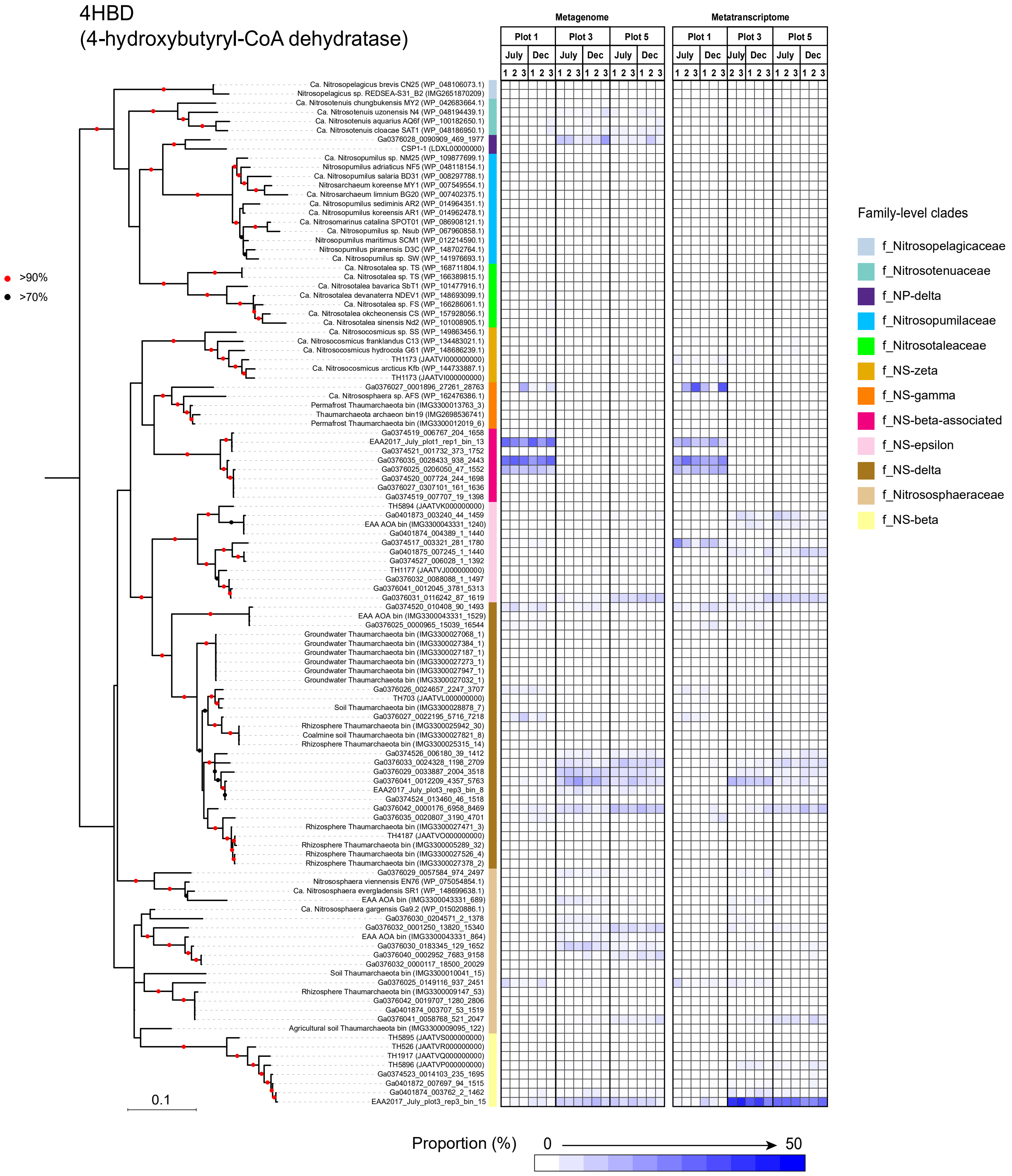

4HBD
(4-hydroxybutyryl-CoA dehydratase)
Metagenome
Metatranscriptome
Plot 1
Plot 3
Plot 5
July
Dec
1 2 3
>90%
>70%
Ca. Nitrosopelagicus brevis CN25 (WP_048106073.1)
Nitrosopelagicus sp. REDSEA-S31_B2 (IMG2651870209)
Ca. Nitrosotenuis chungbukensis MY2 (WP_042683664.1)
Ca. Nitrosotenuis uzonensis N4 (WP_048194439.1)
Ca. Nitrosotenuis aquarius AQ6f (WP_100182650.1)
Ca. Nitrosotenuis cloacae SAT1 (WP_048186950.1)
Ga0376028_0090909_469_1977
CSP1-1 (LDXL00000000)
Ca. Nitrosopumilus sp. NM25 (WP_109877699.1)
Nitrosopumilus adriaticus NF5 (WP_048118154.1)
Ca. Nitrosopumilus salaria BD31 (WP_008297788.1)
Nitrosarchaeum koreense MY1 (WP_007549554.1)
Ca. Nitrosarchaeum limnium BG20 (WP_007402375.1)
Ca. Nitrosopumilus sediminis AR2 (WP_014964351.1)
Ca. Nitrosopumilus koreensis AR1 (WP_014962478.1)
Ca. Nitrosomarinus catalina SPOT01 (WP_086908121.1)
Ca. Nitrosopumilus sp. Nsub (WP_067960858.1)
Nitrosopumilus maritimus SCM1 (WP_012214590.1)
Nitrosopumilus piranensis D3C (WP_148702764.1)
Ca. Nitrosopumilus sp. SW (WP_141976693.1)
Ca. Nitrosotalea sp. TS (WP_168711804.1)
Ca. Nitrosotalea sp. TS (WP_166389815.1)
Ca. Nitrosotalea bavarica SbT1 (WP_101477916.1)
Ca. Nitrosotalea devanaterra NDEV1 (WP_148693099.1)
Ca. Nitrosotalea sp. FS (WP_166286061.1)
Ca. Nitrosotalea okcheonensis CS (WP_157928056.1)
Ca. Nitrosotalea sinensis Nd2 (WP_101008905.1)
Ca. Nitrosocosmicus sp. SS (WP_149863456.1)
Ca. Nitrosocosmicus franklandus C13 (WP_134483021.1)
Ca. Nitrosocosmicus hydrocola G61 (WP_148686239.1)
TH1173 (JAATVI000000000)
Ca. Nitrosocosmicus arcticus Kfb (WP_144733887.1)
TH1173 (JAATVI000000000)
Ga0376027_0001896_27261_28763
Ca. Nitrososphaera sp. AFS (WP_162476386.1)
Permafrost Thaumarchaeota bin (IMG3300013763_3)
Thaumarchaeota archaeon bin19 (IMG2698536741)
Permafrost Thaumarchaeota bin (IMG3300012019_6)
Ga0374519_006767_204_1658
EAA2017_July_plot1_rep1_bin_13
Ga0374521_001732_373_1752
Ga0376035_0028433_938_2443
Ga0376025_0206050_47_1552
Ga0374520_007724_244_1698
Ga0376027_0307101_161_1636
Ga0374519_007707_19_1398
TH5894 (JAATVK000000000)
Ga0401873_003240_44_1459
EAA AOA bin (IMG3300043331_1240)
Ga0401874_004389_1_1440
Ga0374517_003321_281_1780
Ga0401875_007245_1_1440
Ga0374527_006028_1_1392
TH1177 (JAATVJ000000000)
Ga0376032_0088088_1_1497
Ga0376041_0012045_3781_5313
Ga0376031_0116242_87_1619
Ga0374520_010408_90_1493
EAA AOA bin (IMG3300043331_1529)
Ga0376025_0000965_15039_16544
Groundwater Thaumarchaeota bin (IMG3300027068_1)
Groundwater Thaumarchaeota bin (IMG3300027384_1)
Groundwater Thaumarchaeota bin (IMG3300027187_1)
Groundwater Thaumarchaeota bin (IMG3300027273_1)
Groundwater Thaumarchaeota bin (IMG3300027947_1)
Groundwater Thaumarchaeota bin (IMG3300027032_1)
Ga0376026_0024657_2247_3707
TH703 (JAATVL000000000)
Soil Thaumarchaeota bin (IMG3300028878_7)
Ga0376027_0022195_5716_7218
Rhizosphere Thaumarchaeota bin (IMG3300025942_30)
Coalmine soil Thaumarchaeota bin (IMG3300027821_8)
Rhizosphere Thaumarchaeota bin (IMG3300025315_14)
Ga0374526_006180_39_1412
Ga0376033_0024328_1198_2709
Ga0376029_0033887_2004_3518
Ga0376041_0012209_4357_5763
EAA2017_July_plot3_rep3_bin_8
Ga0374524_013460_46_1518
Ga0376042_0000176_6958_8469
Ga0376035_0020807_3190_4701
Rhizosphere Thaumarchaeota bin (IMG3300027471_3)
TH4187 (JAATVO000000000)
Rhizosphere Thaumarchaeota bin (IMG3300005289_32)
Rhizosphere Thaumarchaeota bin (IMG3300027526_4)
Rhizosphere Thaumarchaeota bin (IMG3300027378_2)
Ga0376029_0057584_974_2497
Nitrososphaera viennensis EN76 (WP_075054854.1)
Ca. Nitrososphaera evergladensis SR1 (WP_148699638.1)
EAA AOA bin (IMG3300043331_689)
Ca. Nitrososphaera gargensis Ga9.2 (WP_015020886.1)
Ga0376030_0204571_2_1378
Ga0376032_0001250_13820_15340
EAA AOA bin (IMG3300043331_864)
Ga0376030_0183345_129_1652
Ga0376040_0002952_7683_9158
Ga0376032_0000117_18500_20029
Soil Thaumarchaeota bin (IMG3300010041_15)
Ga0376025_0149116_937_2451
Rhizosphere Thaumarchaeota bin (IMG3300009147_53)
Ga0376042_0019707_1280_2806
Ga0401874_003707_53_1519
Ga0376041_0058768_521_2047
Agricultural soil Thaumarchaeota bin (IMG3300009095_122)
TH5895 (JAATVS000000000)
TH526 (JAATVR000000000)
TH1917 (JAATVQ000000000)
TH5896 (JAATVP000000000)
Ga0374523_0014103_235_1695
Ga0401872_007697_94_1515
Ga0401874_003762_2_1462
EAA2017_July_plot3_rep3_bin_15
0.1
Family-level clades
f_Nitrosopelagicaceae
f_Nitrosotenuaceae
f_NP-delta
f_Nitrosopumilaceae
f_Nitrosotaleaceae
f_NS-zeta
f_NS-gamma
f_NS-beta-associated
f_NS-epsilon
f_NS-delta
f_Nitrososphaeraceae
f_NS-beta
Proportion (%)
0
50

Fig. S13. Maximum likelihood phylogeny of AOA 4HBD protein sequences recovered from metagenomes and metatranscriptomes in this study (left) and heat map of relative abundance based on read mapping (right). Representative sequences assembled in this study are indicated with their IMG accession number (“Ga*”). Reference sequences of cultured AOA genomes and MAGs retrieved from NCBI RefSeq and IMG databases were used to classify each phylotype to a family-level AOA lineage based on genome-wide taxonomic ranking system (Sheridan et al 2020). Ultrafast bootstrap values >70% and >90% (1,000 replicates) are indicated by black and red solid circles, respectively. The scale bar represents 0.1 substitution per amino acid position. Heat map shows the proportion of mapped sequencing reads of each phylotype relative to total AOA *4hbd* reads in each metagenome or metatranscriptome. Shown are all three biological replicates, except for plot 3, for which only two metatranscriptomes were recovered in July 2017.

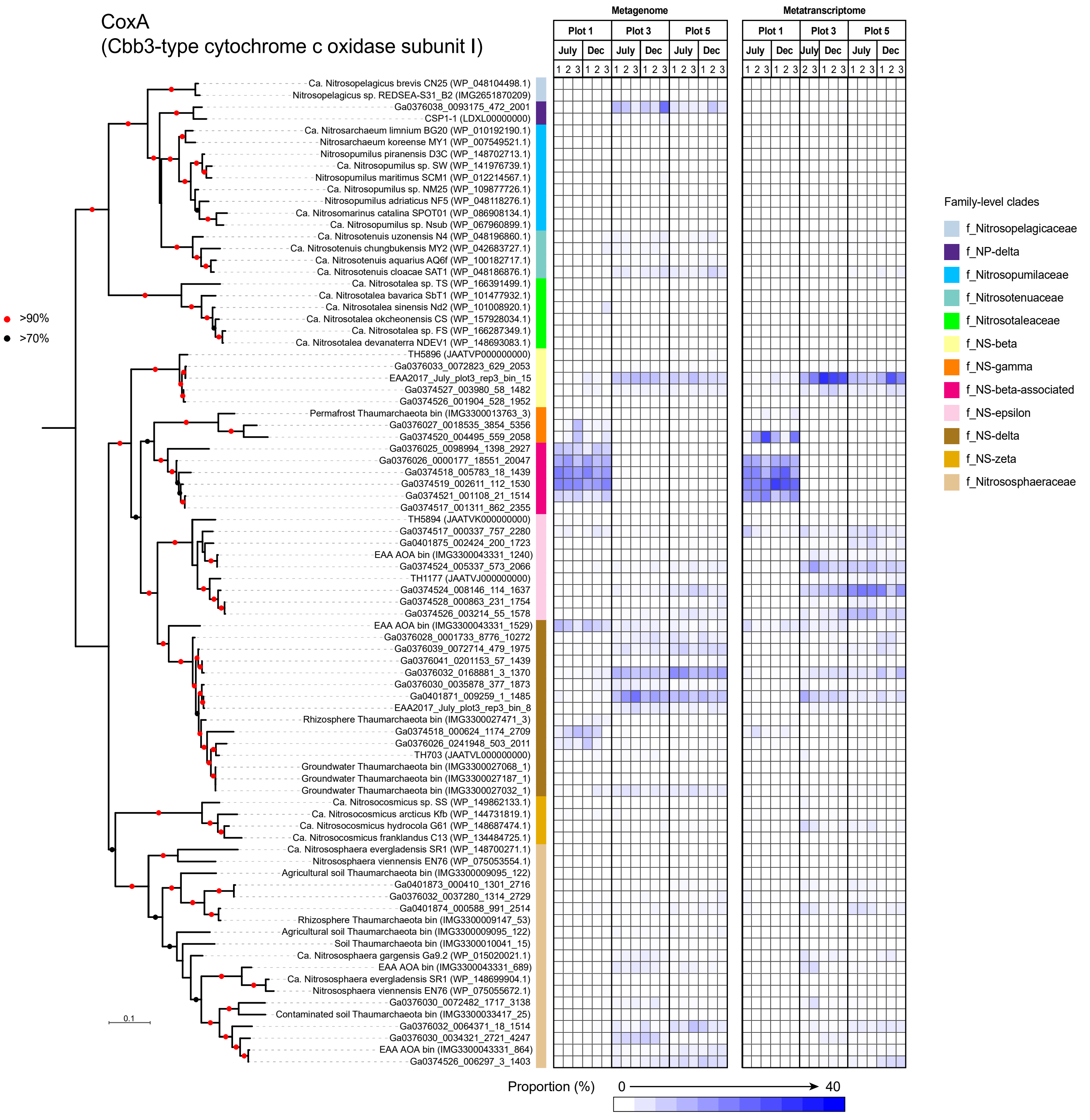

CoxA
(Cbb3-type cytochrome c oxidase subunit I)
Metagenome
Metatranscriptome
Plot 1
Plot 3
Plot 5
July
Dec
>90%
>70%
0.1
Family-level clades
f_Nitrosopelagicaceae
f_NP-delta
f_Nitrosopumilaceae
f_Nitrosotenuaceae
f_Nitrosotaleaceae
f_NS-beta
f_NS-gamma
f_NS-beta-associated
f_NS-epsilon
f_NS-delta
f_NS-zeta
f_Nitrososphaeraceae
Proportion (%)
0
40

Fig. S14. Maximum likelihood phylogeny of AOA CoxA protein sequences recovered from metagenomes and metatranscriptomes in this study (left) and heat map of relative abundance based on read mapping (right). Representative sequences assembled in this study are indicated with their IMG accession number ("Ga*"). Reference sequences of cultured AOA genomes and MAGs retrieved from NCBI RefSeq and IMG databases were used to classify each phylotype to a family-level AOA lineage based on genome-wide taxonomic ranking system (Sheridan et al 2020). Ultrafast bootstrap values >70% and >90% (1,000 replicates) are indicated by black and red solid circles, respectively. The scale bar represents 0.1 substitution per amino acid position. Heat map shows the proportion of mapped sequencing reads of each phylotype relative to total AOA *coxA* reads in each metagenome or metatranscriptome. Shown are all three biological replicates, except for plot 3, for which only two metatranscriptomes were recovered in July 2017.

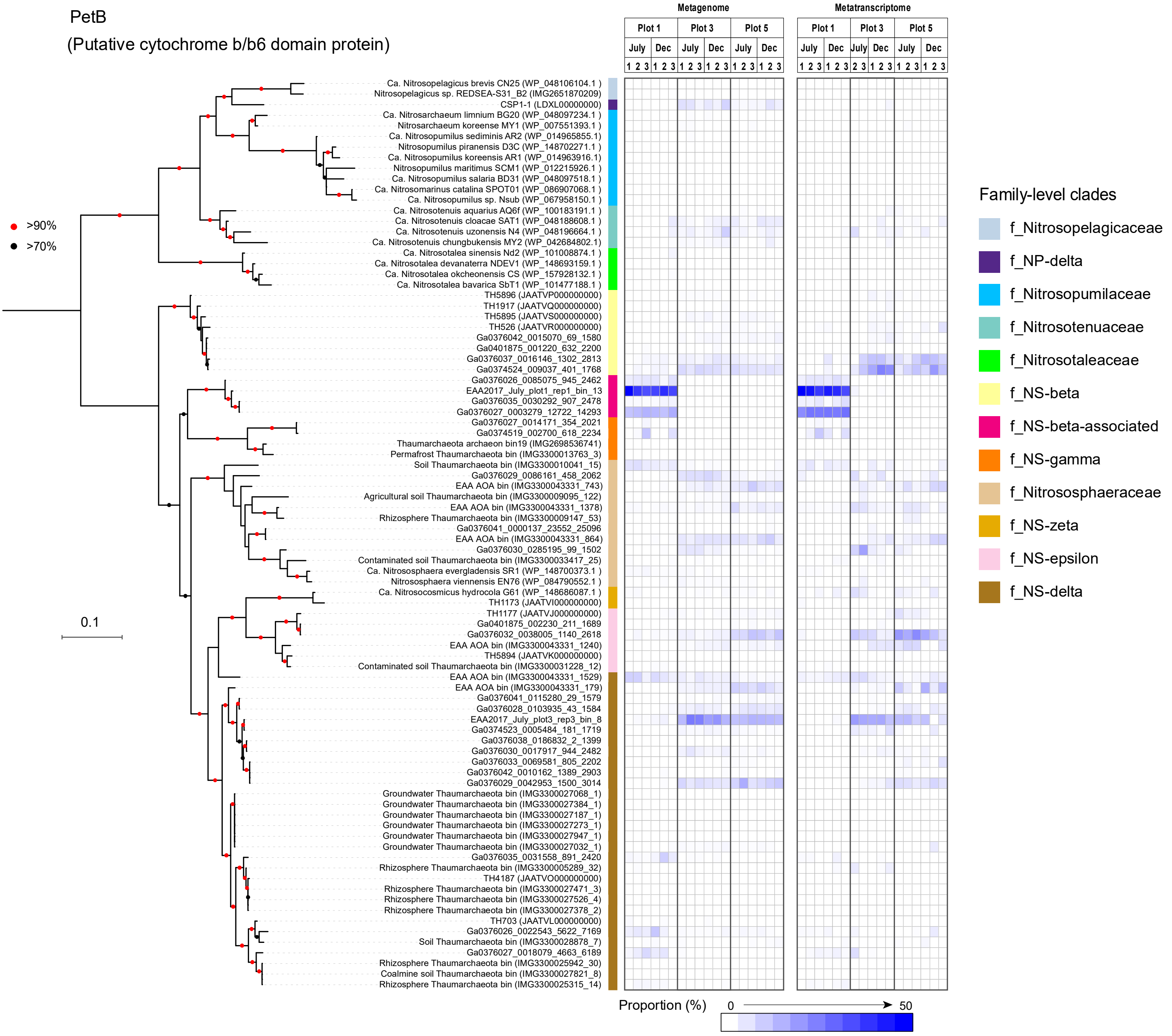

PetB
(Putative cytochrome b/b6 domain protein)
>90%
>70%
0.1
Metagenome
Metatranscriptome
Plot 1
Plot 3
Plot 5
July
Dec
1 2 3
Ca. Nitrosopelagicus brevis CN25 (WP_048106104.1)
Nitrosopelagicus sp. REDSEA-S31_B2 (IMG2651870209)
CSP1-1 (LDXL00000000)
Ca. Nitrosarchaeum limnium BG20 (WP_048097234.1)
Nitrosarchaeum koreense MY1 (WP_007551393.1)
Ca. Nitrosopumilus sediminis AR2 (WP_014965855.1)
Nitrosopumilus piranensis D3C (WP_148702271.1)
Ca. Nitrosopumilus koreensis AR1 (WP_014963916.1)
Nitrosopumilus maritimus SCM1 (WP_012215926.1)
Ca. Nitrosopumilus salaria BD31 (WP_048097518.1)
Ca. Nitrosomarinus catalina SPOT01 (WP_086907068.1)
Ca. Nitrosopumilus sp. Nsub (WP_067958150.1)
Ca. Nitrosotenuis aquarius AQ6f (WP_100183191.1)
Ca. Nitrosotenuis cloacae SAT1 (WP_048188608.1)
Ca. Nitrosotenuis uzonensis N4 (WP_048196664.1)
Ca. Nitrosotenuis chungbukensis MY2 (WP_042684802.1)
Ca. Nitrosotalea sinensis Nd2 (WP_101008874.1)
Ca. Nitrosotalea devanaterra NDEV1 (WP_148693159.1)
Ca. Nitrosotalea okcheonensis CS (WP_157928132.1)
Ca. Nitrosotalea bavarica SbT1 (WP_101477188.1)
TH5896 (JAATVP000000000)
TH1917 (JAATVQ000000000)
TH5895 (JAATVS000000000)
TH526 (JAATVR000000000)
Ga0376042_0015070_69_1580
Ga0401875_001220_632_2200
Ga0376037_0016146_1302_2813
Ga0374524_009037_401_1768
Ga0376026_0085075_945_2462
EAA2017_July_plot1_rep1_bin_13
Ga0376035_0030292_907_2478
Ga0376027_0003279_12722_14293
Ga0376027_0014171_354_2021
Ga0374519_002700_618_2234
Thaumarchaeota archaeon bin19 (IMG2698536741)
Permafrost Thaumarchaeota bin (IMG3300013763_3)
Soil Thaumarchaeota bin (IMG3300010041_15)
Ga0376029_0086161_458_2062
EAA AOA bin (IMG3300043331_743)
Agricultural soil Thaumarchaeota bin (IMG3300009095_122)
EAA AOA bin (IMG3300043331_1378)
Rhizosphere Thaumarchaeota bin (IMG3300009147_53)
Ga0376041_0000137_23552_25096
EAA AOA bin (IMG3300043331_864)
Ga0376030_0285195_99_1502
Contaminated soil Thaumarchaeota bin (IMG3300033417_25)
Ca. Nitrososphaera evergladensis SR1 (WP_148700373.1)
Nitrososphaera viennensis EN76 (WP_084790552.1)
Ca. Nitrosocosmicus hydrocola G61 (WP_148686087.1)
TH1173 (JAATVI000000000)
TH1177 (JAATVJ000000000)
Ga0401875_002230_211_1689
Ga0376032_0038005_1140_2618
EAA AOA bin (IMG3300043331_1240)
TH5894 (JAATVK000000000)
Contaminated soil Thaumarchaeota bin (IMG3300031228_12)
EAA AOA bin (IMG3300043331_1529)
EAA AOA bin (IMG3300043331_179)
Ga0376041_0115280_29_1579
Ga0376028_0103935_43_1584
EAA2017_July_plot3_rep3_bin_8
Ga0374523_0005484_181_1719
Ga0376038_0186832_2_1399
Ga0376030_0017917_944_2482
Ga0376033_0069581_805_2202
Ga0376042_0010162_1389_2903
Ga0376029_0042953_1500_3014
Groundwater Thaumarchaeota bin (IMG3300027068_1)
Groundwater Thaumarchaeota bin (IMG3300027384_1)
Groundwater Thaumarchaeota bin (IMG3300027187_1)
Groundwater Thaumarchaeota bin (IMG3300027273_1)
Groundwater Thaumarchaeota bin (IMG3300027947_1)
Groundwater Thaumarchaeota bin (IMG3300027032_1)
Ga0376035_0031558_891_2420
Rhizosphere Thaumarchaeota bin (IMG3300005289_32)
TH4187 (JAATVO000000000)
Rhizosphere Thaumarchaeota bin (IMG3300027471_3)
Rhizosphere Thaumarchaeota bin (IMG3300027526_4)
Rhizosphere Thaumarchaeota bin (IMG3300027378_2)
TH703 (JAATVL000000000)
Ga0376026_0022543_5622_7169
Soil Thaumarchaeota bin (IMG3300028878_7)
Ga0376027_0018079_4663_6189
Rhizosphere Thaumarchaeota bin (IMG3300025942_30)
Coalmine soil Thaumarchaeota bin (IMG3300027821_8)
Rhizosphere Thaumarchaeota bin (IMG3300025315_14)
Proportion (%)
0
50
Family-level clades
f_Nitrosopelagicaceae
f_NP-delta
f_Nitrosopumilaceae
f_Nitrosotenuaceae
f_Nitrosotaleaceae
f_NS-beta
f_NS-beta-associated
f_NS-gamma
f_Nitrososphaeraceae
f_NS-zeta
f_NS-epsilon
f_NS-delta

Fig. S15. Maximum likelihood phylogeny of AOA PetB protein sequences recovered from metagenomes and metatranscriptomes in this study (left) and heat map of relative abundance based on read mapping (right). Representative sequences assembled in this study are indicated with their IMG accession number (“Ga*”). Reference sequences of cultured AOA genomes and MAGs retrieved from NCBI RefSeq and IMG databases were used to classify each phylotype to a family-level AOA lineage based on genome-wide taxonomic ranking system (Sheridan et al 2020). Ultrafast bootstrap values >70% and >90% (1,000 replicates) are indicated by black and red solid circles, respectively. The scale bar represents 0.1 substitution per amino acid position. Heat map shows the proportion of mapped sequencing reads of each phylotype relative to total AOA *petB* reads in each metagenome or metatranscriptome. Shown are all three biological replicates, except for plot 3, for which only two metatranscriptomes were recovered in July 2017.

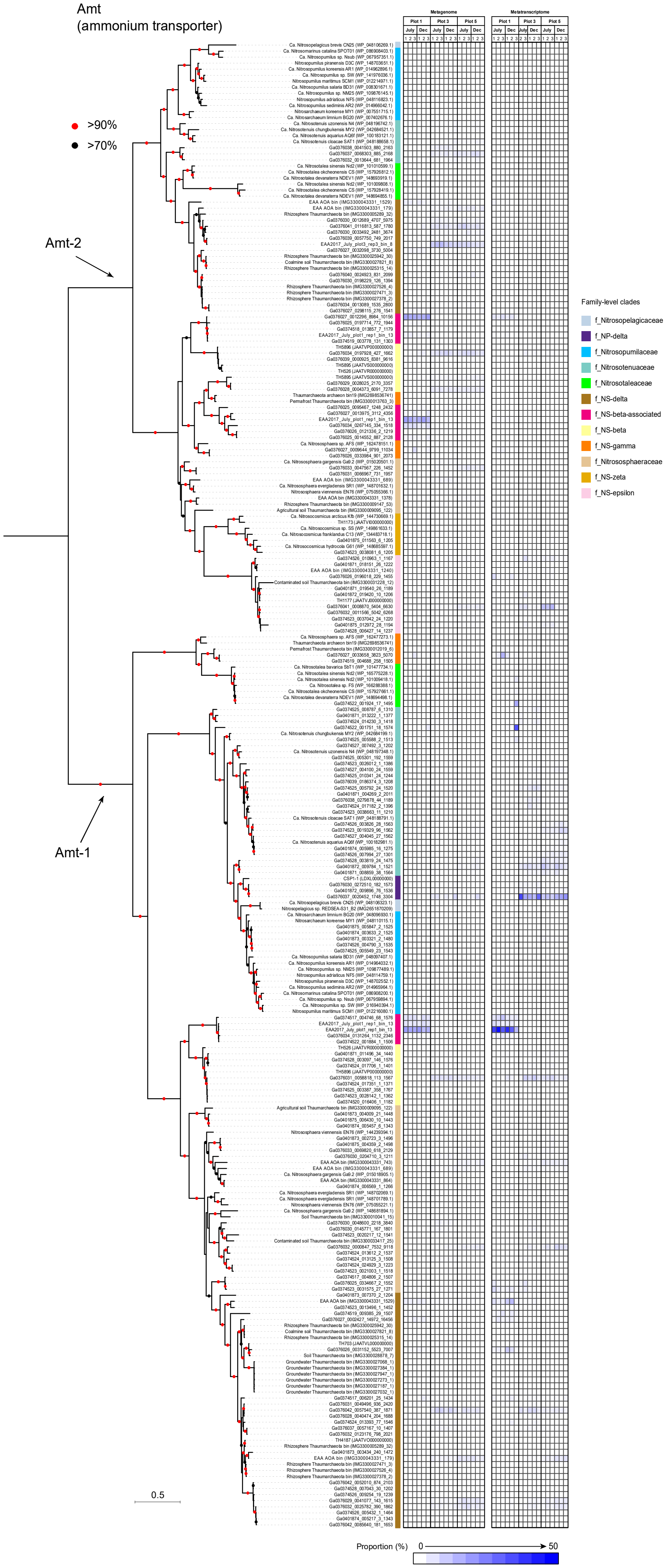
Amt
(ammonium transporter)
>90%
>70%
Amt-2
Amt-1
0.5
Metagenome
Metatranscriptome
Plot 1
Plot 3
Plot 5
July
Dec
Family-level clades
f_Nitrosopelagicaceae
f_NP-delta
f_Nitrosopumilaceae
f_Nitrosotenuaceae
f_Nitrosotaleaceae
f_NS-delta
f_NS-beta-associated
f_NS-beta
f_NS-gamma
f_Nitrososphaeraceae
f_NS-zeta
f_NS-epsilon
Proportion (%)
0
50

S38

Fig. S16. Maximum likelihood phylogeny of AOA Amt protein sequences recovered from metagenomes and metatranscriptomes in this study (left) and heat map of relative abundance based on read mapping (right). Representative sequences assembled in this study are indicated with their IMG accession number ("Ga*"). Reference sequences of cultured AOA genomes and MAGs retrieved from NCBI RefSeq and IMG databases were used to classify each phylotype to a family-level AOA lineage based on genome-wide taxonomic ranking system (Sheridan et al 2020). Ultrafast bootstrap values >70% and >90% (1,000 replicates) are indicated by black and red solid circles, respectively. The scale bar represents 0.5 substitution per amino acid position. Heat map shows the proportion of mapped sequencing reads of each phylotype relative to total AOA *amt* reads in each metagenome or metatranscriptome. Shown are all three biological replicates, except for plot 3, for which only two metatranscriptomes were recovered in July 2017.

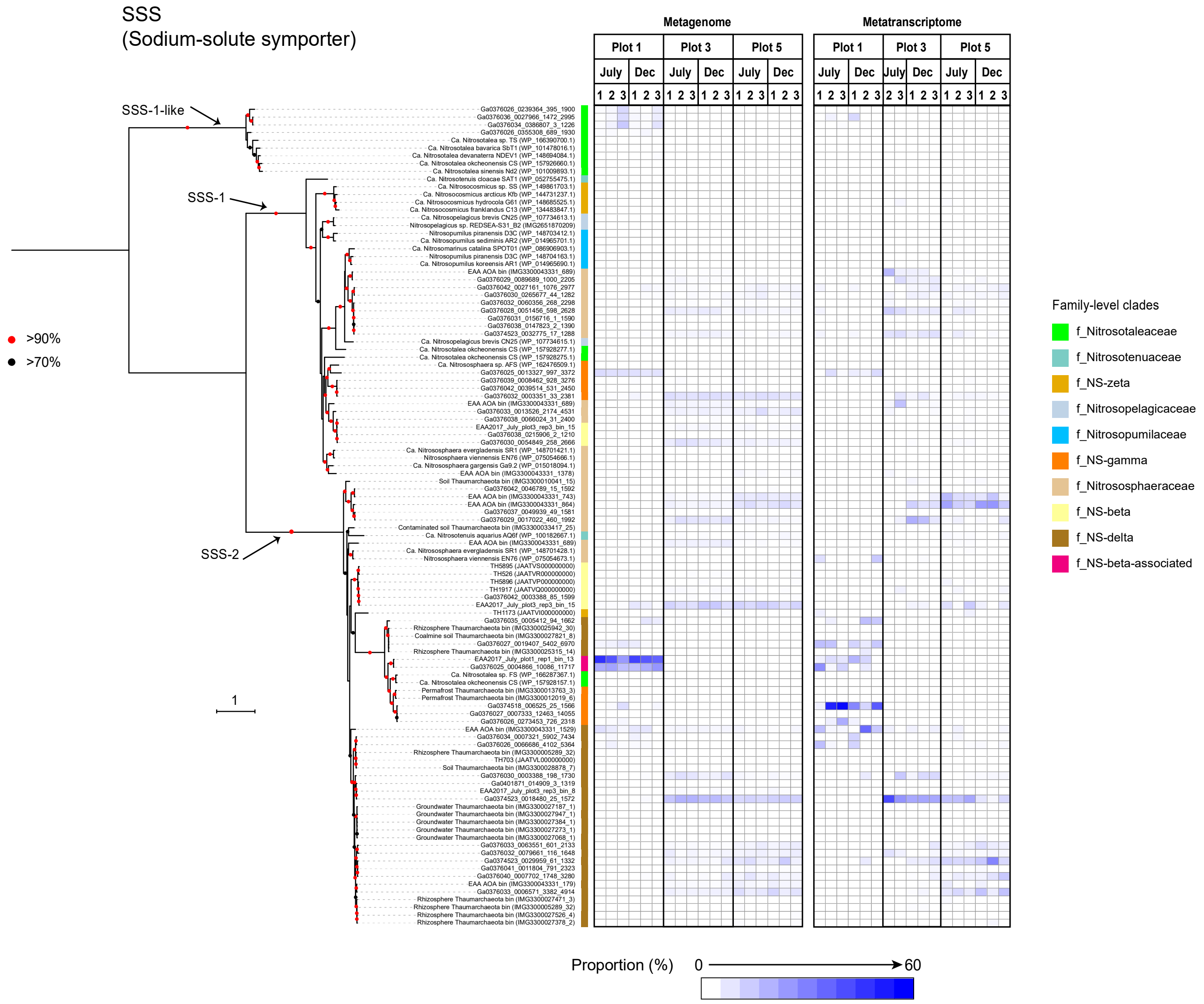

SSS
(Sodium-solute symporter)
SSS-1-like
SSS-1
SSS-2
>90%
>70%
1
Metagenome
Metatranscriptome
Plot 1
Plot 3
Plot 5
July
Dec
Family-level clades
f_Nitrosotaleaceae
f_Nitrosotenuaceae
f_NS-zeta
f_Nitrosopelagicaceae
f_Nitrosopumilaceae
f_NS-gamma
f_Nitrososphaeraceae
f_NS-beta
f_NS-delta
f_NS-beta-associated
Proportion (%)
0
60

Fig. S17. Maximum likelihood phylogeny of AOA SSS protein sequences recovered from metagenomes and metatranscriptomes in this study (left) and heat map of relative abundance based on read mapping (right). Representative sequences assembled in this study are indicated with their IMG accession number ("Ga*"). Reference sequences of cultured AOA genomes and MAGs retrieved from NCBI RefSeq and IMG databases were used to classify each phylotype to a family-level AOA lineage based on genome-wide taxonomic ranking system (Sheridan et al 2020). Ultrafast bootstrap values >70% and >90% (1,000 replicates) are indicated by black and red solid circles, respectively. The scale bar represents 1 substitution per amino acid position. Heat map shows the proportion of mapped sequencing reads of each phylotype relative to total AOA *sss* reads in each metagenome or metatranscriptome. Shown are all three biological replicates, except for plot 3, for which only two metatranscriptomes were recovered in July 2017.

Ut (urea transporter)

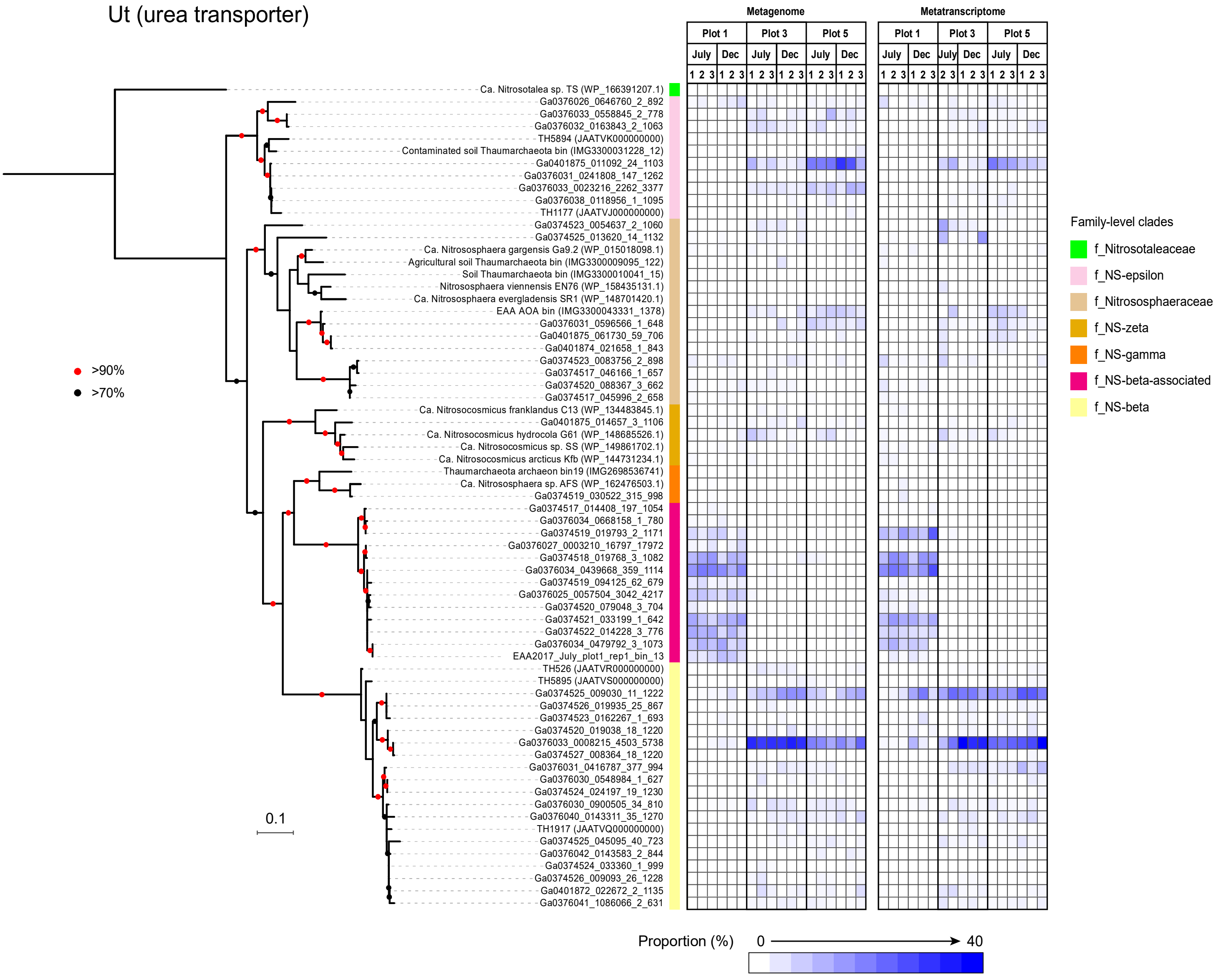

Fig. S18. Maximum likelihood phylogeny of AOA Ut protein sequences recovered from metagenomes and metatranscriptomes in this study (left) and heat map of relative abundance based on read mapping (right). Representative sequences assembled in this study are indicated with their IMG accession number ("Ga*"). Reference sequences of cultured AOA genomes and MAGs retrieved from NCBI RefSeq and IMG databases were used to classify each phylotype to a family-level AOA lineage based on genome-wide taxonomic ranking system (Sheridan et al 2020). Ultrafast bootstrap values >70% and >90% (1,000 replicates) are indicated by black and red solid circles, respectively. The scale bar represents 0.1 substitution per amino acid position. Heat map shows the proportion of mapped sequencing reads of each phylotype relative to total AOA *ut* reads in each metagenome or metatranscriptome. Shown are all three biological replicates, except for plot 3, for which only two metatranscriptomes were recovered in July 2017.

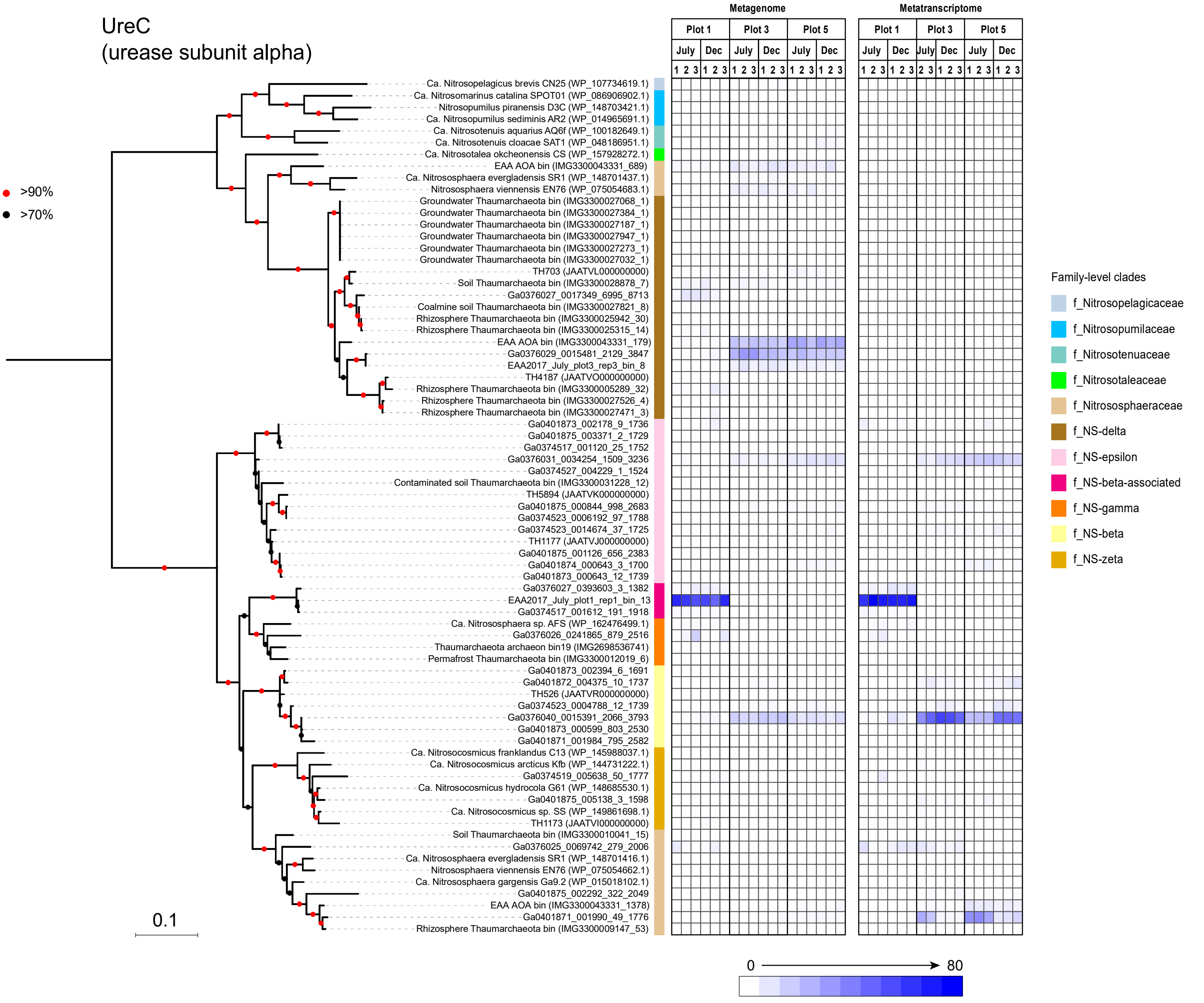
UreC
(urease subunit alpha)
>90%
>70%
0.1
Metagenome
Metatranscriptome
Plot 1
Plot 3
Plot 5
July
Dec
1 2 3
0
80
Family-level clades
f_Nitrosopelagicaceae
f_Nitrosopumilaceae
f_Nitrosotenuaceae
f_Nitrosotaleaceae
f_Nitrososphaeraceae
f_NS-delta
f_NS-epsilon
f_NS-beta-associated
f_NS-gamma
f_NS-beta
f_NS-zeta
Ca. Nitrosopelagicus brevis CN25 (WP_107734619.1)
Ca. Nitrosomarinus catalina SPOT01 (WP_086906902.1)
Nitrosopumilus piranensis D3C (WP_148703421.1)
Ca. Nitrosopumilus sediminis AR2 (WP_014965691.1)
Ca. Nitrosotenuis aquarius AQ6f (WP_100182649.1)
Ca. Nitrosotenuis cloacae SAT1 (WP_048186951.1)
Ca. Nitrosotalea okcheonensis CS (WP_157928272.1)
EAA AOA bin (IMG3300043331_689)
Ca. Nitrososphaera evergladensis SR1 (WP_148701437.1)
Nitrososphaera viennensis EN76 (WP_075054683.1)
Groundwater Thaumarchaeota bin (IMG3300027068_1)
Groundwater Thaumarchaeota bin (IMG3300027384_1)
Groundwater Thaumarchaeota bin (IMG3300027187_1)
Groundwater Thaumarchaeota bin (IMG3300027947_1)
Groundwater Thaumarchaeota bin (IMG3300027273_1)
Groundwater Thaumarchaeota bin (IMG3300027032_1)
TH703 (JAATVL000000000)
Soil Thaumarchaeota bin (IMG3300028878_7)
Ga0376027_0017349_6995_8713
Coalmine soil Thaumarchaeota bin (IMG3300027821_8)
Rhizosphere Thaumarchaeota bin (IMG3300025942_30)
Rhizosphere Thaumarchaeota bin (IMG3300025315_14)
EAA AOA bin (IMG3300043331_179)
Ga0376029_0015481_2129_3847
EAA2017_July_plot3_rep3_bin_8
TH4187 (JAATVO000000000)
Rhizosphere Thaumarchaeota bin (IMG3300005289_32)
Rhizosphere Thaumarchaeota bin (IMG3300027526_4)
Rhizosphere Thaumarchaeota bin (IMG3300027471_3)
Ga0401873_002178_9_1736
Ga0401875_003371_2_1729
Ga0374517_001120_25_1752
Ga0376031_0034254_1509_3236
Ga0374527_004229_1_1524
Contaminated soil Thaumarchaeota bin (IMG3300031228_12)
TH5894 (JAATVK000000000)
Ga0401875_000844_998_2683
Ga0374523_0006192_97_1788
Ga0374523_0014674_37_1725
TH1177 (JAATVJ000000000)
Ga0401875_001126_656_2383
Ga0401874_000643_3_1700
Ga0401873_000643_12_1739
Ga0376027_0393603_3_1382
EAA2017_July_plot1_rep1_bin_13
Ga0374517_001612_191_1918
Ca. Nitrososphaera sp. AFS (WP_162476499.1)
Ga0376026_0241865_879_2516
Thaumarchaeota archaeon bin19 (IMG2698536741)
Permafrost Thaumarchaeota bin (IMG3300012019_6)
Ga0401873_002394_6_1691
Ga0401872_004375_10_1737
TH526 (JAATVR000000000)
Ga0374523_0004788_12_1739
Ga0376040_0015391_2066_3793
Ga0401873_000599_803_2530
Ga0401871_001984_795_2582
Ca. Nitrosocosmicus franklandus C13 (WP_145988037.1)
Ca. Nitrosocosmicus arcticus Kfb (WP_144731222.1)
Ga0374519_005638_50_1777
Ca. Nitrosocosmicus hydrocola G61 (WP_148685530.1)
Ga0401875_005138_3_1598
Ca. Nitrosocosmicus sp. SS (WP_149861698.1)
TH1173 (JAATVI000000000)
Soil Thaumarchaeota bin (IMG3300010041_15)
Ga0376025_0069742_279_2006
Ca. Nitrososphaera evergladensis SR1 (WP_148701416.1)
Nitrososphaera viennensis EN76 (WP_075054662.1)
Ca. Nitrososphaera gargensis Ga9.2 (WP_015018102.1)
Ga0401875_002292_322_2049
EAA AOA bin (IMG3300043331_1378)
Ga0401871_001990_49_1776
Rhizosphere Thaumarchaeota bin (IMG3300009147_53)

Fig. S19. Maximum likelihood phylogeny of AOA UreC protein sequences recovered from metagenomes and metatranscriptomes in this study (left) and heat map of relative abundance based on read mapping (right). Representative sequences assembled in this study are indicated with their IMG accession number ("Ga*"). Reference sequences of cultured AOA genomes and MAGs retrieved from NCBI RefSeq and IMG databases were used to classify each phylotype to a family-level AOA lineage based on genome-wide taxonomic ranking system (Sheridan et al 2020). Ultrafast bootstrap values >70% and >90% (1,000 replicates) are indicated by black and red solid circles, respectively. The scale bar represents 0.1 substitution per amino acid position. Heat map shows the proportion of mapped sequencing reads of each phylotype relative to total AOA *ureC* reads in each metagenome or metatranscriptome. Shown are all three biological replicates, except for plot 3, for which only two metatranscriptomes were recovered in July 2017.

GlnB
(nitrogen regulatory protein P-II)

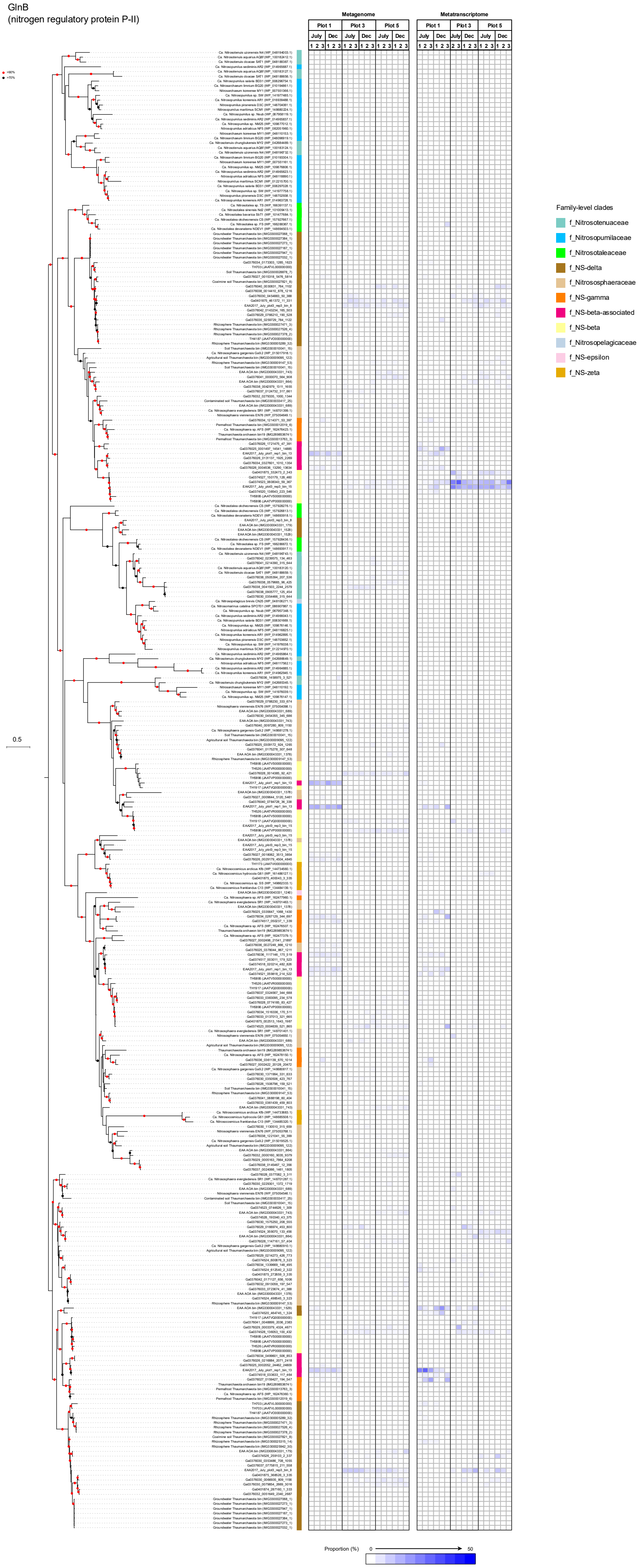

Fig. S20. Maximum likelihood phylogeny of AOA GlnB protein sequences recovered from metagenomes and metatranscriptomes in this study (left) and heat map of relative abundance based on read mapping (right). Representative sequences assembled in this study are indicated with their IMG accession number ("Ga*"). Reference sequences of cultured AOA genomes and MAGs retrieved from NCBI RefSeq and IMG databases were used to classify each phylotype to a family-level AOA lineage based on genome-wide taxonomic ranking system (Sheridan et al 2020). Ultrafast bootstrap values >70% and >90% (1,000 replicates) are indicated by black and red solid circles, respectively. The scale bar represents 0.5 substitution per amino acid position. Heat map shows the proportion of mapped sequencing reads of each phylotype relative to total AOA *glnB* reads in each metagenome or metatranscriptome. Shown are all three biological replicates, except for plot 3, for which only two metatranscriptomes were recovered in July 2017.

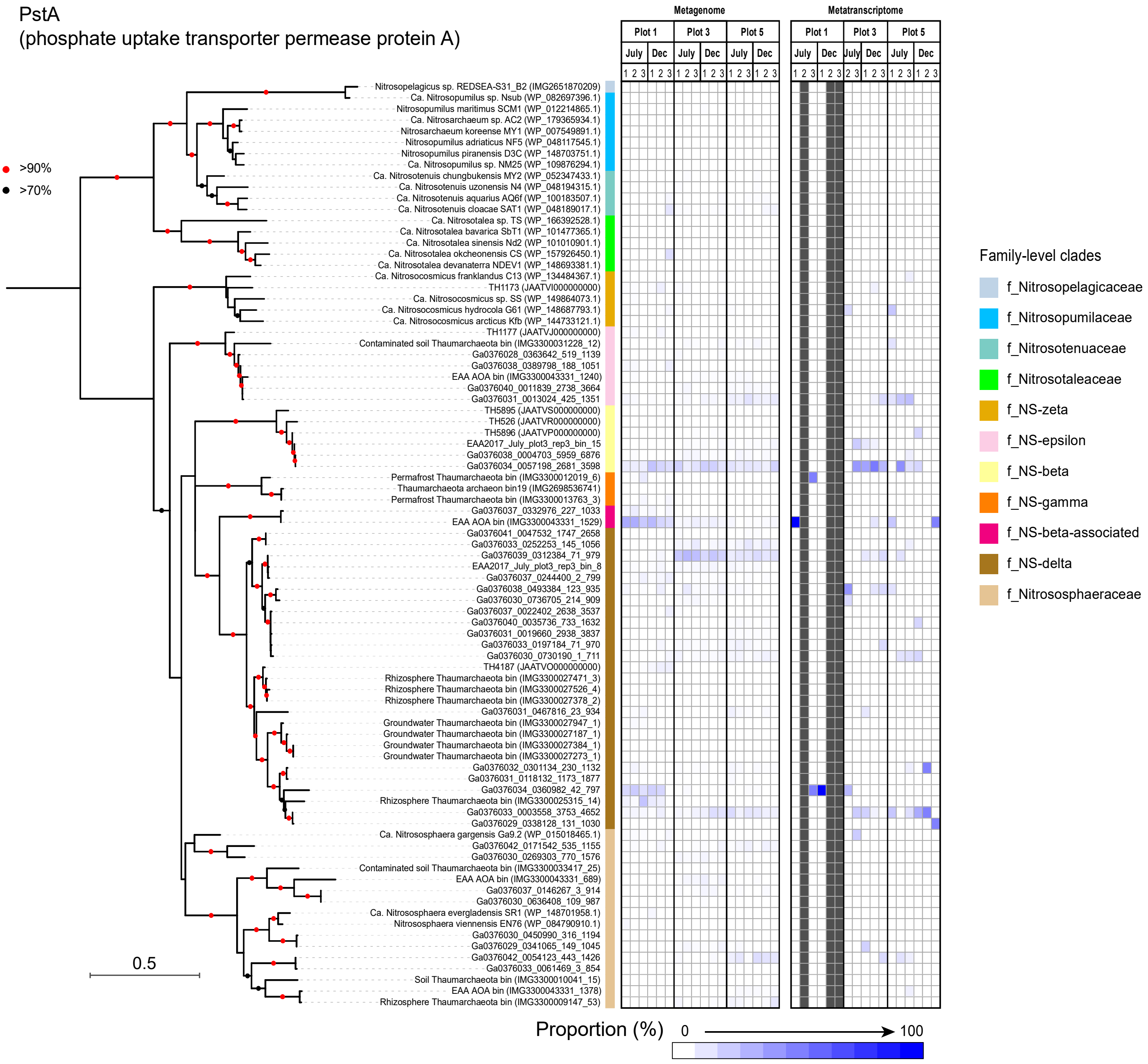
PstA
(phosphate uptake transporter permease protein A)
>90%
>70%
0.5
Metagenome
Metatranscriptome
Plot 1
Plot 3
Plot 5
July
Dec
1 2 3
Nitrosopelagicus sp. REDSEA-S31_B2 (IMG2651870209)
Ca. Nitrosopumilus sp. Nsub (WP_082697396.1)
Nitrosopumilus maritimus SCM1 (WP_012214865.1)
Ca. Nitrosarchaeum sp. AC2 (WP_179365934.1)
Nitrosarchaeum koreense MY1 (WP_007549891.1)
Nitrosopumilus adriaticus NF5 (WP_048117545.1)
Nitrosopumilus piranensis D3C (WP_148703751.1)
Ca. Nitrosopumilus sp. NM25 (WP_109876294.1)
Ca. Nitrosotenuis chungbukensis MY2 (WP_052347433.1)
Ca. Nitrosotenuis uzonensis N4 (WP_048194315.1)
Ca. Nitrosotenuis aquarius AQ6f (WP_100183507.1)
Ca. Nitrosotenuis cloacae SAT1 (WP_048189017.1)
Ca. Nitrosotalea sp. TS (WP_166392528.1)
Ca. Nitrosotalea bavarica SbT1 (WP_101477365.1)
Ca. Nitrosotalea sinensis Nd2 (WP_101010901.1)
Ca. Nitrosotalea okcheonensis CS (WP_157926450.1)
Ca. Nitrosotalea devanaterra NDEV1 (WP_148693381.1)
Ca. Nitrosocosmicus franklandus C13 (WP_134484367.1)
TH1173 (JAATVI000000000)
Ca. Nitrosocosmicus sp. SS (WP_149864073.1)
Ca. Nitrosocosmicus hydrocola G61 (WP_148687793.1)
Ca. Nitrosocosmicus arcticus Kfb (WP_144733121.1)
TH1177 (JAATVJ000000000)
Contaminated soil Thaumarchaeota bin (IMG3300031228_12)
Ga0376028_0363642_519_1139
Ga0376038_0389798_188_1051
EAA AOA bin (IMG3300043331_1240)
Ga0376040_0011839_2738_3664
Ga0376031_0013024_425_1351
TH5895 (JAATVS000000000)
TH526 (JAATVR000000000)
TH5896 (JAATVP000000000)
EAA2017_July_plot3_rep3_bin_15
Ga0376038_0004703_5959_6876
Ga0376034_0057198_2681_3598
Permafrost Thaumarchaeota bin (IMG3300012019_6)
Thaumarchaeota archaeon bin19 (IMG2698536741)
Permafrost Thaumarchaeota bin (IMG3300013763_3)
Ga0376037_0332976_227_1033
EAA AOA bin (IMG3300043331_1529)
Ga0376041_0047532_1747_2658
Ga0376033_0252253_145_1056
Ga0376039_0312384_71_979
EAA2017_July_plot3_rep3_bin_8
Ga0376037_0244400_2_799
Ga0376038_0493384_123_935
Ga0376030_0736705_214_909
Ga0376037_0022402_2638_3537
Ga0376040_0035736_733_1632
Ga0376031_0019660_2938_3837
Ga0376033_0197184_71_970
Ga0376030_0730190_1_711
TH4187 (JAATVO000000000)
Rhizosphere Thaumarchaeota bin (IMG3300027471_3)
Rhizosphere Thaumarchaeota bin (IMG3300027526_4)
Rhizosphere Thaumarchaeota bin (IMG3300027378_2)
Ga0376031_0467816_23_934
Groundwater Thaumarchaeota bin (IMG3300027947_1)
Groundwater Thaumarchaeota bin (IMG3300027187_1)
Groundwater Thaumarchaeota bin (IMG3300027384_1)
Groundwater Thaumarchaeota bin (IMG3300027273_1)
Ga0376032_0301134_230_1132
Ga0376031_0118132_1173_1877
Ga0376034_0360982_42_797
Rhizosphere Thaumarchaeota bin (IMG3300025315_14)
Ga0376033_0003558_3753_4652
Ga0376029_0338128_131_1030
Ca. Nitrososphaera gargensis Ga9.2 (WP_015018465.1)
Ga0376042_0171542_535_1155
Ga0376030_0269303_770_1576
Contaminated soil Thaumarchaeota bin (IMG3300033417_25)
EAA AOA bin (IMG3300043331_689)
Ga0376037_0146267_3_914
Ga0376030_0636408_109_987
Ca. Nitrososphaera evergladensis SR1 (WP_148701958.1)
Nitrososphaera viennensis EN76 (WP_084790910.1)
Ga0376030_0450990_316_1194
Ga0376029_0341065_149_1045
Ga0376042_0054123_443_1426
Ga0376033_0061469_3_854
Soil Thaumarchaeota bin (IMG3300010041_15)
EAA AOA bin (IMG3300043331_1378)
Rhizosphere Thaumarchaeota bin (IMG3300009147_53)
Family-level clades
f_Nitrosopelagicaceae
f_Nitrosopumilaceae
f_Nitrosotenuaceae
f_Nitrosotaleaceae
f_NS-zeta
f_NS-epsilon
f_NS-beta
f_NS-gamma
f_NS-beta-associated
f_NS-delta
f_Nitrososphaeraceae
Proportion (%)
0
100

Fig. S21. Maximum likelihood phylogeny of AOA PstA protein sequences recovered from metagenomes and metatranscriptomes in this study (left) and heat map of relative abundance based on read mapping (right). Representative sequences assembled in this study are indicated with their IMG accession number ("Ga*"). Reference sequences of cultured AOA genomes and MAGs retrieved from NCBI RefSeq and IMG databases were used to classify each phylotype to a family-level AOA lineage based on genome-wide taxonomic ranking system (Sheridan et al 2020). Ultrafast bootstrap values >70% and >90% (1,000 replicates) are indicated by black and red solid circles, respectively. The scale bar represents 0.5 substitution per amino acid position. Heat map shows the proportion of mapped sequencing reads of each phylotype relative to total AOA *pstA* reads in each metagenome or metatranscriptome. Shown are all three biological replicates, except for plot 3, for which only two metatranscriptomes were recovered in July 2017.

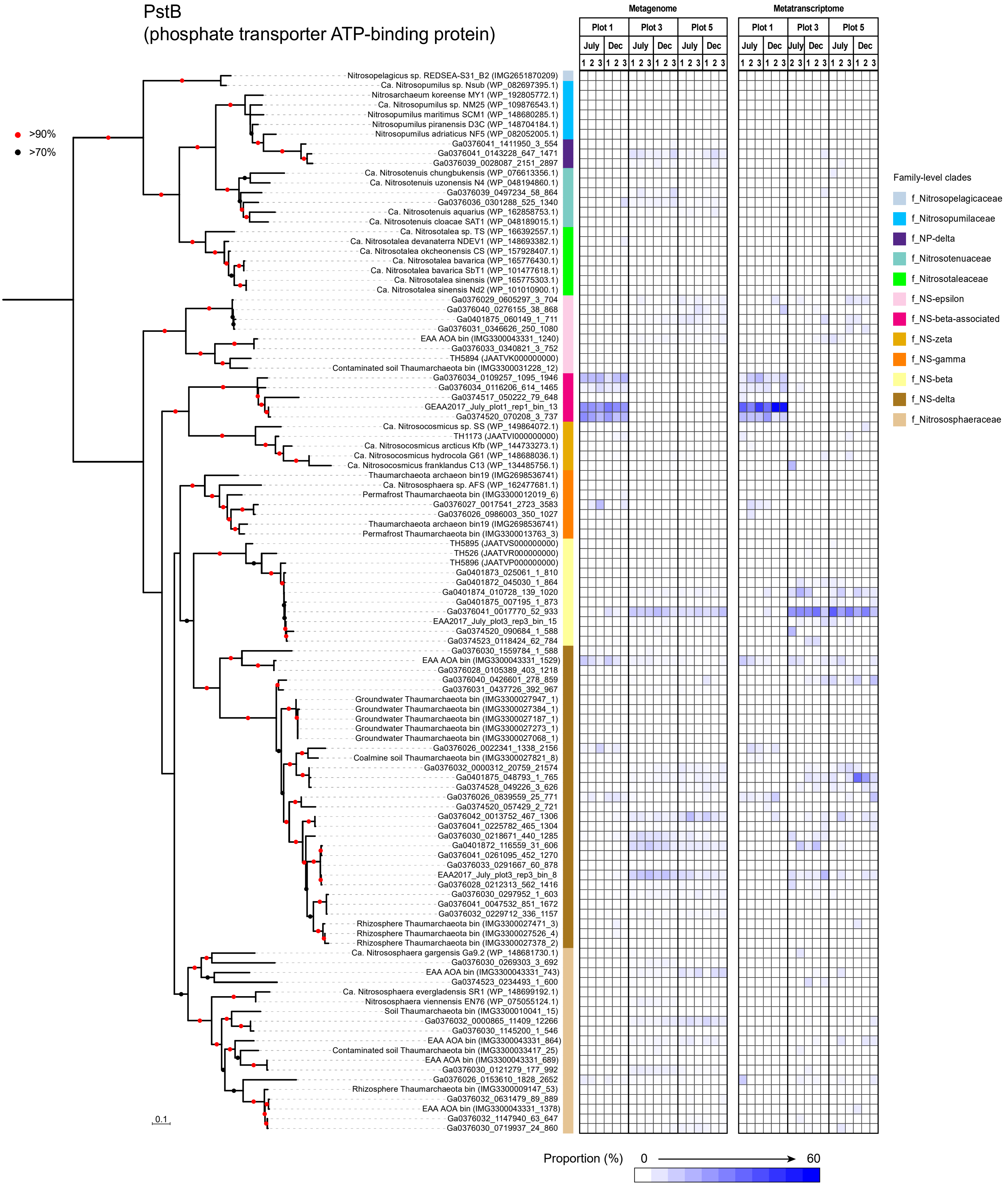

PstB
(phosphate transporter ATP-binding protein)
>90%
>70%
0.1
Metagenome
Metatranscriptome
Plot 1
Plot 3
Plot 5
July
Dec
1 2 3
Nitrosopelagicus sp. REDSEA-S31_B2 (IMG2651870209)
Ca. Nitrosopumilus sp. Nsub (WP_082697395.1)
Nitrosarchaeum koreense MY1 (WP_192805772.1)
Ca. Nitrosopumilus sp. NM25 (WP_109876543.1)
Nitrosopumilus maritimus SCM1 (WP_148680285.1)
Nitrosopumilus piranensis D3C (WP_148704184.1)
Nitrosopumilus adriaticus NF5 (WP_082052005.1)
Ga0376041_1411950_3_554
Ga0376041_0143228_647_1471
Ga0376039_0028087_2151_2897
Ca. Nitrosotenuis chungbukensis (WP_076613356.1)
Ca. Nitrosotenuis uzonensis N4 (WP_048194860.1)
Ga0376039_0497234_58_864
Ga0376036_0301288_525_1340
Ca. Nitrosotenuis aquarius (WP_162858753.1)
Ca. Nitrosotenuis cloacae SAT1 (WP_048189015.1)
Ca. Nitrosotalea sp. TS (WP_166392557.1)
Ca. Nitrosotalea devanaterra NDEV1 (WP_148693382.1)
Ca. Nitrosotalea okcheonensis CS (WP_157928407.1)
Ca. Nitrosotalea bavarica (WP_165776430.1)
Ca. Nitrosotalea bavarica SbT1 (WP_101477618.1)
Ca. Nitrosotalea sinensis (WP_165775303.1)
Ca. Nitrosotalea sinensis Nd2 (WP_101010900.1)
Ga0376029_0605297_3_704
Ga0376040_0276155_38_868
Ga0401875_060149_1_711
Ga0376031_0346626_250_1080
EAA AOA bin (IMG3300043331_1240)
Ga0376033_0340821_3_752
TH5894 (JAATVK000000000)
Contaminated soil Thaumarchaeota bin (IMG3300031228_12)
Ga0376034_0109257_1095_1946
Ga0376034_0116206_614_1465
Ga0374517_050222_79_648
GEAA2017_July_plot1_rep1_bin_13
Ga0374520_070208_3_737
Ca. Nitrosocosmicus sp. SS (WP_149864072.1)
TH1173 (JAATVI000000000)
Ca. Nitrosocosmicus arcticus Kfb (WP_144733273.1)
Ca. Nitrosocosmicus hydrocola G61 (WP_148688036.1)
Ca. Nitrosocosmicus franklandus C13 (WP_134485756.1)
Thaumarchaeota archaeon bin19 (IMG2698536741)
Ca. Nitrososphaera sp. AFS (WP_162477681.1)
Permafrost Thaumarchaeota bin (IMG3300012019_6)
Ga0376027_0017541_2723_3583
Ga0376026_0986003_350_1027
Thaumarchaeota archaeon bin19 (IMG2698536741)
Permafrost Thaumarchaeota bin (IMG3300013763_3)
TH5895 (JAATVS000000000)
TH526 (JAATVR000000000)
TH5896 (JAATVP000000000)
Ga0401873_025061_1_810
Ga0401872_045030_1_864
Ga0401874_010728_139_1020
Ga0401875_007195_1_873
Ga0376041_0017770_52_933
EAA2017_July_plot3_rep3_bin_15
Ga0374520_090684_1_588
Ga0374523_0118424_62_784
Ga0376030_1559784_1_588
EAA AOA bin (IMG3300043331_1529)
Ga0376028_0105389_403_1218
Ga0376040_0426601_278_859
Ga0376031_0437726_392_967
Groundwater Thaumarchaeota bin (IMG3300027947_1)
Groundwater Thaumarchaeota bin (IMG3300027384_1)
Groundwater Thaumarchaeota bin (IMG3300027187_1)
Groundwater Thaumarchaeota bin (IMG3300027273_1)
Groundwater Thaumarchaeota bin (IMG3300027068_1)
Ga0376026_0022341_1338_2156
Coalmine soil Thaumarchaeota bin (IMG3300027821_8)
Ga0376032_0000312_20759_21574
Ga0401875_048793_1_765
Ga0374528_049226_3_626
Ga0376026_0839559_25_771
Ga0374520_057429_2_721
Ga0376042_0013752_467_1306
Ga0376041_0225782_465_1304
Ga0376030_0218671_440_1285
Ga0401872_116559_31_606
Ga0376041_0261095_452_1270
Ga0376033_0291667_60_878
EAA2017_July_plot3_rep3_bin_8
Ga0376028_0212313_562_1416
Ga0376030_0297952_1_603
Ga0376041_0047532_851_1672
Ga0376032_0229712_336_1157
Rhizosphere Thaumarchaeota bin (IMG3300027471_3)
Rhizosphere Thaumarchaeota bin (IMG3300027526_4)
Rhizosphere Thaumarchaeota bin (IMG3300027378_2)
Ca. Nitrososphaera gargensis Ga9.2 (WP_148681730.1)
Ga0376030_0269303_3_692
EAA AOA bin (IMG3300043331_743)
Ga0374523_0234493_1_600
Ca. Nitrososphaera evergladensis SR1 (WP_148699192.1)
Nitrososphaera viennensis EN76 (WP_075055124.1)
Soil Thaumarchaeota bin (IMG3300010041_15)
Ga0376032_0000865_11409_12266
Ga0376030_1145200_1_546
EAA AOA bin (IMG3300043331_864)
Contaminated soil Thaumarchaeota bin (IMG3300033417_25)
EAA AOA bin (IMG3300043331_689)
Ga0376030_0121279_177_992
Ga0376026_0153610_1828_2652
Rhizosphere Thaumarchaeota bin (IMG3300009147_53)
Ga0376032_0631479_89_889
EAA AOA bin (IMG3300043331_1378)
Ga0376032_1147940_63_647
Ga0376030_0719937_24_860
Family-level clades
f_Nitrosopelagicaceae
f_Nitrosopumilaceae
f_NP-delta
f_Nitrosotenuaceae
f_Nitrosotaleaceae
f_NS-epsilon
f_NS-beta-associated
f_NS-zeta
f_NS-gamma
f_NS-beta
f_NS-delta
f_Nitrososphaeraceae
Proportion (%)
0
60

Fig. S22. Maximum likelihood phylogeny of AOA PstB protein sequences recovered from metagenomes and metatranscriptomes in this study (left) and heat map of relative abundance based on read mapping (right). Representative sequences assembled in this study are indicated with their IMG accession number ("Ga*"). Reference sequences of cultured AOA genomes and MAGs retrieved from NCBI RefSeq and IMG databases were used to classify each phylotype to a family-level AOA lineage based on genome-wide taxonomic ranking system (Sheridan et al 2020). Ultrafast bootstrap values >70% and >90% (1,000 replicates) are indicated by black and red solid circles, respectively. The scale bar represents 0.1 substitution per amino acid position. Heat map shows the proportion of mapped sequencing reads of each phylotype relative to total AOA *pstB* reads in each metagenome or metatranscriptome. Shown are all three biological replicates, except for plot 3, for which only two metatranscriptomes were recovered in July 2017.

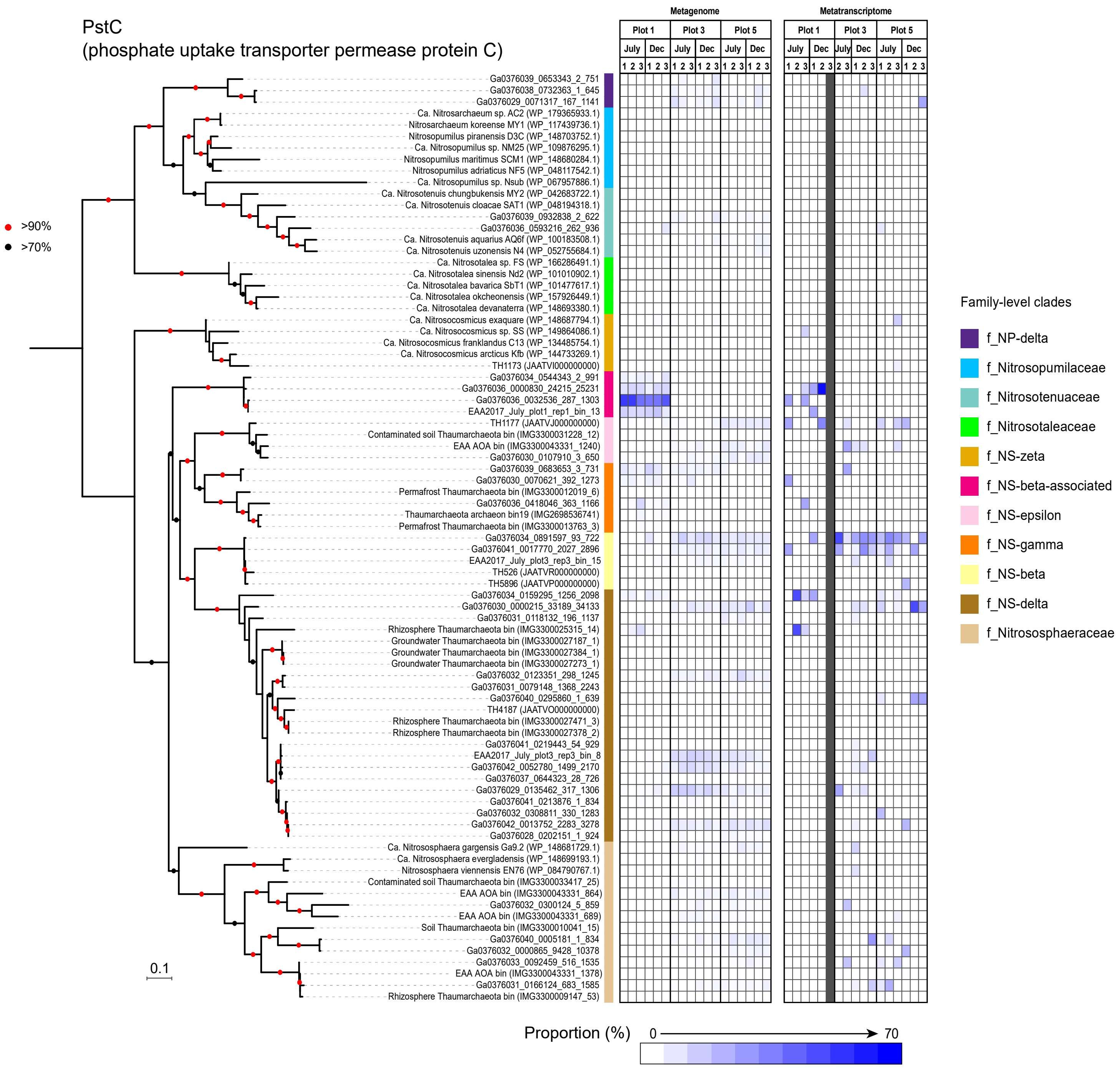
PstC
(phosphate uptake transporter permease protein C)
>90%
>70%
0.1
Ga0376039_0653343_2_751
Ga0376038_0732363_1_645
Ga0376029_0071317_167_1141
Ca. Nitrosarchaeum sp. AC2 (WP_179365933.1)
Nitrosarchaeum koreense MY1 (WP_117439736.1)
Nitrosopumilus piranensis D3C (WP_148703752.1)
Ca. Nitrosopumilus sp. NM25 (WP_109876295.1)
Nitrosopumilus maritimus SCM1 (WP_148680284.1)
Nitrosopumilus adriaticus NF5 (WP_048117542.1)
Ca. Nitrosopumilus sp. Nsub (WP_067957886.1)
Ca. Nitrosotenuis chungbukensis MY2 (WP_042683722.1)
Ca. Nitrosotenuis cloacae SAT1 (WP_048194318.1)
Ga0376039_0932838_2_622
Ga0376036_0593216_262_936
Ca. Nitrosotenuis aquarius AQ6f (WP_100183508.1)
Ca. Nitrosotenuis uzonensis N4 (WP_052755684.1)
Ca. Nitrosotalea sp. FS (WP_166286491.1)
Ca. Nitrosotalea sinensis Nd2 (WP_101010902.1)
Ca. Nitrosotalea bavarica SbT1 (WP_101477617.1)
Ca. Nitrosotalea okcheonensis (WP_157926449.1)
Ca. Nitrosotalea devanaterra (WP_148693380.1)
Ca. Nitrosocosmicus exaquare (WP_148687794.1)
Ca. Nitrosocosmicus sp. SS (WP_149864086.1)
Ca. Nitrosocosmicus franklandus C13 (WP_134485754.1)
Ca. Nitrosocosmicus arcticus Kfb (WP_144733269.1)
TH1173 (JAATVI000000000)
Ga0376034_0544343_2_991
Ga0376036_0000830_24215_25231
Ga0376036_0032536_287_1303
EAA2017_July_plot1_rep1_bin_13
TH1177 (JAATVJ000000000)
Contaminated soil Thaumarchaeota bin (IMG3300031228_12)
EAA AOA bin (IMG3300043331_1240)
Ga0376030_0107910_3_650
Ga0376039_0683653_3_731
Ga0376030_0070621_392_1273
Permafrost Thaumarchaeota bin (IMG3300012019_6)
Ga0376036_0418046_363_1166
Thaumarchaeota archaeon bin19 (IMG2698536741)
Permafrost Thaumarchaeota bin (IMG3300013763_3)
Ga0376034_0891597_93_722
Ga0376041_0017770_2027_2896
EAA2017_July_plot3_rep3_bin_15
TH526 (JAATVR000000000)
TH5896 (JAATVP000000000)
Ga0376034_0159295_1256_2098
Ga0376030_0000215_33189_34133
Ga0376031_0118132_196_1137
Rhizosphere Thaumarchaeota bin (IMG3300025315_14)
Groundwater Thaumarchaeota bin (IMG3300027187_1)
Groundwater Thaumarchaeota bin (IMG3300027384_1)
Groundwater Thaumarchaeota bin (IMG3300027273_1)
Ga0376032_0123351_298_1245
Ga0376031_0079148_1368_2243
Ga0376040_0295860_1_639
TH4187 (JAATVO000000000)
Rhizosphere Thaumarchaeota bin (IMG3300027471_3)
Rhizosphere Thaumarchaeota bin (IMG3300027378_2)
Ga0376041_0219443_54_929
EAA2017_July_plot3_rep3_bin_8
Ga0376042_0052780_1499_2170
Ga0376037_0644323_28_726
Ga0376029_0135462_317_1306
Ga0376041_0213876_1_834
Ga0376032_0308811_330_1283
Ga0376042_0013752_2283_3278
Ga0376028_0202151_1_924
Ca. Nitrososphaera gargensis Ga9.2 (WP_148681729.1)
Ca. Nitrososphaera evergladensis (WP_148699193.1)
Nitrososphaera viennensis EN76 (WP_084790767.1)
Contaminated soil Thaumarchaeota bin (IMG3300033417_25)
EAA AOA bin (IMG3300043331_864)
Ga0376032_0300124_5_859
EAA AOA bin (IMG3300043331_689)
Soil Thaumarchaeota bin (IMG3300010041_15)
Ga0376040_0005181_1_834
Ga0376032_0000865_9428_10378
Ga0376033_0092459_516_1535
EAA AOA bin (IMG3300043331_1378)
Ga0376031_0166124_683_1585
Rhizosphere Thaumarchaeota bin (IMG3300009147_53)
Metagenome
Metatranscriptome
Plot 1
Plot 3
Plot 5
July
Dec
1 2 3
Family-level clades
f_NP-delta
f_Nitrosopumilaceae
f_Nitrosotenuaceae
f_Nitrosotaleaceae
f_NS-zeta
f_NS-beta-associated
f_NS-epsilon
f_NS-gamma
f_NS-beta
f_NS-delta
f_Nitrososphaeraceae
Proportion (%)
0
70

Fig. S23. Maximum likelihood phylogeny of AOA PstC protein sequences recovered from metagenomes and metatranscriptomes in this study (left) and heat map of relative abundance based on read mapping (right). Representative sequences assembled in this study are indicated with their IMG accession number ("Ga*"). Reference sequences of cultured AOA genomes and MAGs retrieved from NCBI RefSeq and IMG databases were used to classify each phylotype to a family-level AOA lineage based on genome-wide taxonomic ranking system (Sheridan et al 2020). Ultrafast bootstrap values >70% and >90% (1,000 replicates) are indicated by black and red solid circles, respectively. The scale bar represents 0.1 substitution per amino acid position. Heat map shows the proportion of mapped sequencing reads of each phylotype relative to total AOA *pstC* reads in each metagenome or metatranscriptome. Shown are all three biological replicates, except for plot 3, for which only two metatranscriptomes were recovered in July 2017.

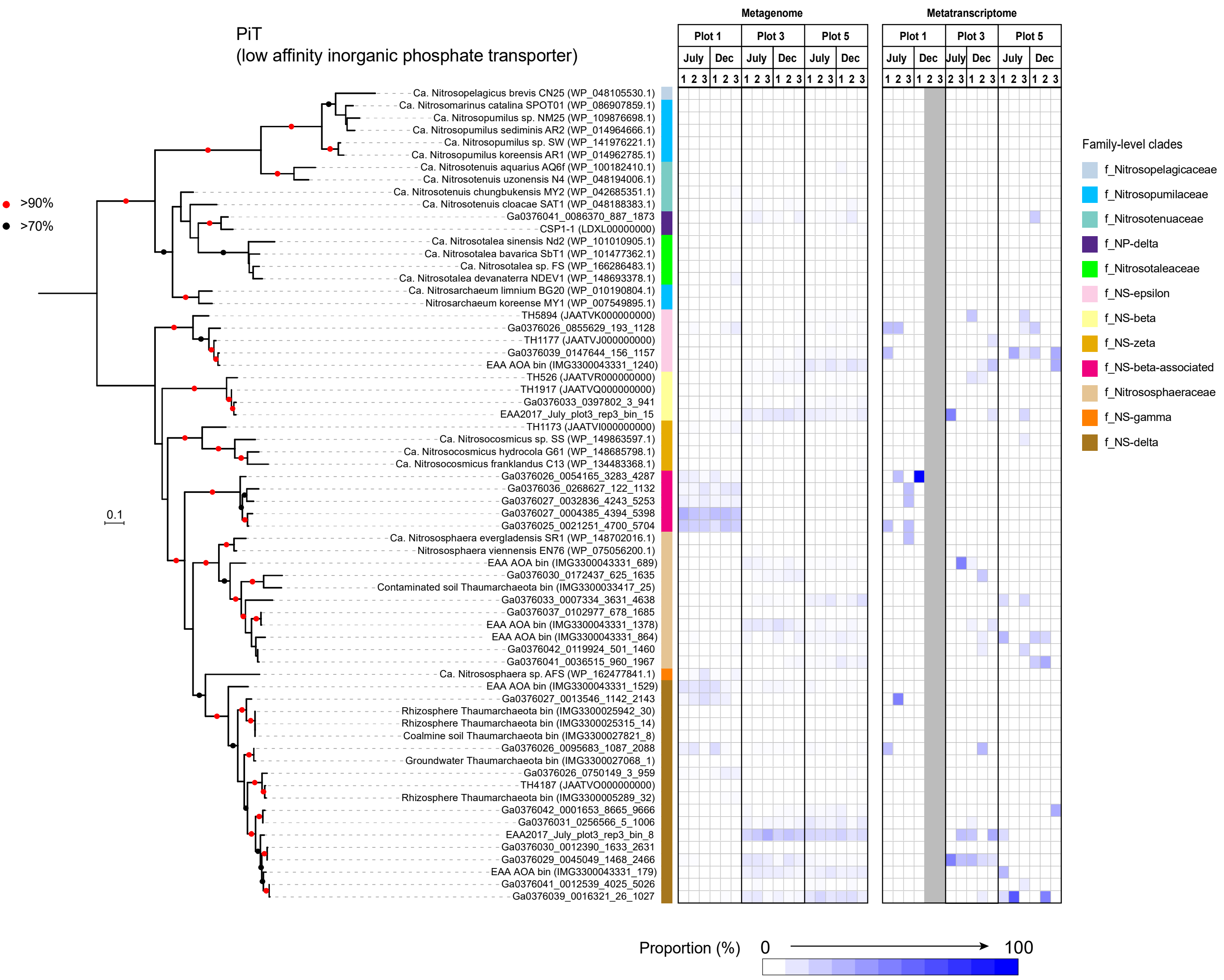

PiT
(low affinity inorganic phosphate transporter)
>90%
>70%
0.1
Metagenome
Metatranscriptome
Plot 1
Plot 3
Plot 5
July
Dec
1 2 3
Ca. Nitrosopelagicus brevis CN25 (WP_048105530.1)
Ca. Nitrosomarinus catalina SPOT01 (WP_086907859.1)
Ca. Nitrosopumilus sp. NM25 (WP_109876698.1)
Ca. Nitrosopumilus sediminis AR2 (WP_014964666.1)
Ca. Nitrosopumilus sp. SW (WP_141976221.1)
Ca. Nitrosopumilus koreensis AR1 (WP_014962785.1)
Ca. Nitrosotenuis aquarius AQ6f (WP_100182410.1)
Ca. Nitrosotenuis uzonensis N4 (WP_048194006.1)
Ca. Nitrosotenuis chungbukensis MY2 (WP_042685351.1)
Ca. Nitrosotenuis cloacae SAT1 (WP_048188383.1)
Ga0376041_0086370_887_1873
CSP1-1 (LDXL00000000)
Ca. Nitrosotalea sinensis Nd2 (WP_101010905.1)
Ca. Nitrosotalea bavarica SbT1 (WP_101477362.1)
Ca. Nitrosotalea sp. FS (WP_166286483.1)
Ca. Nitrosotalea devanaterra NDEV1 (WP_148693378.1)
Ca. Nitrosarchaeum limnium BG20 (WP_010190804.1)
Nitrosarchaeum koreense MY1 (WP_007549895.1)
TH5894 (JAATVK000000000)
Ga0376026_0855629_193_1128
TH1177 (JAATVJ000000000)
Ga0376039_0147644_156_1157
EAA AOA bin (IMG3300043331_1240)
TH526 (JAATVR000000000)
TH1917 (JAATVQ000000000)
Ga0376033_0397802_3_941
EAA2017_July_plot3_rep3_bin_15
TH1173 (JAATVI000000000)
Ca. Nitrosocosmicus sp. SS (WP_149863597.1)
Ca. Nitrosocosmicus hydrocola G61 (WP_148685798.1)
Ca. Nitrosocosmicus franklandus C13 (WP_134483368.1)
Ga0376026_0054165_3283_4287
Ga0376036_0268627_122_1132
Ga0376027_0032836_4243_5253
Ga0376027_0004385_4394_5398
Ga0376025_0021251_4700_5704
Ca. Nitrososphaera evergladensis SR1 (WP_148702016.1)
Nitrososphaera viennensis EN76 (WP_075056200.1)
EAA AOA bin (IMG3300043331_689)
Ga0376030_0172437_625_1635
Contaminated soil Thaumarchaeota bin (IMG3300033417_25)
Ga0376033_0007334_3631_4638
Ga0376037_0102977_678_1685
EAA AOA bin (IMG3300043331_1378)
EAA AOA bin (IMG3300043331_864)
Ga0376042_0119924_501_1460
Ga0376041_0036515_960_1967
Ca. Nitrososphaera sp. AFS (WP_162477841.1)
EAA AOA bin (IMG3300043331_1529)
Ga0376027_0013546_1142_2143
Rhizosphere Thaumarchaeota bin (IMG3300025942_30)
Rhizosphere Thaumarchaeota bin (IMG3300025315_14)
Coalmine soil Thaumarchaeota bin (IMG3300027821_8)
Ga0376026_0095683_1087_2088
Groundwater Thaumarchaeota bin (IMG3300027068_1)
Ga0376026_0750149_3_959
TH4187 (JAATVO000000000)
Rhizosphere Thaumarchaeota bin (IMG3300005289_32)
Ga0376042_0001653_8665_9666
Ga0376031_0256566_5_1006
EAA2017_July_plot3_rep3_bin_8
Ga0376030_0012390_1633_2631
Ga0376029_0045049_1468_2466
EAA AOA bin (IMG3300043331_179)
Ga0376041_0012539_4025_5026
Ga0376039_0016321_26_1027
Family-level clades
f_Nitrosopelagicaceae
f_Nitrosopumilaceae
f_Nitrosotenuaceae
f_NP-delta
f_Nitrosotaleaceae
f_NS-epsilon
f_NS-beta
f_NS-zeta
f_NS-beta-associated
f_Nitrososphaeraceae
f_NS-gamma
f_NS-delta
Proportion (%)
0
100

Fig. S24. Maximum likelihood phylogeny of AOA PiT protein sequences recovered from metagenomes and metatranscriptomes in this study (left) and heat map of relative abundance based on read mapping (right). Representative sequences assembled in this study are indicated with their IMG accession number ("Ga*"). Reference sequences of cultured AOA genomes and MAGs retrieved from NCBI RefSeq and IMG databases were used to classify each phylotype to a family-level AOA lineage based on genome-wide taxonomic ranking system (Sheridan et al 2020). Ultrafast bootstrap values >70% and >90% (1,000 replicates) are indicated by black and red solid circles, respectively. The scale bar represents 0.1 substitution per amino acid position. Heat map shows the proportion of mapped sequencing reads of each phylotype relative to total AOA *pit* reads in each metagenome or metatranscriptome. Shown are all three biological replicates, except for plot 3, for which only two metatranscriptomes were recovered in July 2017.

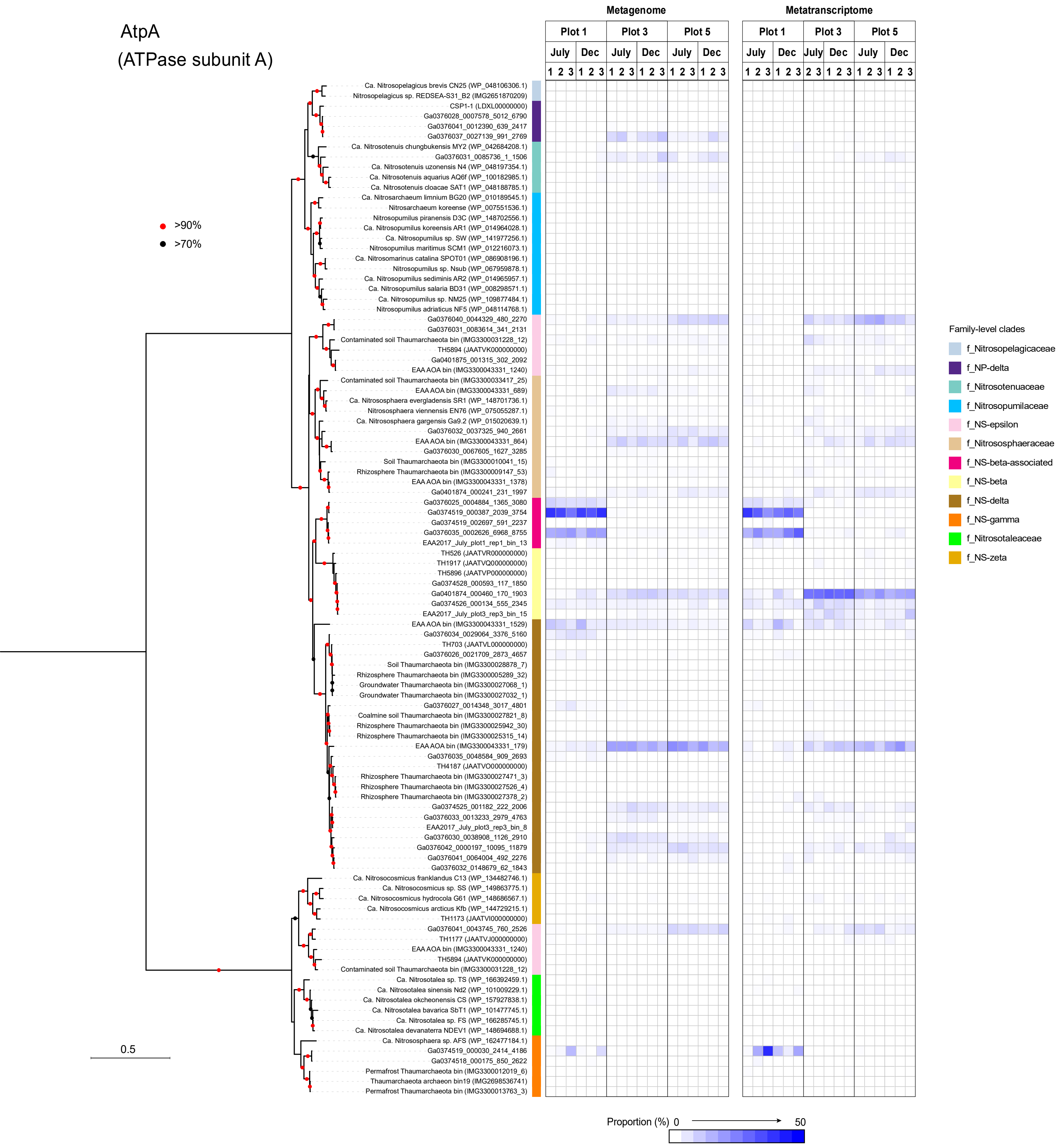
AtpA
(ATPase subunit A)
>90%
>70%
0.5
Metagenome
Metatranscriptome
Plot 1
Plot 3
Plot 5
July
Dec
Family-level clades
f_Nitrosopelagicaceae
f_NP-delta
f_Nitrosotenuaceae
f_Nitrosopumilaceae
f_NS-epsilon
f_Nitrososphaeraceae
f_NS-beta-associated
f_NS-beta
f_NS-delta
f_NS-gamma
f_Nitrosotaleaceae
f_NS-zeta
Proportion (%) 0
50

Fig. S25. Maximum likelihood phylogeny of AOA AtpA protein sequences recovered from metagenomes and metatranscriptomes in this study (left) and heat map of relative abundance based on read mapping (right). Representative sequences assembled in this study are indicated with their IMG accession number ("Ga*"). Reference sequences of cultured AOA genomes and MAGs retrieved from NCBI RefSeq and IMG databases were used to classify each phylotype to a family-level AOA lineage based on genome-wide taxonomic ranking system (Sheridan et al 2020). Ultrafast bootstrap values >70% and >90% (1,000 replicates) are indicated by black and red solid circles, respectively. The scale bar represents 0.5 substitution per amino acid position. Heat map shows the proportion of mapped sequencing reads of each phylotype relative to total AOA *atpA* reads in each metagenome or metatranscriptome. Shown are all three biological replicates, except for plot 3, for which only two metatranscriptomes were recovered in July 2017.

FlaB
(Archaeal flagellin)

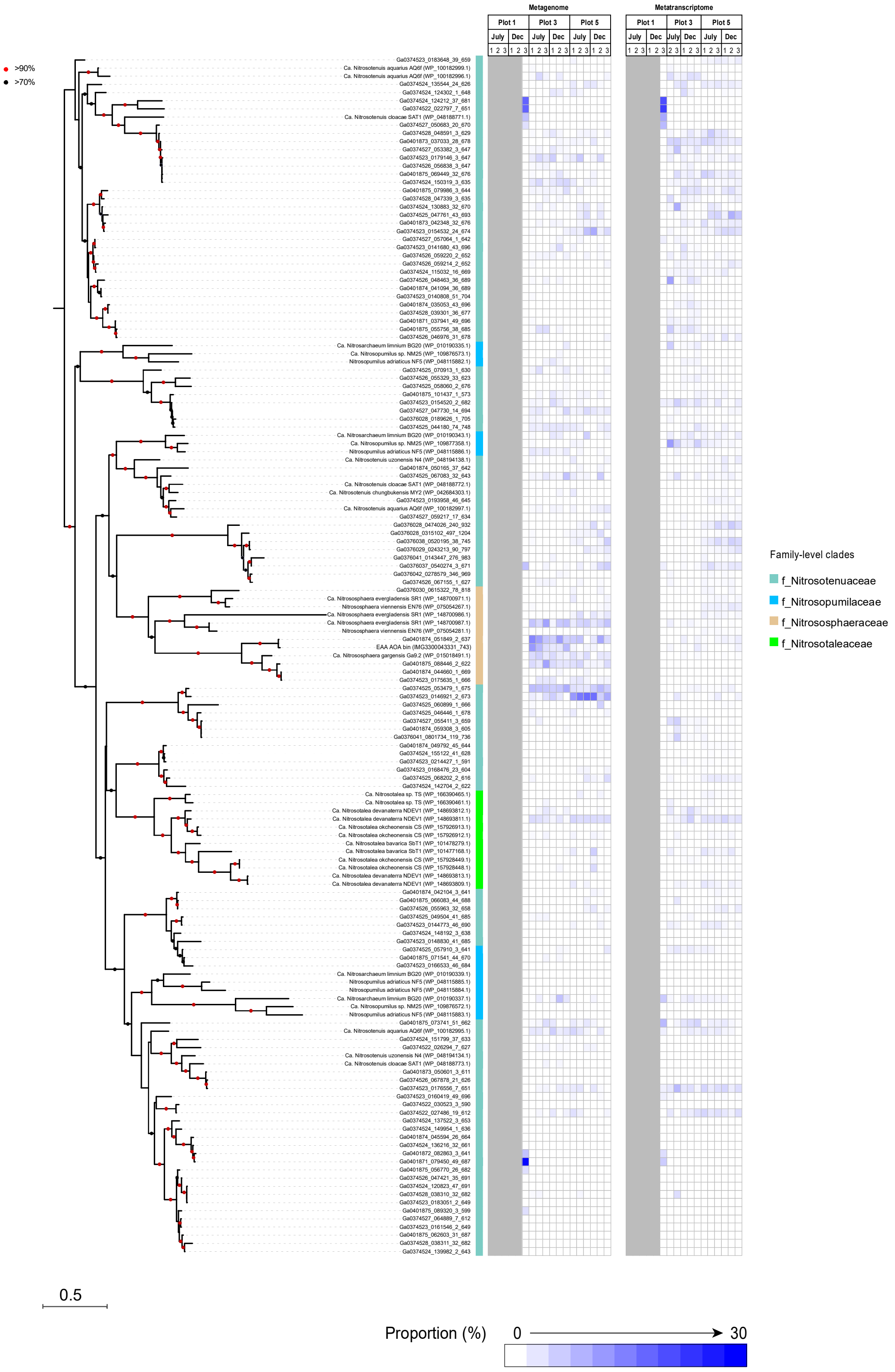

Fig. S26. Maximum likelihood phylogeny of AOA FlaB protein sequences recovered from metagenomes and metatranscriptomes in this study (left) and heat map of relative abundance based on read mapping (right). Representative sequences assembled in this study are indicated with their IMG accession number ("Ga*"). Reference sequences of cultured AOA genomes and MAGs retrieved from NCBI RefSeq and IMG databases were used to classify each phylotype to a family-level AOA lineage based on genome-wide taxonomic ranking system (Sheridan et al 2020). Ultrafast bootstrap values >70% and >90% (1,000 replicates) are indicated by black and red solid circles, respectively. The scale bar represents 0.5 substitution per amino acid position. Heat map shows the proportion of mapped sequencing reads of each phylotype relative to total AOA *flaB* reads in each metagenome or metatranscriptome. Shown are all three biological replicates, except for plot 3, for which only two metatranscriptomes were recovered in July 2017.

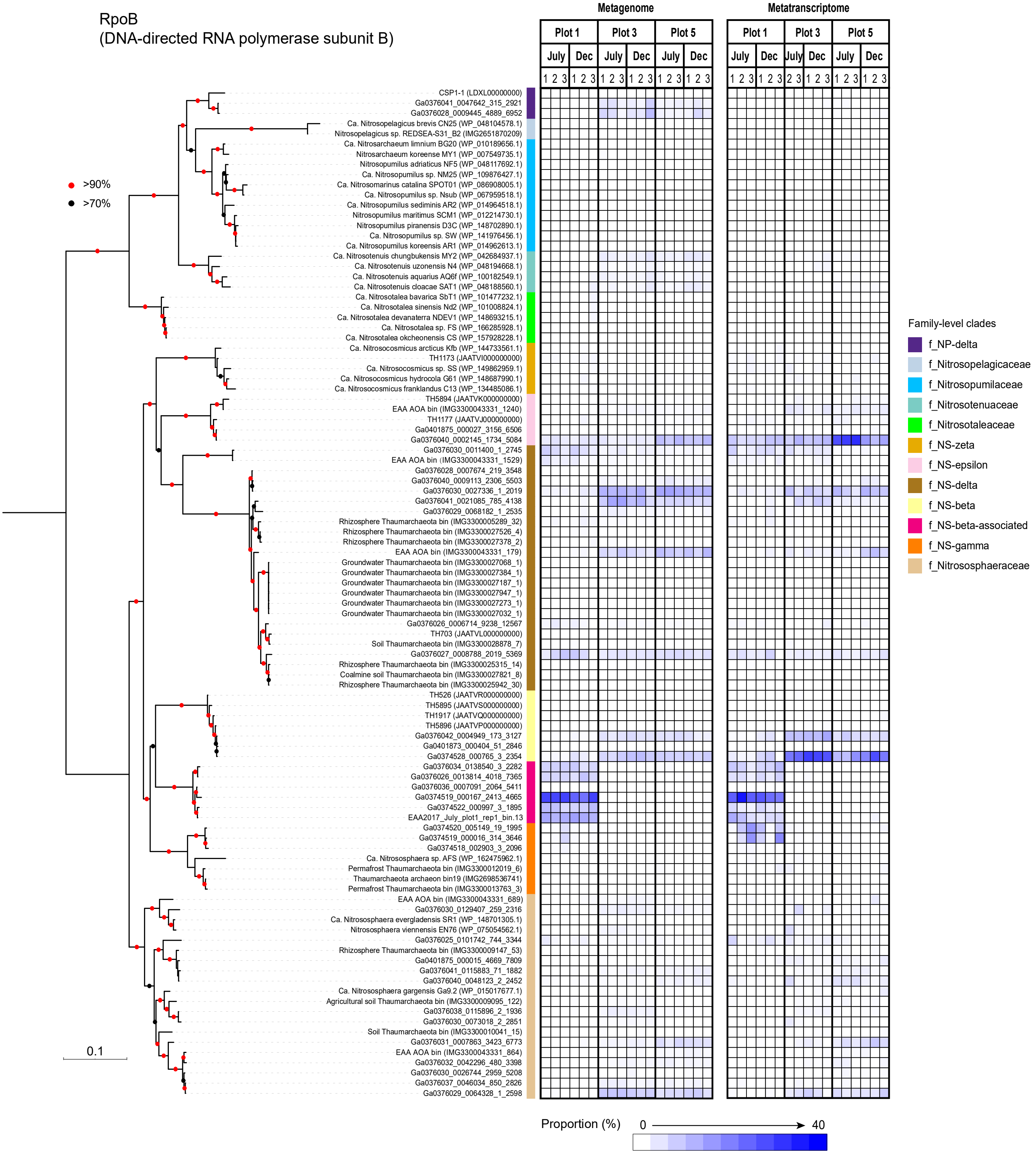

RpoB
(DNA-directed RNA polymerase subunit B)
>90%
>70%
0.1
CSP1-1 (LDXL00000000)
Ga0376041_0047642_315_2921
Ga0376028_0009445_4889_6952
Ca. Nitrosopelagicus brevis CN25 (WP_048104578.1)
Nitrosopelagicus sp. REDSEA-S31_B2 (IMG2651870209)
Ca. Nitrosarchaeum limnium BG20 (WP_010189656.1)
Nitrosarchaeum koreense MY1 (WP_007549735.1)
Nitrosopumilus adriaticus NF5 (WP_048117692.1)
Ca. Nitrosopumilus sp. NM25 (WP_109876427.1)
Ca. Nitrosomarinus catalina SPOT01 (WP_086908005.1)
Ca. Nitrosopumilus sp. Nsub (WP_067959518.1)
Ca. Nitrosopumilus sediminis AR2 (WP_014964518.1)
Nitrosopumilus maritimus SCM1 (WP_012214730.1)
Nitrosopumilus piranensis D3C (WP_148702890.1)
Ca. Nitrosopumilus sp. SW (WP_141976456.1)
Ca. Nitrosopumilus koreensis AR1 (WP_014962613.1)
Ca. Nitrosotenuis chungbukensis MY2 (WP_042684937.1)
Ca. Nitrosotenuis uzonensis N4 (WP_048194668.1)
Ca. Nitrosotenuis aquarius AQ6f (WP_100182549.1)
Ca. Nitrosotenuis cloacae SAT1 (WP_048188560.1)
Ca. Nitrosotalea bavarica SbT1 (WP_101477232.1)
Ca. Nitrosotalea sinensis Nd2 (WP_101008824.1)
Ca. Nitrosotalea devanaterra NDEV1 (WP_148693215.1)
Ca. Nitrosotalea sp. FS (WP_166285928.1)
Ca. Nitrosotalea okcheonensis CS (WP_157928228.1)
Ca. Nitrosocosmicus arcticus Kfb (WP_144733561.1)
TH1173 (JAATVI000000000)
Ca. Nitrosocosmicus sp. SS (WP_149862959.1)
Ca. Nitrosocosmicus hydrocola G61 (WP_148687990.1)
Ca. Nitrosocosmicus franklandus C13 (WP_134485086.1)
TH5894 (JAATVK000000000)
EAA AOA bin (IMG3300043331_1240)
TH1177 (JAATVJ000000000)
Ga0401875_000027_3156_6506
Ga0376040_0002145_1734_5084
Ga0376030_0011400_1_2745
EAA AOA bin (IMG3300043331_1529)
Ga0376028_0007674_219_3548
Ga0376040_0009113_2306_5503
Ga0376030_0027336_1_2019
Ga0376041_0021085_785_4138
Ga0376029_0068182_1_2535
Rhizosphere Thaumarchaeota bin (IMG3300005289_32)
Rhizosphere Thaumarchaeota bin (IMG3300027526_4)
Rhizosphere Thaumarchaeota bin (IMG3300027378_2)
EAA AOA bin (IMG3300043331_179)
Groundwater Thaumarchaeota bin (IMG3300027068_1)
Groundwater Thaumarchaeota bin (IMG3300027384_1)
Groundwater Thaumarchaeota bin (IMG3300027187_1)
Groundwater Thaumarchaeota bin (IMG3300027947_1)
Groundwater Thaumarchaeota bin (IMG3300027273_1)
Groundwater Thaumarchaeota bin (IMG3300027032_1)
Ga0376026_0006714_9238_12567
TH703 (JAATVL000000000)
Soil Thaumarchaeota bin (IMG3300028878_7)
Ga0376027_0008788_2019_5369
Rhizosphere Thaumarchaeota bin (IMG3300025315_14)
Coalmine soil Thaumarchaeota bin (IMG3300027821_8)
Rhizosphere Thaumarchaeota bin (IMG3300025942_30)
TH526 (JAATVR000000000)
TH5895 (JAATVS000000000)
TH1917 (JAATVQ000000000)
TH5896 (JAATVP000000000)
Ga0376042_0004949_173_3127
Ga0401873_000404_51_2846
Ga0374528_000765_3_2354
Ga0376034_0138540_3_2282
Ga0376026_0013814_4018_7365
Ga0376036_0007091_2064_5411
Ga0374519_000167_2413_4665
Ga0374522_000997_3_1895
EAA2017_July_plot1_rep1_bin.13
Ga0374520_005149_19_1995
Ga0374519_000016_314_3646
Ga0374518_002903_3_2096
Ca. Nitrososphaera sp. AFS (WP_162475962.1)
Permafrost Thaumarchaeota bin (IMG3300012019_6)
Thaumarchaeota archaeon bin19 (IMG2698536741)
Permafrost Thaumarchaeota bin (IMG3300013763_3)
EAA AOA bin (IMG3300043331_689)
Ga0376030_0129407_259_2316
Ca. Nitrososphaera evergladensis SR1 (WP_148701305.1)
Nitrososphaera viennensis EN76 (WP_075054562.1)
Ga0376025_0101742_744_3344
Rhizosphere Thaumarchaeota bin (IMG3300009147_53)
Ga0401875_000015_4669_7809
Ga0376041_0115883_71_1882
Ga0376040_0048123_2_2452
Ca. Nitrososphaera gargensis Ga9.2 (WP_015017677.1)
Agricultural soil Thaumarchaeota bin (IMG3300009095_122)
Ga0376038_0115896_2_1936
Ga0376030_0073018_2_2851
Soil Thaumarchaeota bin (IMG3300010041_15)
Ga0376031_0007863_3423_6773
EAA AOA bin (IMG3300043331_864)
Ga0376032_0042296_480_3398
Ga0376030_0026744_2959_5208
Ga0376037_0046034_850_2826
Ga0376029_0064328_1_2598
Metagenome
Metatranscriptome
Plot 1
Plot 3
Plot 5
July
Dec
1 2 3
Family-level clades
f_NP-delta
f_Nitrosopelagicaceae
f_Nitrosopumilaceae
f_Nitrosotenuaceae
f_Nitrosotaleaceae
f_NS-zeta
f_NS-epsilon
f_NS-delta
f_NS-beta
f_NS-beta-associated
f_NS-gamma
f_Nitrososphaeraceae
Proportion (%)
0
40

Fig. S27. Maximum likelihood phylogeny of AOA RpoB protein sequences recovered from metagenomes and metatranscriptomes in this study (left) and heat map of relative abundance based on read mapping (right). Representative sequences assembled in this study are indicated with their IMG accession number ("Ga*"). Reference sequences of cultured AOA genomes and MAGs retrieved from NCBI RefSeq and IMG databases were used to classify each phylotype to a family-level AOA lineage based on genome-wide taxonomic ranking system (Sheridan et al 2020). Ultrafast bootstrap values >70% and >90% (1,000 replicates) are indicated by black and red solid circles, respectively. The scale bar represents 0.1 substitution per amino acid position. Heat map shows the proportion of mapped sequencing reads of each phylotype relative to total AOA *rpoB* reads in each metagenome or metatranscriptome. Shown are all three biological replicates, except for plot 3, for which only two metatranscriptomes were recovered in July 2017.

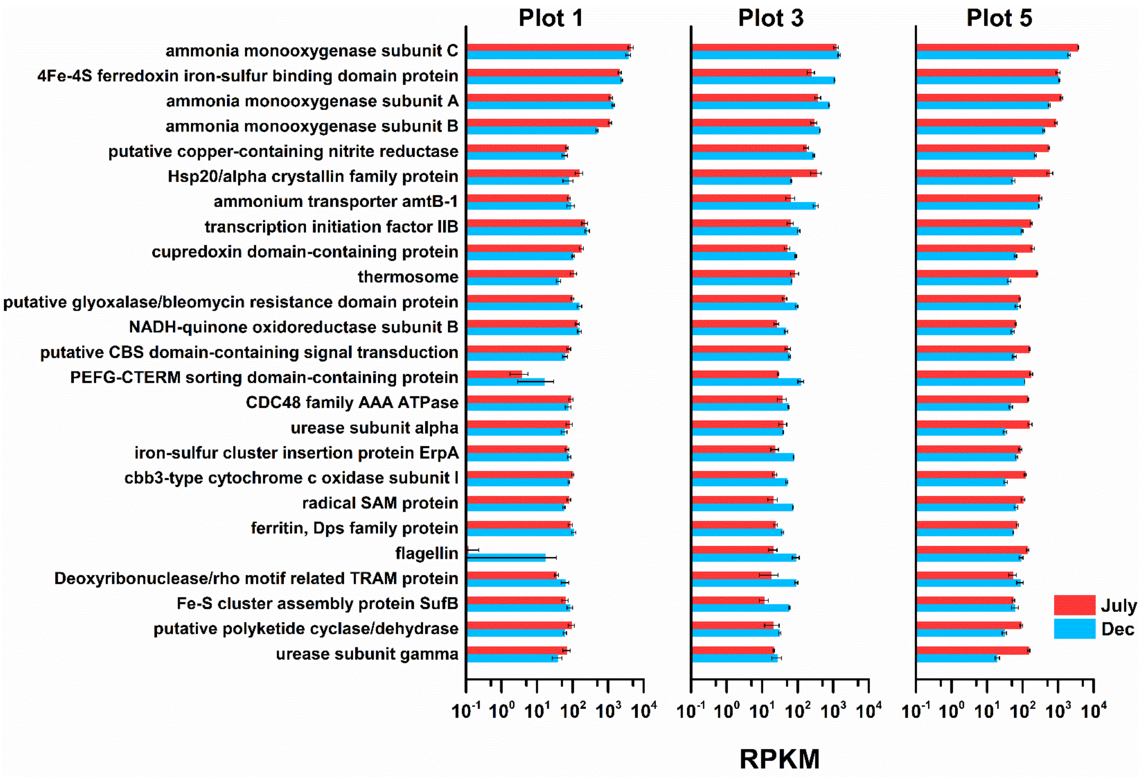

Fig. S28. **Top 25 most actively expressed genes of ammonia-oxidizing archaea (AOA) in different plots and seasons (July and December of 2017).** Columns show the relative abundances of gene transcripts (reads per kilobase per million mapped reads [RPKM]). Error bars represent standard errors of relative abundances from triplicate samples.

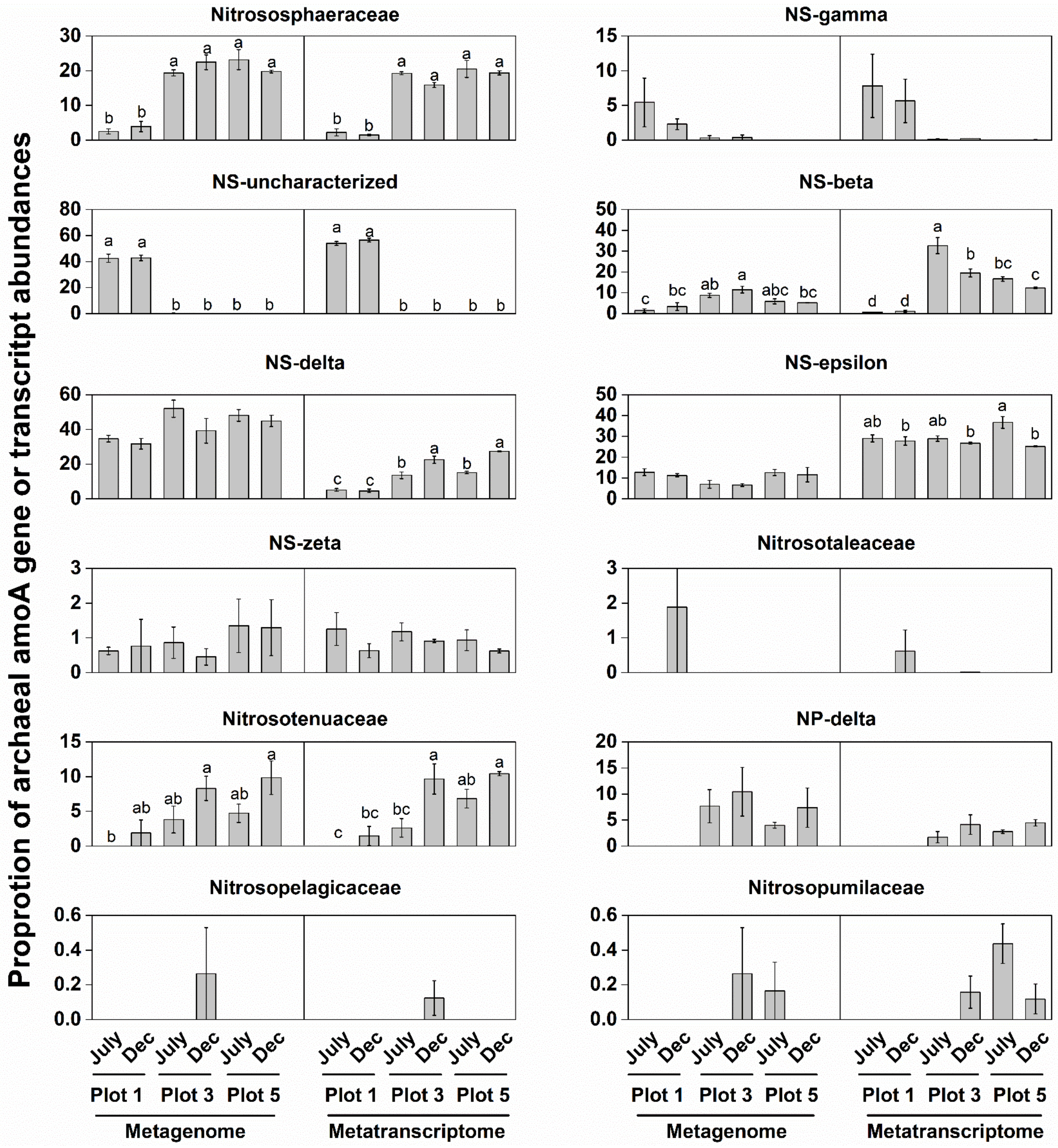

Fig. S29. **The proportion of archaeal *amoA* gene and transcript abundance in different AOA lineages in soils.** Error bars represent standard errors of relative abundances from triplicate samples and different letters above columns indicate significant difference in each plot.

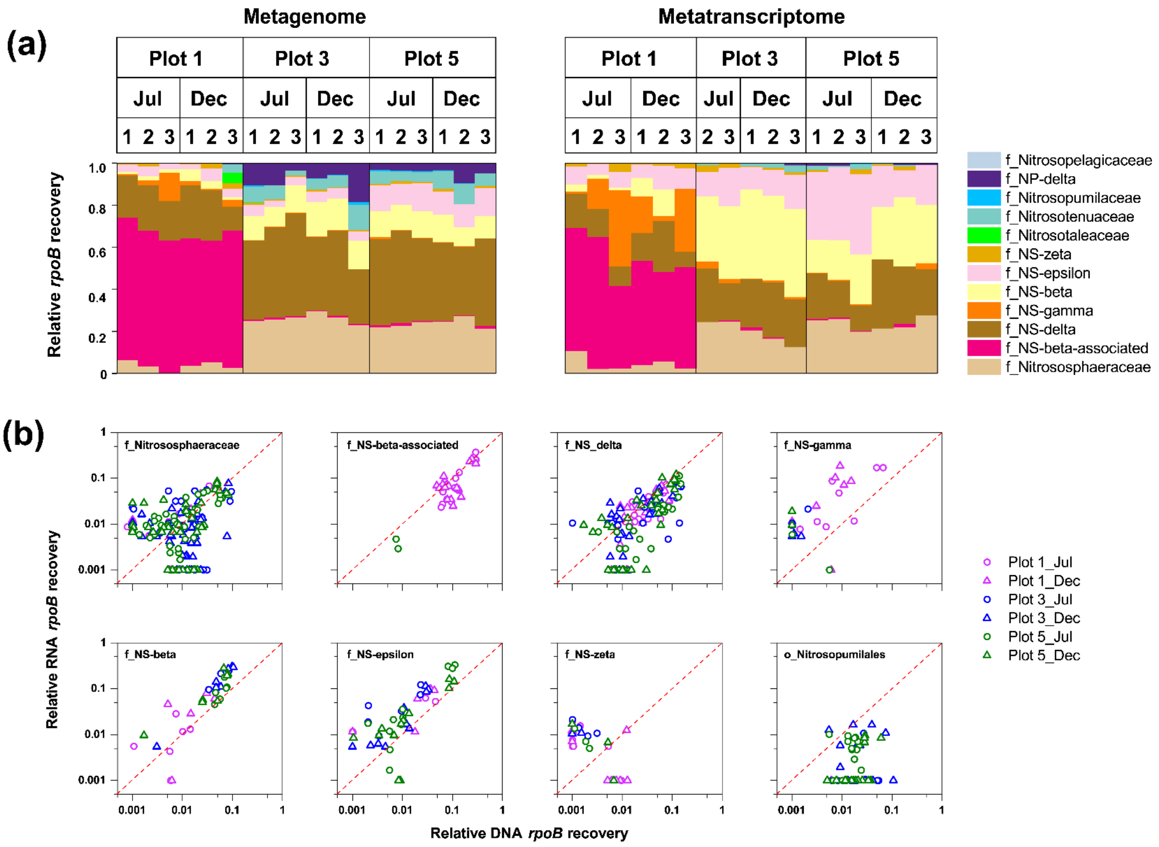

Fig. S30. Recovery frequencies of *rpoB* reads of AOA families (a) and phylotypes (b) relative to total AOA *rpoB* read numbers from metagenome and metatranscriptome data. (b) A symbol (circle or triangle) in each plot represents the relative *rpoB* DNA and RNA recovery frequencies of an AOA phylotype (100% protein sequence identity). The red dotted line across each plot indicates the same recovery frequency at both DNA and RNA levels, showing activity of a phylotype proportionate to corresponding relative abundance. Phylotypes landing on area above the red dotted line show disproportionately high activity and phylotypes below the red line have disproportionately low activity or potential inactivity.

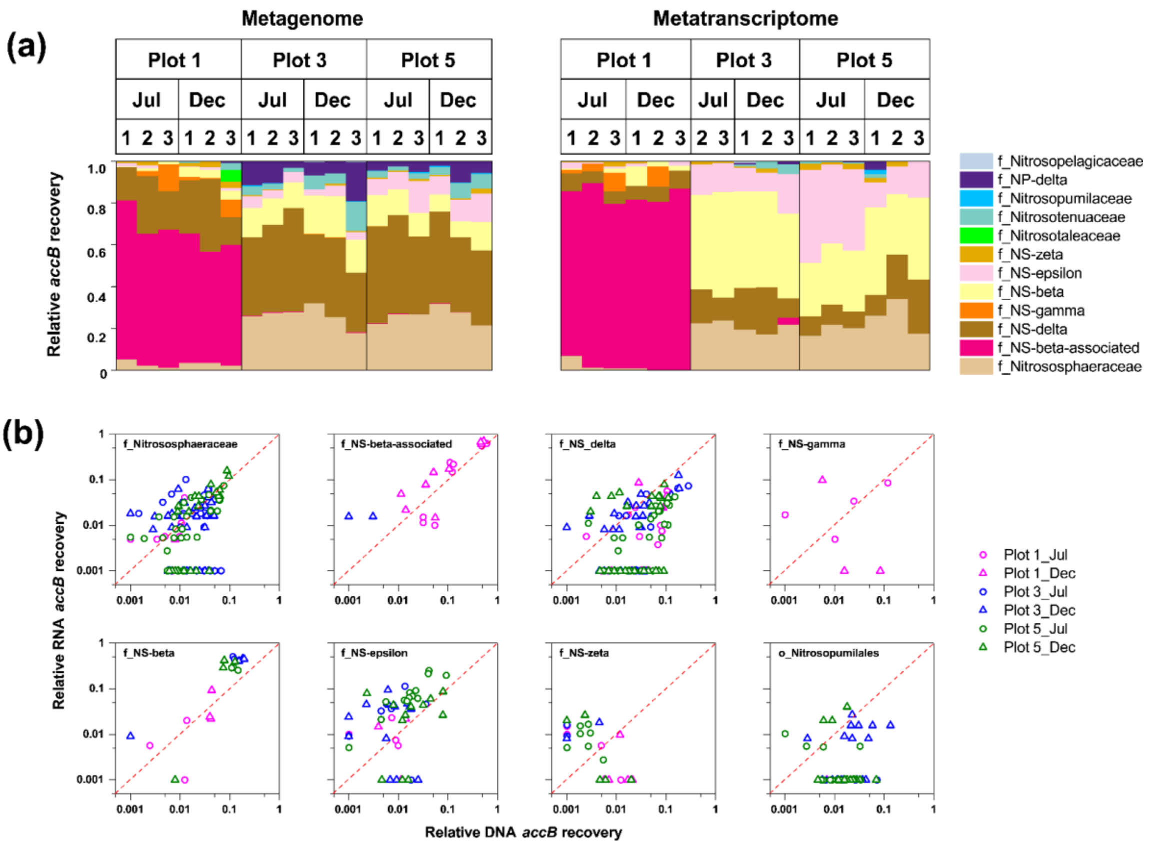

Fig. S31. **Recovery frequencies of *accB* reads of AOA families (a) and phylotypes (b) relative to total AOA *accB* read numbers from metagenome and metatranscriptome data.** (b) A symbol (circle or triangle) in each plot represents the relative *accB* DNA and RNA recovery frequencies of an AOA phylotype (100% protein sequence identity). The red dotted line across each plot indicates the same recovery frequency at both DNA and RNA levels, showing activity of a phylotype proportionate to corresponding relative abundance. phylotypes landing on area above the red dotted line show disproportionately high activity and phylotypes below the red line have disproportionately low activity or potential inactivity.

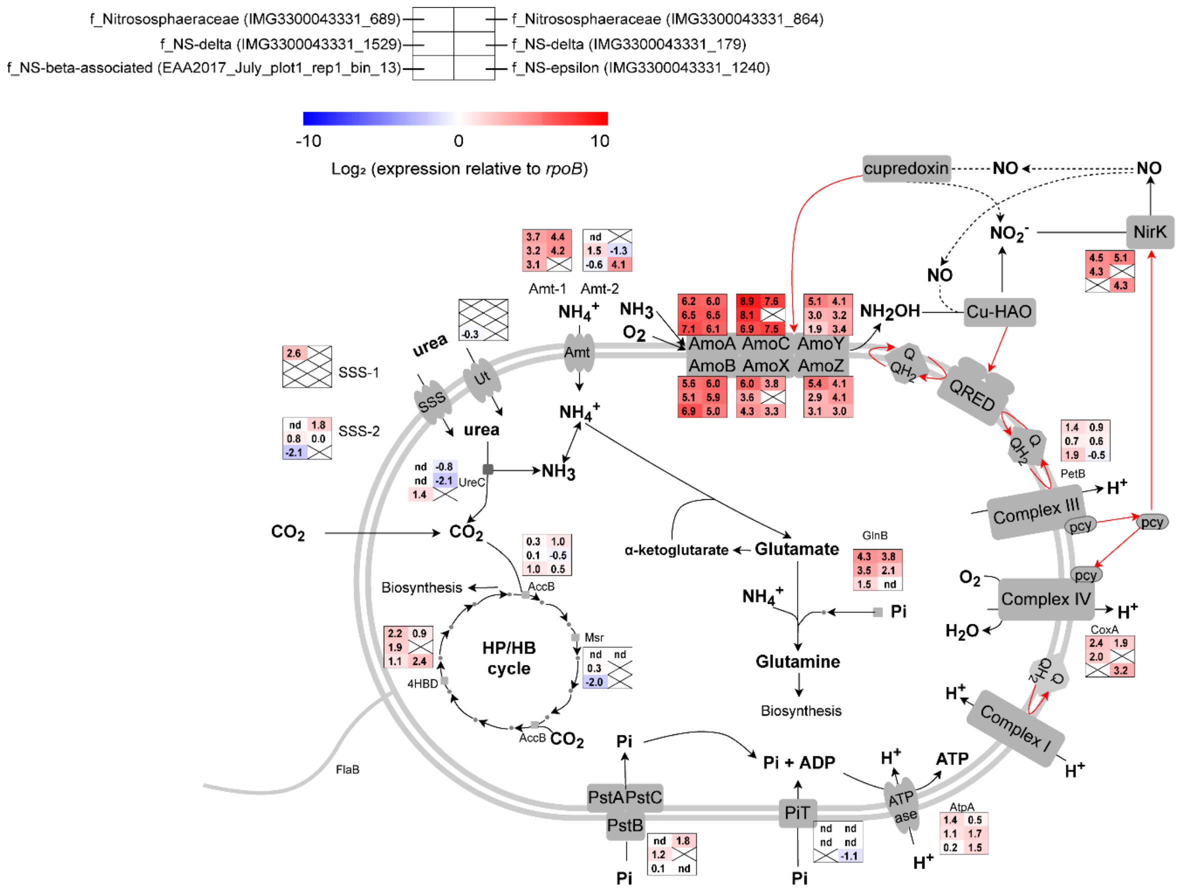

Fig. S32. **The *rpoB*-normalized transcript abundance of core metabolism pathway genes in in six MAGs assembled from EAA soils.** The cell diagram shows the core AOA metabolic pathways and proteins for ammonia transport, oxidation and assimilation, electron transfer, HP/HB carbon fixation and ATP synthesis. Heatmap shows log_2-transformed ratios of target gene transcript abundance to *rpoB* transcript abundance within each lineage averaging different plots and seasons. Cross symbol "X" indicates gene absent in a lineage or strain; "nd" indicates no transcript detected. Red arrows denote electron flow and dashed arrows indicate proposed pathways yet to be experimentally verified. AmoABCXYZ, ammonia monooxygenase subunit A, B, C, X, Y, and Z; NirK, copper-containing nitrite reductase; AccB, acetyl-CoA carboxylase beta; Msr, Malonic semialdehyde reductase; 4HBD, 4-hydroxybutyryl-CoA dehydratase; CoxA, Cbb3-type cytochrome c oxidase subunit I; PetB, putative cytochrome b/b6 domain protein; Amt-1 and Amt-2, type 1 and 2 ammonium transporters; SSS-1 and SSS-2, type 1 and 2 sodium-solute symporters; Ut, urea transporter; UreC, urease subunit alpha; GlnB, nitrogen regulatory protein P-II; PstB, phosphate transporter ATP-binding protein; PiT, low affinity inorganic phosphate transporter; AtpA, ATP synthase subunit A.
